# The single-cell transcriptional landscape of innate and adaptive lymphocytes in pediatric-onset colitis

**DOI:** 10.1016/j.xcrm.2023.101038

**Published:** 2023-05-08

**Authors:** Efthymia Kokkinou, Tea Soini, Ram Vinay Pandey, Aline van Acker, Jakob Theorell, Paulo Czarnewski, Egle Kvedaraite, Niels Vandamme, Magda Lourda, Chiara Sorini, Whitney Weigel, Anna Carrasco, Christopher Andrew Tibbitt, Heinrich Schlums, Ulrik Lindforss, Caroline Nordenvall, Malin Ljunggren, Maja Ideström, Mattias Svensson, Jan-Inge Henter, Eduardo J. Villablanca, Yenan T. Bryceson, Helena Rolandsdotter, Jenny Mjösberg

**Affiliations:** 1 Center for Infectious Medicine, Department of Medicine Huddinge, Karolinska Institutet, Karolinska University Hospital Huddinge, Stockholm, Sweden; 2 Center for Hematology and Regenerative Medicine, Department of Medicine, Karolinska Institutet, Karolinska University Hospital Huddinge, Stockholm, Sweden; 3 Psychiatry Southwest, Health Care Services Stockholm County, Huddinge, Sweden; 4 Science for Life Laboratory, Department of Biochemistry and Biophysics, National Bioinformatics Infrastructure Sweden, Stockholm University, Solna, Sweden; 5 Childhood Cancer Research Unit, Department of Women’s and Children’s Health, Karolinska Institutet, Karolinska University Hospital, Stockholm, Sweden; 6 Department of Clinical Pathology and Cancer Diagnostics, Karolinska University Hospital, Stockholm, Sweden; 7 Data Mining and Modeling for Biomedicine, VIB Center for Inflammation Research, Ghent, Belgium; 8 Department of Applied Mathematics, Computer Science and Statistics, Ghent University, Ghent, Belgium; 9 Immunology and Allergy Unit, Department of Medicine, Solna, Karolinska Institutet and University Hospital, Stockholm, Sweden; 10 Department of Molecular Medicine and Surgery, Karolinska Institutet and Center for Digestive Diseases, Karolinska University Hospital, Stockholm, Sweden; 11 Pediatric Gastroenterology, Hepatology and Nutrition Unit, Astrid Lindgren Children’s Hospital, Karolinska University Hospital, Stockholm, Sweden; 12 Theme of Children’s Health, Karolinska University Hospital, Stockholm, Sweden; 13 Department of Gastroenterology, Sachs' Children and Youth Hospital, Stockholm, Sweden; 14 Department of Clinical Science and Education, Södersjukhuset, Karolinska Institutet, Stockholm, Sweden

**Keywords:** ILC, IBD, pediatric inflammatory bowel disease, gut immunity, single-cell RNA sequencing, tissue-resident T cells

## Abstract

Innate lymphoid cells (ILCs) are considered innate counterparts of adaptive T cells; however, their common and unique transcriptional signatures in pediatric inflammatory bowel disease (pIBD) are largely unknown. Here, we report a dysregulated colonic ILC composition in pIBD colitis that correlates with inflammatory activity, including accumulation of naive-like CD45RA^+^CD62L^−^ ILCs. Weighted gene co-expression network analysis (WGCNA) reveals modules of genes that are shared or unique across innate and adaptive lymphocytes. Shared modules include genes associated with activation/tissue residency, naivety/quiescence, and antigen presentation. Lastly, nearest-neighbor-based analysis facilitates the identification of “most inflamed” and “least inflamed” lymphocytes in pIBD colon with unique transcriptional signatures. Our study reveals shared and unique transcriptional signatures of colonic ILCs and T cells in pIBD. We also provide insight into the transcriptional regulation of colonic inflammation, deepening our understanding of the potential mechanisms involved in pIBD.

## Highlights

• Innate lymphoid cell (ILC) balance is altered in pediatric IBD (pIBD)
• Colonic ILCs and T cells have shared and unique transcriptional programs
• PIBD inflammation is characterized by transcriptional changes in both ILCs and T cells

## Graphical abstract

**Figure:**
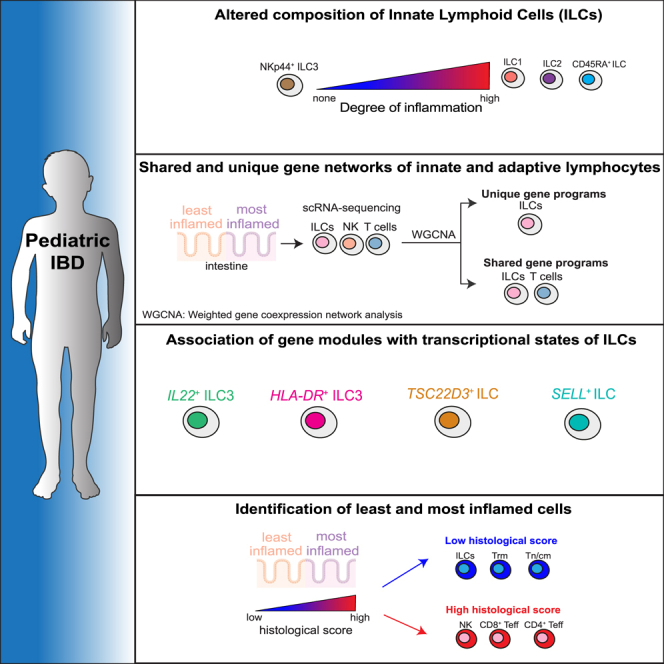

## Teaser

The role of innate lymphoid cells (ILCs) in pediatric IBD (pIBD) remains understudied. Here, Kokkinou et al. show that while some transcriptional features of colonic ILCs in pIBD are unique, some are shared with T cells. pIBD inflammation leads to transcriptional and cellular changes in both ILCs and T cells.

## Introduction

Pediatric inflammatory bowel disease (pIBD) includes Crohn’s disease (CD) and ulcerative colitis (UC), which have common symptoms like abdominal pain, frequent bloody diarrhea, and, for children with CD, sometimes also growth retardation.^1^ Around 25% of patients with IBD have been diagnosed before the age of 18 years, and the overall prevalence of pIBD is increasing.^2^ PIBD is characterized by more severe symptoms and a more extensive disease at onset than adults, and the CD phenotype shows less isolated ileal manifestations and more colonic inflammation compared with adult patients with CD.^2^ The etiology and pathogenesis of IBD is considered multifactorial. Host genetic as well as environmental factors may result in a dysregulated immune response and dysbiosis that is believed to drive chronic inflammation.^3^

T cells are implicated in intestinal inflammation in both mouse models^4^ and in humans, where conventional (CD4^+^ and CD8^+^)^5^^,^^6^^,^^7^ as well as unconventional T cells, including innate-like, γδ T cells and mucosal invariant (MAIT)^8^^,^^9^ T cells, show dysregulated composition. In patients with IBD, biologic treatments antagonizing immunomodulating factors that either activate T cells (interleukin [IL]-12/23, ustekinumab, etc.) or are expressed by T cells (α4β7 integrin; velodizumab, tumor necrosis factor [TNF]; infliximab, etc.) are effective in the majority of the patients.^10^

Innate lymphoid cells (ILCs), including natural killer (NK) cells, are viewed as the counterparts of adaptive T cells, which, in concert, execute specialized effector programs.^11^ In the inflamed adult IBD colon and ileum, the mucosal ILC composition is altered, resulting in reduced frequencies of IL-22-producing NKp44^+^ type 3 ILCs (ILC3s) and increased interferon (IFN)-γ-producing ILC1s^12^^,^^13^ as well as IL-17-producing ILC3s.^14^ However, it remains unknown whether these alterations in ILC frequencies are also present in pIBD.

Single-cell RNA sequencing (scRNA-seq) studies have provided transcriptional signatures of T cells in IBD mucosa of children and adults.^5^^,^^15^^,^^16^^,^^17^^,^^18^^,^^19^ However, characterization of intestinal ILCs in parallel to T cells in pediatric patients with IBD has not yet been performed.

Here, we provide a comprehensive characterization of ILCs and T cells in colonic biopsies from newly diagnosed and treatment-naive pediatric patients with IBD colitis and non-IBD controls. Flow cytometry showed inflammation-specific alterations of ILC frequencies, including the accumulation of CD45RA^+^CD62L^−^ naive-like ILCs, which correlated with disease severity. scRNA-seq revealed gene co-expression networks that were shared between or unique to innate and adaptive lymphocytes. Finally, a nearest-neighbor-based approach provided unbiased means to identify the “most inflamed” or “least inflamed” lymphocytes in our dataset based on the histological inflammation score of the samples. Overall, our study provides a comprehensive immunophenotypic and transcriptional characterization of ILCs and T cells in pediatric patients with IBD colitis. We uncover previously unknown co-regulatory dynamics of innate and adaptive lymphocytes that potentially play a role in disease pathogenesis and may be targeted for therapeutic intervention.

## Results

### Dysregulated colonic ILC composition correlates with inflammatory activity in pIBD colitis

To characterize ILCs in pediatric patients with newly diagnosed IBD, we collected colonic biopsies from endoscopically inflamed and non-inflamed sites from 19 treatment-naive patients with CD or UC colitis (Table S1). Pediatric patients who underwent colonoscopy because of intestinal symptoms but did not have IBD or other endoscopic inflammation served as the non-IBD control group (n = 10). For the patients with IBD, endoscopic inflammation was evaluated with the SWIBREG^20^ local endoscopic disease activity score (denoted “endoscopic score”) (Table S2), and local microscopic inflammation was evaluated with a modified version of the D′ Haens et al. histological score (denoted “histo score”; see STAR Methods).^21^

**Figure 1:**
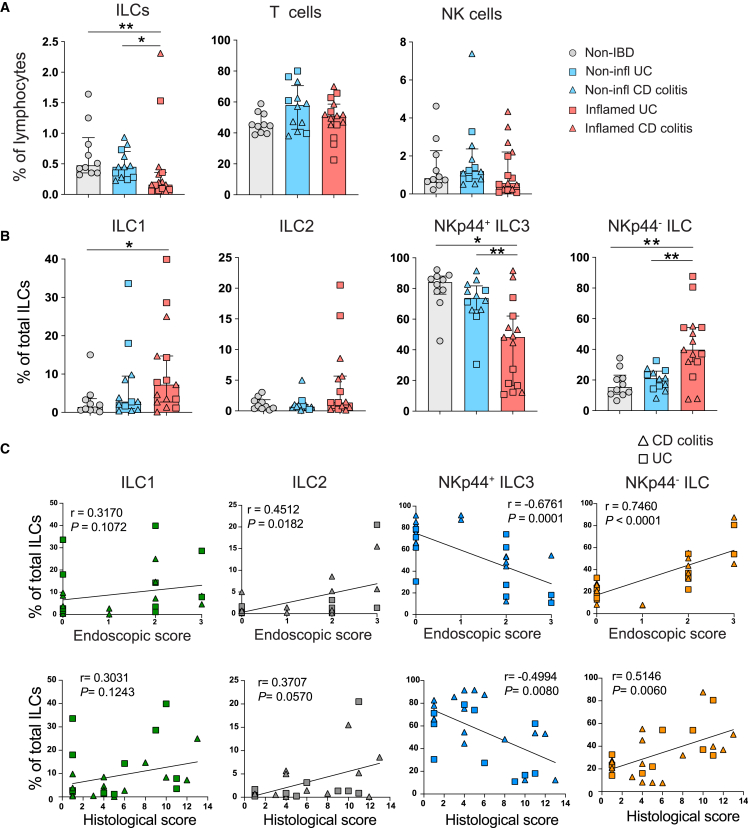
Dysregulated colonic ILC composition correlates to inflammatory activity in pIBD colitis (A) Bar graphs showing the frequencies of total ILCs, T cells, and NK cells (gated as shown in Figure S1A) in non-IBD (n = 10), endoscopically non-inflamed (n = 12), and endoscopically inflamed (n = 15) pIBD colon samples. A total of 8 samples were derived from paired inflamed and non-inflamed samples. Frequencies shown out of CD45^+^ lymphocytes. (B) Bar graphs showing the frequencies of ILC1s, ILC2s, NKp44^+^ ILC3s, and NKp44^−^ ILCs (gated as shown in Figure S1A) in non-IBD (n = 10), endoscopically non-inflamed (n = 12), and endoscopically inflamed (n = 15) pIBD colon samples. Frequencies shown out of total ILCs. (C) Correlation plots between frequencies of ILC1s, ILC2s, NKp44^+^ ILC3s, and NKp44^−^ ILCs and the endoscopic and histological scores determined for each of the endoscopically non-inflamed (n = 12) and endoscopically inflamed (n = 15) pIBD colon samples. Statistical significances in (B) were detected with Mann-Whitney test. Bar graphs are shown as median ± interquartile range (IQR). Spearman’s rank correlation test was applied for assessing correlations between variables. In (A)–(C), cells from each donor were analyzed in independent experiments.

Human intestinal CD127^+^ ILCs were analyzed by flow cytometry as previously described^13^^,^^22^ (Figure S1A). The identity of ILC1s, ILC3s, and NK cells was confirmed by staining of lineage-defining transcription factors in both blood and colon samples (Figure S1B). In contrast to CD3^+^ T cells and CD56^+^ NK cells, the frequency of CD127^+^ ILCs among lymphocytes was reduced in endoscopically inflamed IBD compared with non-IBD and non-inflamed IBD colon mucosa (Figure 1A). The majority of ILCs expressed the tissue residency marker CD69 (Figure S1C) but also showed increased expression of markers associated with naive T cells, CD45RA and CD62L (Figure S1C). While HLA-DR expression was elevated in pIBD ILCs, the expression remained unchanged in total CD3^+^ T cells (Figure S1C). However, elevated HLA-DR expression has been previously detected in CD4^+^ T cells in IBD.^5^ Further phenotyping of pIBD T cells and NK cells can be seen in Figure S1C.

We detected a skewed ILC composition in endoscopically inflamed IBD versus non-IBD and non-inflamed IBD colon, as illustrated by increased frequencies of NKp44^−^ ILCs and ILC1s, while NKp44^+^ ILC3 frequencies were reduced (Figure 1B), similar to our previous findings in adult IBD.^13^ There was a positive correlation between the endoscopic and histo scores and the frequencies of ILC2s and NKp44^−^ ILCs, whereas the frequency of NKp44^+^ ILC3s was inversely correlated with both endoscopic and histo scores (Figure 1D). Overall, our findings show that the mucosal ILC composition is dysregulated in patients with pIBD, displaying a pattern similar to what we previously reported for adult IBD.

### Single-cell transcriptional profiling of colonic ILCs, NK cells, and T cells in pIBD colitis

**Figure 2:**
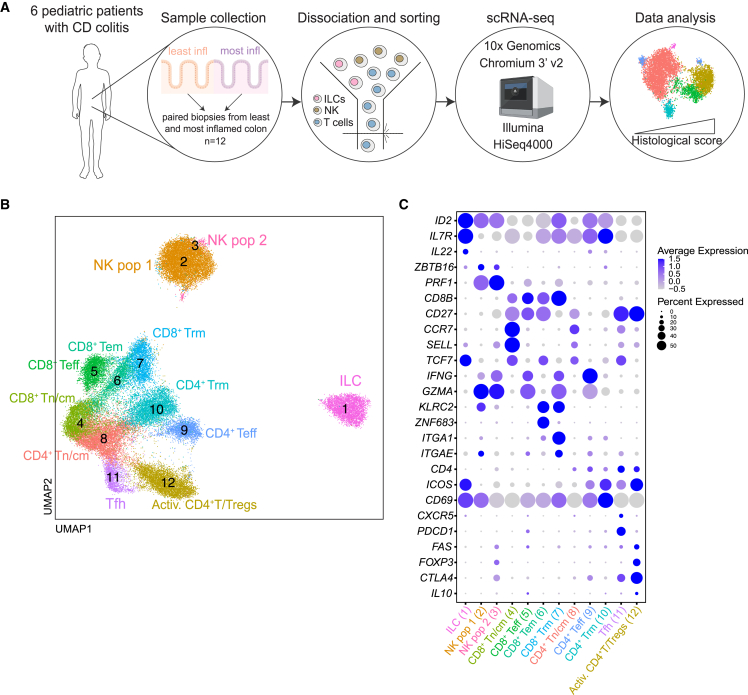
Single-cell transcriptional profiling of colonic ILCs, NK cells, and T cells in pIBD colitis (A) Schematic illustration of the scRNA-seq workflow of the total 12 paired colonic biopsy samples obtained from the endoscopically least and most inflamed areas of the 6 pediatric patients with CD-colitis (n = 6 donors). (B) UMAP visualization of the scRNA-seq data showing the unbiased clustering of total 44 717 ILCs, NK cells, and T cells. (C) Dotplot displaying expression of lineage-identity genes for the annotation of the clusters in (B). In (A)–(C), cells from each donor were analyzed in independent experiments.

To characterize the transcriptional signatures of both innate and adaptive lymphocytes in pIBD, we performed 3′ 10× scRNA-seq of biopsies from six treatment-naive pediatric patients with newly diagnosed CD-colitis (total n = 12 samples) (Figure 2A; Table S3). To assess the transcriptional signatures of inflammation within the same patient, biopsies were obtained from the endoscopically least and most inflamed area of the colon and subsequently fluorescence-activated cell sorted (FACS) for Lin^−^CD127^+^CD161^+^ (ILCs), Lin^+^CD127^−^CD56^+^ (NK cells), and CD3^+^ (T cells) (Figure S2A). After quality control, normalization, and integration of the datasets, 44,717 lymphocytes were obtained for analysis. Louvain clustering analysis allowed for the separation of the pre-sorted cell populations into 12 clusters, which were represented by all patients and samples (Figures 2B and S2B). Clusters were annotated based on signature genes (Figure 2C), where one cluster represented ILCs expressing *IL7R*, *ID2*, and *IL22*, two clusters represented different activation stages of NK cells expressing *ID2*, *ZBTB16*, *PRF1*, *GZMA*, and *IFNG*, and nine clusters represented subsets of CD4^+^ and CD8^+^ T cells. These included naive/central memory CD8^+^ and CD4^+^ T cells (CD8^+^ and CD4^+^ Tn/cm cells; *CD8*, *CD4*, *CCR7*, *SELL*, *TCF7*, and *CD27*); CD8^+^ T effector cells (CD8^+^ Teff cells; *CD8B*, *PRF1*, *GZMA*, and *IFNG*); CD8^+^ T effector memory cells (CD8^+^ Tem cells; *CD8B*, *KLRC2*, and *ZNF683*); CD8^+^ T resident memory cells (CD8^+^ Trm cells; *CD69*, *ITGA1*, and *ITGAE*); CD4^+^ Teff cells (*CD4*, *IFNG*, and *GZMA*); CD4^+^ Trm cells (*CD4*, *CD69*, and *ICOS*); T follicular helper cells (Tfh cells; *CD4*, *CXCR5*, *PDCD1*, and *FAS*); and activated CD4^+^T/regulatory T cells (Activ. CD4^+^T/Tregs; *CD4*, *CTLA4*, *ICOS*, *FOXP3*, and *IL10*) (Figures 2B and 2C).

Altogether, scRNA-seq revealed an overall diverse landscape of innate and adaptive lymphocytes in the pediatric colonic mucosa.

### Gene module analyses of colonic innate and adaptive lymphocytes reveal subset-specific and shared programs of co-expressed genes

**Figure 3:**
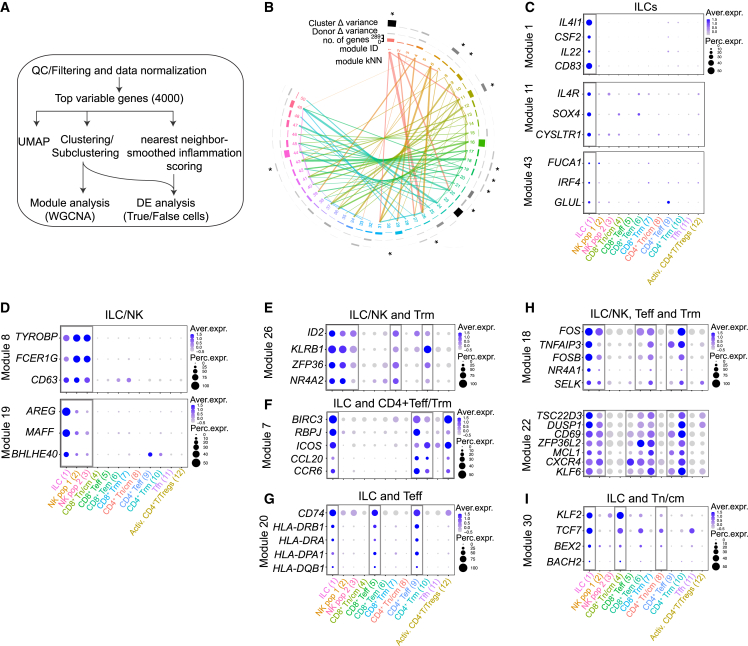
Gene module analyses of colonic innate and adaptive lymphocytes reveal subset-specific and shared programs of co-expressed genes (A) Summary flow chart used for the analysis of scRNA-seq data and subsequent gene module and nearest-neighbor analysis. (B) Circular dendrogram illustrating the number and composition of each module and the contribution of each module to discriminating cell clusters and donors. Connecting lines represent modules with highly correlated expressions (kNN [k nearest neighbor]). Modules selected for analysis and described in the text are marked with an asterisk. (C–I) A selection of genes in each module marked with an asterisk in (B) illustrated by dotplots. The expression of the modules across the annotated clusters is shown in Figure S3.

Complex biological processes like inflammation are orchestrated by co-expressed gene networks. In addition, ILCs and T cells largely overlap in terms of phenotype and functions, suggesting coordinated gene regulation. To identify such patterns of gene expression and regulation dynamics, we performed weighted gene co-expression network analysis (WGCNA),^23^^,^^24^ where we used the top 4,000 variable genes to discern 50 modules of co-regulated genes among the lymphocytes (Figure 3B). With this approach, we identified gene networks that were either confined to a specific cell type or shared across cell types. More specifically, we identified three modules (1, 11, and 43) preferentially expressed by the ILC cluster (Figures 3C and S3A). Intriguingly, module 1 showed coordinated expression between key ILC cytokine genes *IL22*, *CSF2* (encoding GM-CSF), the co-stimulatory molecule *CD83*, and *IL4I1*, the latter encoding a secreted L-amino acid oxidase reported to promote aryl hydrocarbon receptor (AHR) activation while limiting Th17 proliferation.^25^^,^^26^ The expression of *SOX4*, *IL4R*, and *CYSLTR1* (encoding the cysteinyl leukotriene receptor) in module 11 showed that although a distinct cluster of ILC2s could not be identified in our data, ILCs expressing transcripts with known regulatory function in ILC2s/Th2 cells are present in the pIBD colon.

Two gene modules were shared between the two innate lymphocyte clusters (ILCs and NK cells; modules 8 and 19), including the cytotoxicity-associated adapter proteins *TYROBP* and *FCER1G*, as well as the effector molecule *AREG* that was expressed together with *BHLHE40*, encoding a transcriptional regulator previously reported to control cytokine production in T cells^27^^,^^28^ (Figures 3D and S3B). Highlighting the shared features of ILCs and T cells, module 26 was coordinately expressed by ILCs and Trm cells, including innate-like genes (*ID2* and *KLRB1*) and genes associated with tissue residency (*ZFP36* and *NR4A2*) (Figures 3E and S3C). Module 7 was shared by ILCs and CD4^+^ Teff cells, Trm cells, and Activ. T cells and included genes typically expressed by Th17 cells (*RBPJ*, *ICOS*, *CCL20*, and *CCR6*) (Figures 3F and S3D). In line with previous reports showing antigen-presenting features of ILCs,^29^^,^^30^^,^^31^^,^^32^ module 20 contained a set of transcripts associated with antigen presentation (*HLA-DRA* and *HLA-DPA1*) that were shared by ILCs and Teff cells (Figures 3G and S3E). Additional shared signatures between ILCs and T cells were related to genes that regulate tissue residency (*FOS* and *NR4A1*; module 18) as well as quiescence (*TSC22D3*, *DUSP1*, *ZFP36L2*, and *KLF6*; module 22) (Figures 3H and S3F), the latter recently shown to be expressed by plastic skin ILCs in a mouse model of psoriasis.^33^ Lastly, module 30 showed coordinated expression of *KLF2* and *TCF7*, genes typically expressed by naive T cells. However, module 30 was highly expressed by ILCs and naive T cells, indicating the presence of ILCs with naive features as previously described in the adult gut^24^^,^^34^ (Figures 3I and S3G).

Overall, these findings reveal subset-specific and common gene signatures that may regulate ILCs and T cells.

### Gene modules associate with ILC subclusters and identify quiescent/naive-like ILCs

**Figure 4:**
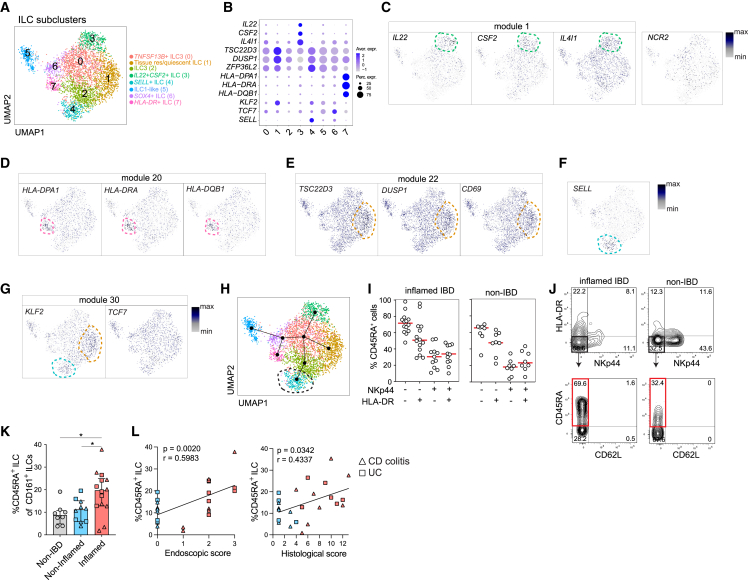
Gene modules associate with ILC subclusters and identify quiescent/naive-like ILCs (A) UMAP visualization of ILC subclusters after reclustering main cluster 1 shown in Figure 2B. (B) Dotplot showing selected ILC-module genes (Figures 3D–3I). The expression is shown within the annotated subclusters of ILCs depicted in (A). (C–G) UMAP visualization of selected ILC-module genes in the ILC subclusters. Dotted lines mark the subcluster where the depicted gene is highly and differentially expressed. A list of differentially expressed genes (DEGs) per ILC subcluster is shown in Figure S4A. (H) Developmental trajectories of ILC subclusters performed with Monocle 3. Subcluster 4 (dotted circle) was used as the root. (I) Frequencies of CD45RA^+^ ILCs among ILCs expressing combinations of HLA-DR and NKp44 in inflamed pIBD (n = 12–14) and non-IBD (n = 8) colon biopsies (bar graphs are shown as median). The samples were analyzed in independent experiments. (J) Representative flow cytometry plots of CD45RA versus CD62L (marked in red is the CD45RA^+^ ILC subset), gated out of NKp44^−^HLA-DR^−^ ILCs, in inflamed pIBD and non-IBD colon biopsies. (K) Frequency of CD45RA^+^ ILCs out of CD161^+^ ILCs in non-ΙBD (n = 8), endoscopically non-inflamed (n = 10), and endoscopically inflamed pIBD (n = 14) colon biopsies. Mann-Whitney test (p < 0.05), mean with SD. The samples were analyzed in independent experiments. (L) Correlation plots (Spearman, p < 0.05) between the frequency of CD45RA^+^ ILCs and the endoscopic and histological scores determined for each of the endoscopically non-inflamed (n = 8) and endoscopically inflamed (n = 14) pIBD colon samples.

To better understand the expression pattern of unique and shared gene modules among ILCs, we focused our analysis only on the ILCs. Reclustering of ILCs revealed 7 transcriptionally distinct clusters that were annotated by differential expression (DE) analysis (Figures 4A, 4B, S4A, and S4B). Interestingly, the pIBD ILC subclusters displayed a similar degree of heterogeneity with ILCs from adult IBD colon, while tonsillar ILCs were less heterogeneous (Figures S4C and S4D). Intriguingly, the module 1 genes *CD83*, *IL4I1*, *IL22*, and *CSF2* were confined to ILC subcluster 3 (Figures 4C and S4E), but these genes associated poorly with expression of the key ILC3 genes *NCR2* (Figure 4C) and *RORC* (Figure S4F), which were widely expressed among the ILC clusters. We further identified that module 20 genes (*HLA-DPA1*, *HLA-DRA*, and *HLA-DQB1*) were uniquely expressed (Figures 4B, 4D, and S4E) by subcluster 7, suggesting that they represent antigen-presenting ILCs. Furthermore, subcluster 1 expressed many genes in modules 18 and 22, including *TSC22D3*, *DUSP1*, and *CD69* (Figures 4B, 4E, and S4E), previously associated with quiescent and tissue-resident ILCs.^33^ Interestingly, in line with our previous report,^34^ we identified a *SELL*-expressing ILC subcluster 4 (Figures 4B and 4F), with low expression of *NCR2*, *HLA-DR*, and *RORC* transcripts (Figures 4B, 4D, and S4F), likely representing naive-like ILCs enriched in adult IBD. Genes in the “naivety” module 30 (Figure 3I) were not solely expressed by subcluster 4. In fact, *TCF7* was homogenously expressed across all ILCs, and *KLF2* was also expressed by subcluster 1 (Figures 4B and 4G). These data indicated the presence of at least two subclusters of ILCs with features of naive T cells. To determine the developmental relationships between the ILC subclusters, we performed trajectory analysis with Monocle 3 using subcluster 4 (Figure 4H) or 1 (Figure S4G) as the root, respectively. We found that regardless of the root, subclusters 3 (*IL22*-expressing effector ILCs), 4 (naive ILCs), 1 (tissue resident/quiescent ILCs), and 6–8 (ILC1-like ILCs, HLA-DR^+^ ILCs, SOX4^+^ ILCs) branch separately from each other, with cluster 0 as the branching point.

To better characterize the naive/quiescent ILCs in pIBD colon, we stained ILCs for markers of activation/differentiation. We have recently described that the less-differentiated and less-activated (NKp44^−^HLA-DR^−^) ILCs in human tissues, including adult IBD gut, form a heterogeneous population that includes naive-like/quiescent ILCs marked by the presence of surface CD45RA expression.^34^ Confirming and extending our previous findings, CD45RA also marked such cells in pIBD (Figure 4I). As we observed in the adult IBD gut, surface CD62L was not expressed on this population despite the presence of *SELL* transcripts (Figure 4J). Similarly to adult IBD, the frequency of these CD45RA^+^CD62L^−^ ILCs (referred to as CD45RA^+^ ILCs) was increased in the endoscopically inflamed pIBD gut samples, and their frequency was positively correlated with the local endoscopic as well as histo score (Figures 4K and 4L).

In summary, our analyses reveal gene networks confined to certain ILC subsets, including a naive-like module. Using previously described surface markers that identify naive-like/quiescent ILCs in tissues, we observed an accumulation of CD45RA^+^ ILCs in the inflamed gut of pediatric patients with IBD that correlated with the degree of inflammation.

### Nearest-neighbor-smoothed histo scores reveal the most and least inflammatory cells

**Figure 5:**
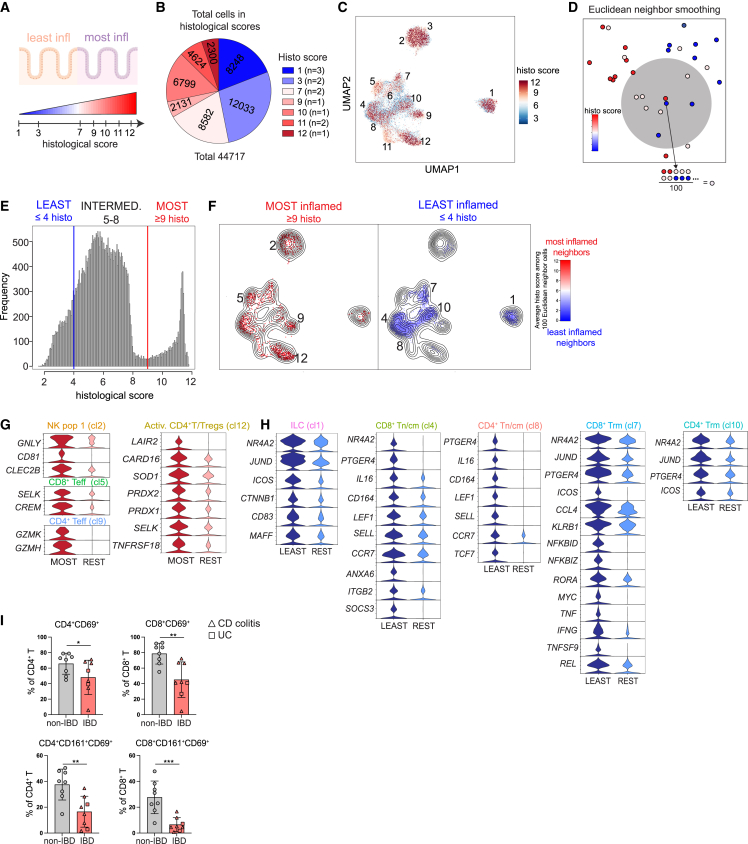
Nearest-neighbor-smoothed histological scores reveal the most and least inflammatory cells (A) Schematic illustration showing the histological scores from the endoscopically least and most inflamed paired colonic biopsies used for scRNA-seq. (B) Pie chart showing the total number of cells from areas of different histological scores from all 12 pIBD colon biopsy samples. (C) The raw histological score of each cell derived from each sample depicted onto the main UMAP from Figure 2B. (D) Schematic illustration of the Euclidean neighbor smoothing. Each cell was given the average histological scores of its 100 transcriptionally closest neighbors. These smoothed histological scores were used for the subsequent analysis. (E) Histogram showing the cell type distribution across the histological scores derived from the nearest-neighbor analysis. Cells were categorized into 3 groups: ≤4 (least inflamed), 5–8 (intermediate), and ≥9 (most inflamed). (F) Visualization of cells from the nearest-neighbor smoothing on the main UMAP with cells derived from the ≥9 histological (histo) score (most inflamed) and ≤4 histo score (least inflamed) groups, which also have neighbors from at least 5 samples. (G and H) Violin plots showing the expression level of selected DEGs (log2 fold change [FC] > 0.10 and adjusted p value [adj. p value] < 0.05) upregulated in the most (G) and least (H) inflamed cells versus the rest of the cells in each cluster. (I) Frequencies of CD4^+^CD69^+^, CD4^+^CD161^+^CD69^+^, CD8^+^CD69^+^, and CD8^+^CD161^+^CD19^+^ cells in colonic adult non-IBD (n = 8) and IBD samples (n = 8). Mann-Whitney test (p < 0.05), mean with SD. The samples were analyzed in two independent experiments.

Next, we set out to explore inflammation-related transcriptional signatures of innate and adaptive lymphocytes. During endoscopy, the grade of inflammation is evaluated based on the presence of visual signs.^35^ In contrast, histological findings more precisely assess both the acute and chronic cellular changes occurring in the intestinal epithelium and lamina propria.^36^ We were therefore interested in defining the transcriptional signatures of these cellular changes with scRNA-seq (Figures 5A and 5B). Indeed, when plotting the histo scores on the uniform manifold approximation and projection (UMAP), we could observe overrepresentation of lymphocytes from samples with high histo scores in certain clusters (Figure 5C). However, this crude representation did not consider donor representability. Therefore, we repurposed a nearest-neighbor-based smoothing method deployed elsewhere.^37^^,^^38^^,^^39^ Here, each cell was assigned the average histo scores of the 100 nearest-neighbor cells in the Euclidean space, which was created by a 100-dimensional principal-component analysis based on the 4,000 most variable genes (Figure 5D). To balance out the number of neighbors for each histological group (defined below), we conducted subsampling of the data so that all three groups consisted of the same number of neighbors (Figure S5A). Distribution of lymphocytes across tissues with different histo scores allowed for categorization of the cells into three groups: least inflamed (histo score ≤ 4), “intermediate” (histo score = 5–8), and most inflamed lymphocytes (histo score ≥ 9) (Figure 5E). Visualization of the smoothed histo scores revealed enrichment of the most inflamed and least inflamed lymphocytes in certain clusters (Figures 5F and S5B). The most inflamed samples were mostly derived from 4 samples (Figure S5C). However, similar enrichment was predicted when batch correction was applied (Figure S5D) and also when we performed differential abundance analysis with DAseq (Figure S5E).^38^ The most inflamed lymphocytes were predominantly NK cells (cluster 2), CD8^+^ Teff cells (cluster 5), CD4^+^ Tef cells (cluster 9), and Activ. T cells/Tregs (cluster 12) (Figure 5F). Conversely, the least inflamed cells were enriched in ILCs (cluster 1), CD8^+^ Tn/cm cells (cluster 4), CD4^+^ Tn/cm cells (cluster 8), CD8^+^ Trm cells (cluster 7), and CD4^+^ Trm cells (cluster 10).

To uncover transcriptional signatures of the most inflamed lymphocytes, we performed DE analysis in the most inflamed cells (shown as MOST) versus the rest of the cells (shown as REST) within each cluster. A total of 96 genes were upregulated in the most inflamed cells with 35 genes being shared among the clusters (Table S4). The most inflamed NK cells showed elevated expression of the transcript for the antimicrobial molecule granulysin (*GNLY*) and genes involved in immunoregulatory interactions (*CD81* and *CLEC2B*), while the most inflamed CD8^+^ T cells expressed the selenoprotein K (*SELK*) and the cAMP regulator *CREM* (Figure 5G). Interestingly, the most inflamed CD4^+^ Teff cells displayed a cytotoxic signature with expression of the granzymes *GZMK* and *GZMH* (Figure 5G), while the most inflamed Activ. CD4^+^ T/Tregs upregulated genes involved in redox balance (*SELK*, *PRDX1*, *PRDX2*, and *SOD1*), the caspase inhibitor *CARD16*, and the inhibitory molecules *LAIR2* and *TNFRSF18* (GITR) (Figure 5G). Similarly, to transcriptionally characterize the least inflamed lymphocytes, we performed DE analysis in the least inflamed cells (shown as LEAST) versus the rest of the cells (shown as REST) (Figure 5H). The least inflamed lymphocytes demonstrated larger transcriptional differences compared with the most inflamed lymphocytes with an upregulation of 226 genes, of which 70 were shared among the clusters (Table S4). Transcripts related to tissue residency such as *NR4A2* and *JUND* as well as *PTGER4* and *ICOS* were commonly expressed by the least inflamed cells across several clusters (Figure 5H). Additionally, the least inflamed ILCs revealed expression of *CD83*, involved in dendritic cell maturation as well as lymphocyte activation.^40^ The least inflamed CD8^+^ and CD4^+^ Tn/cm cells expressed molecules related to their identity (*SELL*, *LEF1*, *CCR7*, *TCF7*) like the least inflamed Trm cells that additionally showed overexpression of genes associated with tissue residency (*ICOS*, *JUND*, *NR4A2*, and *PTGER4*) (Figure 5H). The least inflamed CD8^+^ Trm cells additionally upregulated genes related to “innateness” (*RORA*, *KLRB1*) and effector functions (*IFNG*, *TNF*, *CCL4*) (Figure 5H). To validate these findings, we performed flow cytometry using intestinal specimens of adult patients with and without IBD (Table S5). We showed that, indeed, CD4^+^ Trm and CD8^+^ Trm cells (defined as CD69^+^ and/or CD161^+^) were lower in frequency in IBD, as predicted by the nearest-neighbor analysis (Figure 5I). The prediction of ILCs being mostly represented in the least inflamed landscape was additionally confirmed by our flow cytometry analysis of total ILCs (Figure 1A).

Collectively, based on nearest-neighbor analysis of transcriptional profiles and histo scores, we identified most inflamed and least inflamed lymphocytes with potential roles in colonic inflammation. The genes expressed by these cells deserve further functional exploration to assess their usefulness as targets for immunotherapy in IBD.

## Discussion

In our study, we combined high-dimensional single-cell transcriptional and immunophenotypic analyses to comprehensively characterize innate and adaptive lymphocytes from colonic biopsies obtained from treatment-naive pediatric patients with newly diagnosed IBD colitis. We focused on pIBD as it generally shows a more aggressive phenotype and allows for studies of disease mechanisms that are less influenced by diagnostic delay,^41^ age-related environmental exposures, for example smoking, and co-morbidities compared with adults. A number of studies have investigated IBD-associated patterns of T cells and ILCs in human tissues at the single-cell level.^5^^,^^15^^,^^16^^,^^18^^,^^19^ However, these studies describe discrete cell populations of either ILCs or T cells without directly comparing these two cell types. Therefore, we complemented our protein analysis of ILCs with in-parallel single-cell transcriptional profiling of ILCs, NK cells, and T cells to uncover transcriptional networks that govern both shared and unique properties. Through our approach, we provide insights into ILC and T cell biology in pIBD. Understanding the regulation of these transcriptional networks could possibly lead to the development of therapies for IBD.

Flow cytometry revealed a dysregulated composition of mucosal ILCs in pIBD that correlated with the inflammatory activity. More specifically, we observed a reduction of NKp44^+^ ILC3s and an increase of NKp44^−^ ILCs and ILC1, the latter which could, at least to some extent, be explained by ILC3-ILC1 plasticity as described for adult IBD.^12^ However, it is currently unclear how well the FACS-based definition of human ILCs correlates with transcriptional and functional features of ILCs in the gut mucosa. Similar to our FACS definition of ILC1, scRNA-seq analysis revealed an ILC1-like cluster expressing *TBX21* (encoding T-bet) but low *KIT* (encoding CD117) and *RORC*. These cells likely represent the previously described CD127^+^ ILC1s^12^ and not the intraepithelial CD103^+^ ILC1s, as those lack CD127 expression.^42^ Expression of *NCR2*, encoding NKp44, was seen in several ILC subclusters, indicating transcriptional heterogeneity among NKp44^+^ ILC3s. It has been well described that human NKp44^+^ ILC3s harbor IL-22-producing ILCs^43^ and that ILC3-derived IL-22 is crucial for gut homeostasis in mice.^44^ However, here we show that *IL22* transcription is confined to a specific ILC subcluster with relatively low expression of *RORC*. This is biologically possible as RORC-deficient patients are still capable of IL-22 production.^45^ Of note, *IL22* was co-transcribed with other effector molecules such as *CSF2*, *CD83*, and *IL4I1* in a gene module that was preferentially expressed in ILCs compared with T cells, potentially reflecting the more rapid response of ILCs compared with T cells. Notably, *IL4I1* can activate AHR ligands in tumor cells via tryptophan metabolism,^25^ which in turn supports IL-22 production in ILCs.^46^ Thus, *IL4I1* may promote AHR in ILC3s. Additional studies are needed to investigate how *IL4I1* or other co-expressed molecules regulate IL-22 production by intestinal human ILCs*.*

Although ILC2s represent a large population in the mouse intestine^47^ and human fetal intestine,^48^ the healthy human adult intestine is largely devoid of conventional CD127^+^ ILC2s,^48^^,^^49^ similar to what we also observed in our flow cytometric and scRNA-seq analysis of pIBD colon tissue. Although it is possible that intestinal ILC2s show an unconventional profile that we excluded with our current sorting gating strategy, we identified an ILC-restricted gene module containing genes associated with the regulation of ILC2s. This included positive regulators such as *IL4R* and *CYSTL1* but also *SOX4*, which can suppress Th2 differentiation.^50^ Thus, we speculate that the absence of a transcriptionally distinct subset of CD127^+^ ILC2s in the human intestine could be due to gut environmental factors that suppress ILC2s with a conventional phenotype. This topic requires further investigation.

One of the most striking overlaps between innate and adaptive lymphocytes in our analysis was the expression of a gene module containing genes associated with tissue residency including *CD69*, *CXCR4*, and *KLF6*. This gene module was particularly highly expressed in ILCs and Trm cells, indicating similar programs for tissue residency in innate and adaptive lymphocytes, a topic that deserves further investigation. However, we also noted that this module included genes associated with cellular quiescence, *TSC22D3*, *DUSP1*, and *ZFP36L2*.^33^ These genes were highly transcribed in a *SELL*-expressing ILC subcluster (*ZFP36L2*) and in another ILC subcluster (*TSC22D3* and *DUSP1*), which co-transcribed the transcription factor *KLF2*, typically expressed by naive T cells. The *SELL*-expressing ILC subcluster lacked features of mature ILCs including *RORC*, *NCR2*, *TBX21*, or *EOMES*, reminiscent of the CD62L^+^ naive-like ILCs we recently described in tonsils.^34^ The second naive/quiescent cluster, which lacked expression of *TBX21* and *EOMES*, likely represented the quiescent/naive-like ILCs that we previously reported in human tonsil and adult gut,^34^ where we showed that these less-differentiated quiescent ILCs are marked by the presence of CD45RA expression and, despite expression of *SELL* transcripts, the absence of CD62L.^34^ The frequency of this subset was increased in the inflamed gut of adult patients with IBD, and they could differentiate to ILC1s and IL-22-producing ILC3s.^34^ In the present study, we extended this finding to show that CD45RA^+^ ILCs accumulate in the inflamed gut of pediatric patients with IBD as well. These cells could be ready for *trans*-differentiation, as previously shown in a mouse model of psoriasis where ILC3s accumulated by *trans*-differentiation of ILC2s via a quiescent-like state of ILCs.^33^

We further identified a gene module that was commonly expressed by a distinct subcluster of ILCs and T cells and that contained transcripts of major histocompatibility complex (MHC) class II molecules involved in antigen presentation. Indeed, we and others have previously shown that ILCs can acquire antigen-presenting properties that in turn can be suppressed by the human tumor microenvironment^29^ and the gut microenvironment in mice.^31^ However, the antigen-presenting capacity of ILCs in the context of human IBD, in the presence of a more complex microbiota compared with transgenic mice, remains underexplored. Hence, it is possible that ILCs could orchestrate CD4^+^ T cell responses during gut inflammation in humans, similarly to what was described in a mouse model of acute colitis.^30^

To unravel inflammation-driven transcriptional changes in innate and adaptive lymphocytes, we made use of the histological inflammation score of our samples rather than grouping them in two endoscopically defined groups with variable histo scores. A nearest-neighbor-based smoothing of histo scores identified so-called least inflamed cells that were mostly defined as ILCs, CD4^+^ and CD8^+^ Tn/cm cells, and innate-like CD8^+^ Trm cells. The least inflamed colon areas of patients with pIBD may represent tissue that was previously inflamed and now healed or tissue that is yet unaffected by inflammation. Regardless, the transcriptomes of least inflamed cells represent a footprint of innate and adaptive lymphocyte homeostasis in the colon. This includes genes associated with the lineage identity of ILCs, CD4^+^/CD8^+^ Tn/cm cells, CD4^+^ Trm cells, and innate-like CD8^+^ Trm cells, suggesting that maintained function of these cells is critical for immune homeostasis in the gut and that loss of these cells is associated with gut inflammation. In contrast, the most inflamed cells were dominated by NK cells, cytotoxic CD4^+^ T cells, Activ. CD4^+^ T cells/Tregs, and CD8^+^ Teff cells.

It is possible that the compositional differences in least and most inflamed cells is due to recruitment of lymphocytes from the blood to inflamed areas of the colon. However, it could also be due to plasticity and/or differentiation of cells. Plasticity of ILCs and T cells is well described, and for ILCs, conversion of non-cytotoxic human ILCs to NK cells has been demonstrated *in vitro*.^51^ Most inflamed NK cells upregulated transcripts of the antimicrobial protein *GNLY* and *CLEC2B*, the latter mediating NK cell activation via ligation to NKp80.^52^ *GNLY* has been shown to be released in response to infections and to facilitate antigen presentation in dendritic cells (DCs) via Toll-like receptor 4 (TLR4).^53^ Interestingly, *GNLY*-expressing CD94^+^CD127^+^ ILCs are expanded in patients with CD,^54^ illustrating the fluidity in ILC-NK cell functions and the possibly that ILCs are reduced at the expense of NK cells during gut inflammation. Similarly, the underrepresentation of CD4^+^ Tn/cm and CD4^+^ Trm cells among the most inflamed cells could be due to differentiation to cytotoxic CD4^+^ T cells and/or Activ. T cells/Tregs. Several studies have described the presence of cytotoxic CD4^+^ T cells (CD4^+^ CLT cells) in IBD,^55^^,^^56^ but their origin remains unknown. We identified a CD4^+^ T cell subset expressing *GZMH* and *GZMK* in inflamed IBD. Interestingly, this is in line with a recent report describing that CD4^+^ CTL cells exhibit a diverse granzyme expression in nasal polyp tissue, including a subset that expressed these non-conventional granzymes.^57^ GZMK and GZMH expression is seen in CD8^+^ T cells with limited TCR repertoire and expression of exhaustion markers in aging^58^ and autoimmunity,^59^ suggesting that the CD4^+^ T cell subset that we detect herein might be a highly differentiated/exhausted subset. Future studies should be aimed to address the origin and function of these CD4^+^ CLT cells and Activ. T cells/Tregs in the context of gut mucosal inflammation in IBD.

We also noted accumulation of CD8^+^ Teff cells among the most inflamed cells. While it is tempting to speculate that these cells could be derived from the CD8^+^ Tn/cm and/or CD8^+^ Trm cells among the least inflamed cells, further work is required to address this. The role for the CD8^+^ T cell compartment is conflicting in IBD. A single-cell atlas study of CD8^+^ T cells in UC described innate features of *CD8*^+^*IL26*^+^ T cells,^18^ while another study reported reduced frequencies of cytotoxic IFN-γ^+^ CD8^+^ T effector memory cells re-expressing CD45RA (TEMRA) cells in UC mucosa.^5^ Similarly, we found increased expression of *IFNG*, *TNF*, and innate-like transcripts in the least inflamed IBD CD8^+^ Trm cells, suggesting that these cells have important homeostatic functions in the gut.

In summary, we report that pediatric patients with IBD display dysregulated ILC frequencies that are similar to those observed in adult patients with IBD, suggesting a common pathogenic program in this aspect. Using a combination of high-dimensional single-cell analysis on the transcriptional and protein levels, we provide a roadmap of ILCs and T cells in pIBD, including transcriptional signatures associated with inflammation. Further mechanistic studies are necessary to understand whether and how these findings contribute to pathogenesis and how they could facilitate the design of immunotherapeutic targets.

### Limitations of the study

Although paired non-inflamed biopsies were used to infer inflammatory changes in the inflamed samples, the main limitation of the study is the lack of a non-symptomatic, healthy non-IBD control group in the single-cell analysis. Furthermore, one of the main transcriptional findings, on the reduced presence of Trm cells in colon inflammation, was validated on the protein level using samples from adult patients with IBD. However, we were not able to validate this, or other interesting transcriptional findings, in samples from patients with pIBD. Finally, since we performed our studies in samples from patients with ongoing inflammation, we cannot determine if the observed differences are a cause or consequence of IBD and what the role of these differences are in driving inflammation. In summary, the limitations of this study represent important aspects to address in future studies.

## STAR★Methods

### Key resources table

**Table:** 

REAGENT or RESOURCE	SOURCE	IDENTIFIER
**Antibodies**		

anti-human CD1a FITC (Clone HI149)	Biolegend	Cat#300104
anti-human CD14 FITC (Clone Tuk4)	LifeTech	Cat#MHCD1401-4
anti-human CD19 FITC (Clone 4G7)	BD	Cat#345776
anti-human CD123 FITC (Clone 6H6)	Biolegend	Cat#306014
anti-human BDCA2 FITC (Clone AC144)	Miltenyi	Cat#130-113-192
anti-human FcER1a FITC (Clone AER-27)	Biolegend	Cat#334608
anti-human CD34 FITC (Clone 581)	Biolegend	Cat#343504
anti-human CD94 FITC (Clone DX22)	Biolegend	Cat#305504
anti-human TCRab FITC (Clone IP26)	Biolegend	Cat#306706
anti-human TCRgd FITC (Clone B1)	Biolegend	Cat#331208
anti-human Eomes FITC (Clone WD1928)	eBioscience	Cat#11-4877-42
anti-human NKG2A (CD159a) APC (Clone Z199)	Beckman Coulter	Cat#60797
anti-human Eomes efluor660 (Clone WD1928)	eBioscience	Cat#50-4877-41
anti-human CD69 AF700 (Clone FN50)	Biolegend	Cat#310992
anti-human CD3 AF700 (Clone UCHT1)	BD	Cat#557943
anti-human CD62L APC-Cy7 (Clone DREG-56)	Biolegend	Cat#304814
anti-human CRTH2 V450 (Clone BM16)	BD	Cat#561661
anti-human CD45 V500 (Clone HI30)	BD	Cat#560777
anti-human CD1a biotin (Clone HI149)	Biolegend	Cat#300112
anti-human CD14 biotin (Clone HCD14)	Biolegend	Cat#325624
anti-human CD19 biotin (Clone HIB19)	Biolegend	Cat#302204
anti-human CD123 biotin (Clone 6H6)	Biolegend	Cat#306004
anti-human BDCA2 biotin (Clone GoH3)	Miltenyi	Cat#130-097-923
anti-human FcER1a biotin (Clone AER-37)	eBioscience	Cat#13-5899-82
anti-human CD34 biotin (Clone 581)	Biolegend	Cat#343524
anti-human CD94 biotin (Clone REA113)	Miltenyi	Cat#130-098-967
anti-human TCRab biotin (Clone IP26)	Biolegend	Cat#306704
anti-human TCRgd biotin (Clone B1)	Biolegend	Cat#331206
Streptavidin BV570	Biolegend	Cat#405227
anti-human CD90 BV605 (Clone 5E10)	Biolegend	Cat#328128
anti-human CD161 BV605 (Clone HP-3G10)	Biolegend	Cat#339915
anti-human CD45 BV650 (Clone HI30)	Biolegend	Cat#304043
anti-human CD56 BV650 (Clone HCD56)	Biolegend	Cat#318343
anti-human CD56 BV711 (Clone HCD56)	Biolegend	Cat#318336
anti-human CD3 BV785 (Clone OKT3)	Biolegend	Cat#317329
anti-human NKG2A (CD159a) PE (Clone 131411)	R&D	Cat#FAB1059P
anti-human ROR-gt PE (Clone AFKJS-9)	eBioscience	Cat#12-6988-82
anti-human HLA-DR PE (Clone L243)	eBioscience	Cat#12-9952-41
anti-human CRTH2 PE-CF594 (Clone BM16)	BD	Cat#563501
anti-human CD161 PE-CF594 (Clone HP-3G10)	Biolegend	Cat#339939
anti-human T-bet PE-CF594 (Clone O4-46)	BD	Cat#562467
anti-human NKp44 PE-Cy5 (Clone Z231)	Beckman Coulter	Cat#A66903
anti-human CD117 PE-Cy5.5 (Clone 95C3)	Beckman Coulter	Cat#A66333
anti-human CD127 PE-Cy7 (Clone R34.34)	Beckman Coulter	Cat#A64618
anti-human CD45 BUV395 (Clone HI30)	BD	Cat#563791
anti-human CD45RA BUV737 (Clone HI100)	BD	Cat#564442
anti-human CD3 BV570 (Clone UTCH1)	Biolegend	Cat#300436
anti-human CD69 BV650 (Clone FN50)	Biolegend	Cat#310934
anti-human CD4 BUV496 (Clone SK3)	BD	Cat#564652
anti-human CD8 BUV805 (Clone SK1)	BD	Cat#612889

**Biological samples**		

Intestinal biopsies from pediatric IBD and non-IBD patients	Karolinska University Hospital or Sachs’ Children and Youth Hospital	N/A
Intestinal specimens from adult IBD and non-IBD patients	Karolinska University Hospital	N/A
Peripheral blood from pediatric IBD patients	Karolinska University Hospital	N/A

**Deposited data**		

Count matrix from cell ranger	This paper	GEO Accession Number: GSE169136
R scripts	This paper	https://github.com/ramvinay/Kokkinou_pIBD https://github.com/jtheorell/Kokkinou_gut_neighbors/

**Software and algorithms**		

R v.4.1.0	R Core Team	https://cran.r-project.org/
Cell Ranger v.2.1.1	10x Genomics	https://support.10xgenomics.com/single-cell-gene-expression/software
Seurat v.4.1.1	Butler et al., 2019^60^	https://github.com/satijalab/seurat/
Harmony v.0.1	Korsunsky et al., 2019^61^	https://github.com/immunogenomics/harmony
sva v.3.32.1	Leek et al., 2022^62^	https://bioconductor.org/packages/release/bioc/html/sva.html
Slingshot v.2.2.1	Street et al., 2018^63^	https://bioconductor.org/packages/release/bioc/html/slingshot.html
WGCNA v.1.70.3	Langfelder et al., 2008^23^	https://horvath.genetics.ucla.edu/html/CoexpressionNetwork/Rpackages/WGCNA/
SingleCellExperiment	Amezquita et al., 2020^64^	https://www.bioconductor.org/packages/release/bioc/html/SingleCellExperiment.html
DASeq v1.0.0	Zhao et al., 2021^38^	https://github.com/KlugerLab/DAseq
DepecheR v.1.14.0	Theorell et al., 2019^65^	https://www.bioconductor.org/packages/release/bioc/html/DepecheR.html

**Other**		

LIVE/DEAD™ Fixable Near-IR Dead Cell Stain Kit	Invitrogen	Cat#L34975
LIVE/DEAD™ Fixable Aqua Dead Cell Stain Kit	Invitrogen	Cat#L34957
LIVE/DEAD™ Fixable Green Dead Cell Stain Kit	Invitrogen	Cat#L34970
Collagenase II	Sigma-Aldrich	Cat#C6885
DNAse	Sigma-Aldrich	Cat#10104159001
IMDM	Gibco	Cat#12440-053
Foxp3/Transcription Factor Staining Buffer Set	eBioscience	Cat#00-5523-00
Chromium™ Single Cell 3' Library & Gel Bead Kit v2, 16 rxns	10x Genomics	Cat#PN-120237

### Resource availability

#### Lead contact

Further information and requests for resources should be directed to and will be fulfilled by the lead contact, Jenny Mjösberg (jenny.mjosberg@ki.se).

#### Materials availability

The study did not generate new reagents.

### Experimental model and subject details

#### Human patients and study design

Pediatric patients at the Karolinska University Hospital or Sachs’ Children and Youth Hospital were included. All patients were treatment-naive and included upon diagnostic colonoscopy. IBD colitis diagnosis was endoscopically and histologically verified.

For flow cytometry analysis, 16 biopsies were obtained from two areas in the colon of pediatric patients with IBD (n = 19): the endoscopically most affected area (inflamed) and the endoscopically least-affected area (non-inflamed) (Table S1). Some patients had pancolitis or restricted ileal or upper GI-tract involvement, and thus, samples were taken only from the endoscopically inflamed or non-inflamed colonic mucosa, respectively. Non-IBD controls (n = 10), displayed unspecific gastrointestinal symptoms and colonoscopy was performed to rule out IBD diagnosis. All non-IBD controls had endoscopically and histologically normal gut mucosal findings with no signs of active or chronic inflammation.

For the scRNA-seq, treatment-naïve pediatric patients with CD-colitis were included (n = 6). Biopsies were sampled from endoscopically most inflamed and adjacent least inflamed areas of the colon and histologically evaluated (Table S3).

Colonic intestinal specimens from adult patients (Table S5), include non-IBD controls (non-affected intestine from colorectal cancer patients) (n = 8) as well as IBD patients that underwent surgery (n = 8, 2 UC, 6 CD).

#### Ethical approval

This study was approved by the Swedish Ethical Review Authority (approval numbers 2010/32-31/4, 2018/323-31/1 and 2020-00507). Written informed consents were obtained from all patients and their caregivers.

#### Characterization and inflammation activity scoring of patients with pIBD

Disease activity was evaluated with endoscopic and histopathological scorings. To evaluate the disease activity endoscopically, we used the endoscopic part of SWIBREG (Swedish Inflammatory Bowel Disease Register) scoring system (Table S2).^20^ For pediatric CD patients, SES-CD endoscopic score and Paris phenotypic classification were additionally assessed to characterize the patient group. For histopathological scoring, we used a modification of colonic global histologic disease activity score of diagnostic samples collected in close proximity to the research biopsies.^21^ The evaluation was based on the written statement of an IBD-experienced pathologist. Established IBD biomarkers (shown in Tables S1 and S5), were taken as routine diagnostic examination: blood 0-36 (median 3) and fecal samples 0-60 (median 18) days prior to biopsy sampling and are listed for characterization of the patient groups.

### Method details

#### Cell isolation from intestinal samples and blood

Cell isolation from colonic biopsies was performed as described in Mazzurana *et al* 2019, where the detailed protocols are provided.^66^ Briefly, colonic biopsies were collected in wash buffer containing HBSS supplemented with antibiotics. For subsequent enzymatic digestion, wash buffer was removed and replaced with 1mL IMDM (supplemented with antibiotics), collagenase II 0.25 mg/mL; Sigma) and DNase (0.25 mg/mL; Roche). Magnetic stirring was performed at 37°C, 450rpm.

For 3 pIBD patients (Table S1), peripheral blood mononuclear cells (PBMC) were isolated as previously described^29^^,^^66^ using Lymphoprep™ (Stemcell Technologies) with gradient density centrifugation at 2000 rpm for 20 min, room temperature (RT). Cells were collected, washed, counted and subjected to cell staining.

#### Flow cytometry and cell sorting

For flow cytometry, colon cells or PBMC were stained with dead cell marker (DCM) and antibodies targeting surface antigens for 20-30 min at RT, followed by washing with FACS buffer (PBS containing 1% FCS). The cells were fixed with either 2% PFA for 10 min at RT, or in case of intracellular staining, with Foxp3 Fix/Perm solution kit (eBioscience) for 30 minutes at RT. For the latter, cells were subsequently washed with permeabilization buffer (eBioscience) and stained with antibodies targeting intracellular antigens for 30 min at RT. Samples were immediately acquired on a BD LSR Fortessa or BD Symphony A5 flow cytometer. Analysis of flow cytometry data was performed using FlowJo versions 9.9.6, 10.4.2 and 10.6.2 (TreeStar). A detailed list of the antibodies are shown in the key resources table.

For cell sorting, colonic ILCs, T cells and NK cells were surface-stained with lineage specific antibodies (all antibodies are indicated in the key resources table). Cells were sort-purified in cold RMPI using the BD FACSAria Fusion™. Gating strategy for sorted ILCs, T cells and NK cells is shown in Figure S1A. After sorting cells were immediately subjected to RNA library preparation.

#### 10x genomics single-cell RNA sequencing

Chromium Single-Cell v2 3ʹ Reagents Kit (10x Genomics) was used to generate single-cell RNA Sequencing libraries. In brief, cell suspension was spun down and resuspended in 17.4μl PBS with 0.04% BSA at a final concentration of 1000 cells/μl (target recovery of 10 000 cells). Accordingly, Master Mix was prepared at the adjusted volume of 16.4μl Nuclease-Free water. Cells were loaded on the Chromium Controller Single Cell A Chip to generate Gel Bead Emulsions (GEMs). GEMs were proceeded to GEM-RT incubation, cDNA amplification and library construction following the Chromium™ Single Cell 3′ library Kit v2 User Guide (10x Genomics). Quality controls for cDNA amplification and final barcoded libraries were performed using High Sensitivity DNA Assays from 2100 Agilent Bioanalyzer to assess the quantity and fragment size. Sequencing libraries were loaded at 2.1pM loading concentration on an HiSeq4000 with custom sequencing metrics (single-indexed sequencing run, 28/8/0/98 cycles for R1/i7/i5/R2) (Illumina, San Diego, CA).

### Quantification and statistical analysis

#### Single-cell RNA sequencing data analysis

10x Cell Ranger toolkit (v2.1.1) was used for filtering and aligning reads onto human genome hg19 and counting barcodes and unique molecular identifiers (UMI). Seurat (v4.1.1)^60^ was used for downstream 10x scRNA-seq data analysis. Briefly, cells with a percentage of mitochondria genes > 5% were considered as dead cells and were removed. Cells with < 200 genes and > 2500 detected genes were excluded. Genes detected in < 3 cells were excluded. Raw UMI counts were scaled and normalized through log transformation. We used Harmony for performing the integration of samples.^61^ The top 4000 variable genes were selected for PCA and the first 40 dimensions were selected for UMAP and clustering analysis with resolutions 0.6. Seurat function FindAllMarkers (logFC threshold = 0.25) were used to find cluster- and sample-specific marker genes. Further, UMAP clusters were annotated by well-known marker genes and marker genes were detected by the *FindAllMarke*rs function. The functions *FeaturePlot*, *VlnPlot* and *DotPlot* were used for feature plots, violins plots, heatmaps and dot plots, respectively, with default parameters. For subcluster analysis, we used the ComBat function from sva (v3.32.1)^62^ R package to remove known batches (donors). Wilcoxon test was used for the DE analysis. Trajectory analysis on ILCs was performed using the Slingshot version 2.2.1^63^ R package with Seurat object. We used UMAP coordinates and ILC subclusters 4 and 1 as the starting point (root).

#### Weighted gene co-expression network analysis (WGCNA)

We used the weighted co-expression network analysis (WGCNA)^23^ algorithm to identify gene co-expression networks. Specifically, 4000 variable genes were used for calculation of gene-to-gene Pearson correlations. The adjacency matrix was directly used for creation of the topological overlap matrix (TOM). Gene module identification was done with agglomerative hierarchical clustering on the TOM using Ward’s squared linkage (Ward.D2) with a fixed number of 50 modules. Gene counts belonging to the same module were normalized between 0–1 individually and then averaged per cell for calculation of each gene module expression pattern. Modules were prioritized by fitting a general linear model (GLM) using each gene module expression pattern as the effect and the sample metadata (Louvain cluster, Donor) as the explaining variables. Each module and metadata parameter was tested independently. The output of the GLM fit results in the residual metric of “deviance” (*dev*_*model*_) after fitting the model and “null deviance” (*dev*_*null*_) when considering a NULL model. The percent difference in these two deviances (Δ*dev*) was used as a measure of how much a particular metadata factor explains the expression pattern of a gene module:

$$\Delta dev=\left({dev}_{null}-{dev}_{model}\right)/{dev}_{null}$$

This way, modules with higherΔ*dev*were prioritized for further exploration.

#### Euclidean neighbor-based smoothing of histology inflammation score

Since samples that are endoscopically scored as non-inflamed or inflamed are composed of samples with variable histological scores we needed a method to better define “inflammatory” and “non-inflammatory” cells to potentially discover genes involved in pIBD inflammation. This method builds on the concept of Euclidean neighbor-based smoothing, which has previously been used both in cytometry and scRNAseq analysis.^37^^,^^38^ Its core assumption is that Euclidean neighbors in the transcriptomic space have similar functions.

The processing of the data was done using the neighSmooth function in the BioConductor package DepecheR, in a similar fashion as in Hart *et al*^37^. To even out differences in cell numbers between donors and conditions, the samples were divided into least inflamed (histo score 0-4), intermediate (histo score 7) and most inflamed (histo score 9-12). Then, random subsets of cells were chosen, so that each sample within these overarching inflammation groups had the same number of cells, and simultaneously that the least, intermediate, and most inflamed sample groups had the same number of total cells. In the end, a total number of 22065 neighbor cells were identified, ranging between 1461 and 3681 cells for each sample. After this, the 100 closest Euclidean neighbors were identified from this neighbor cell subset for all cells in the dataset, and each cell was given the average histo score for these 100 nearest neighbors. Each cell was further assessed for the number of samples represented among its 100 nearest neighbors. An arbitrary cutoff of five samples was used to filter out cell populations strongly overrepresented in outlier samples. For this more representative group of cells, aided by a histogram, cutoff values of ≤ 4 for least inflamed cells and ≥ 9 for most inflamed cells were introduced.

To confirm the results of the nearest neighbor-smoothing analysis of the histological scores, DAseq was used.^38^ As this is a discriminant analysis tool, a few adaptions of the data was necessary. First, all samples with score 7 were excluded. Second, if both samples from one individual had low (<4) or high (≥9) histological scores, the least extreme sample was removed. Subsequently, all remaining cells in the low or the high inflammation group were given these discriminant labels. This was followed by the standard DASeq pipeline. As a prediction threshold, the value of 0.5 was used.

#### Statistical analyses

Statistical analysis and graphing of the flow cytometry data was performed using Prism 9 software (GraphPad Software, San Diego, US). The sample size for each experiment, the replicate number as well as the precision measures are included in the figure legends.

## Data Availability

• ScRNA-seq data (count matrix) are available at GEO under the accession number GSE169136. • All codes (R scripts) have been deposited at: https://github.com/ramvinay/Kokkinou_pIBD and https://github.com/jtheorell/Kokkinou_gut_neighbors/ • Software and packages used for this analysis are listed in the key resources table. • Any additional information required to reanalyze the data reported in this paper is available from the lead contact upon request.

## Supplemental information

Document S1. Figures S1–S5 and Tables S1–S3 and S5

Table S4. Genes upregulated in the most and least inflamed clusters, related to Figure 5

Document S2. Article plus supplemental information
