# Supplementary material for: The single-cell transcriptional landscape of innate and adaptive lymphocytes in pediatric-onset colitis
Source: Cell Rep Med. 2023 May 8;4(5):101038. doi: 10.1016/j.xcrm.2023.101038 (PMC10213874; doi:10.1016/j.xcrm.2023.101038)
Supplement: Document S1. Figures S1–S5 and Tables S1–S3 and S5 [file mmc1.pdf]

Cell Reports Medicine, Volume *4*

Supplemental information

The single-cell transcriptional landscape of innate and adaptive lymphocytes in pediatric-onset colitis

Efthymia Kokkinou, Tea Soini, Ram Vinay Pandey, Aline van Acker, Jakob Theorell, Paulo Czarnewski, Egle Kvedaraite, Niels Vandamme, Magda Lourda, Chiara Sorini, Whitney Weigel, Anna Carrasco, Christopher Andrew Tibbitt, Heinrich Schlums, Ulrik Lindforss, Caroline Nordenvall, Malin Ljunggren, Maja Ideström, Mattias Svensson, Jan-Inge Henter, Eduardo J. Villablanca, Yenan T. Bryceson, Helena Rolandsdotter, and Jenny Mjösberg

Supplemental information

The single-cell transcriptional landscape of innate and adaptive lymphocytes in pediatric-onset colitis

Efthymia Kokkinou, Tea Soini, Ram Vinay Pandey, Aline van Acker, Jakob Theorell, Paulo Czarnewski, Egle Kvedaraite, Niels Vandamme, Magda Lourda, Chiara Sorini, Whitney Weigel, Anna Carrasco, Christopher Andrew Tibbitt, Heinrich Schlums, Ulrik Lindforss, Caroline Nordenvall, Malin Ljunggren, Maja Ideström, Mattias Svensson, Jan-Inge Henter, Eduardo J. Villablanca, Yenan T. Bryceson, Helena Rolandsdotter, Jenny Mjösberg

Figure S1

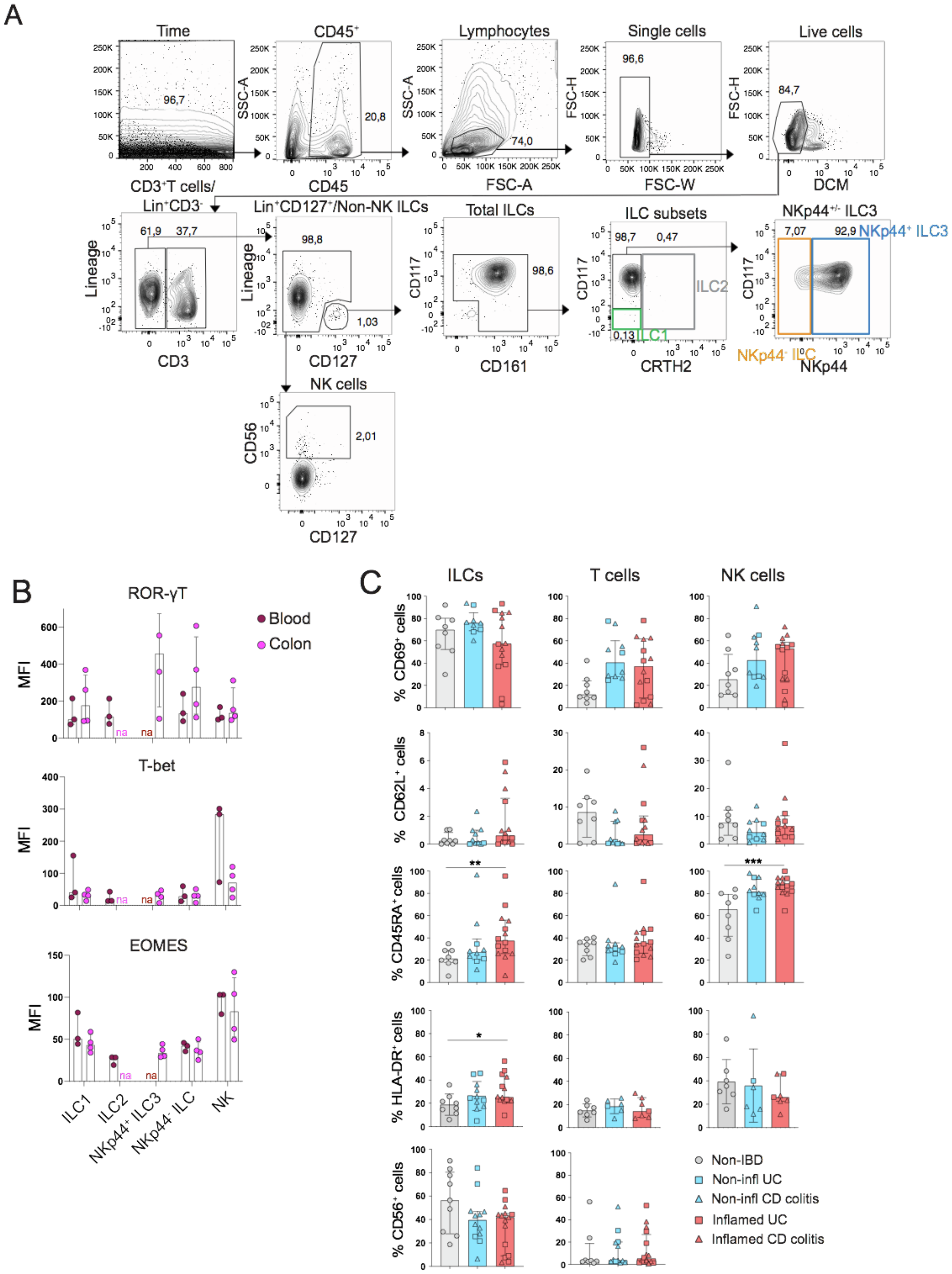

A
Time
CD45+
Lymphocytes
Single cells
Live cells
CD3+T cells/
Lin+CD3-
Lin+CD127+/Non-NK ILCs
Total ILCs
ILC subsets
NKp44+/- ILC3
NKp44+ ILC3
NKp44- ILC
ILC2
ILC1
NK cells
SSC-A
FSC-H
CD45
FSC-A
FSC-W
DCM
Lineage
CD3
CD127
CD117
CD161
CRTH2
NKp44
CD56
96,7
20,8
74,0
96,6
84,7
61,9
37,7
98,8
1,03
98,6
98,7
0,47
0,13
7,07
92,9
2,01
B
ROR-γT
T-bet
EOMES
MFI
Blood
Colon
na
ILC1
ILC2
NKp44+ ILC3
NKp44- ILC
NK
C
ILCs
T cells
NK cells
% CD69+ cells
% CD62L+ cells
% CD45RA+ cells
% HLA-DR+ cells
% CD56+ cells
Non-IBD
Non-infl UC
Non-infl CD colitis
Inflamed UC
Inflamed CD colitis

Figure S1. Dysregulation of colonic ILC composition in pIBD colitis. Related to Figure 1. (A) Gating strategy for the identification of T cells ($CD3^{+}$ Lin^{-}), ILCs ($CD3^{-}Lin^{-}CD127^{+}$ $CD161^{+}$) and NK cells ($CD3^{-}Lin^{+}$ $CD127^{-/dim}$ $CD56^{+}$) by flow cytometry in pediatric (p)IBD and non-IBD colon samples. **(B)** Bar graphs showing the geometric MFI expression of ROR-γT, T-bet and EOMES protein in blood (n = 3) and colon samples (n = 4) of pediatric non-IBD patients. **(C)** Bar graphs showing the frequencies of $CD69^{+}$, $CD62L^{+}$, $CD45RA^{+}$, HLA-DR^{+} and $CD56^{+}$ cells out of ILCs, T cells and NK cells in non-IBD (n = 8-9), endoscopically non-inflamed (n = 6-12) and endoscopically inflamed (n = 6-14) pIBD colon samples (mean with SD). Statistical differences were detected with the Mann-Whitney test. Bar graphs are shown as Mean with SD. In (B) and (C) cells from each donor were analyzed in independent experiments.

Figure S2

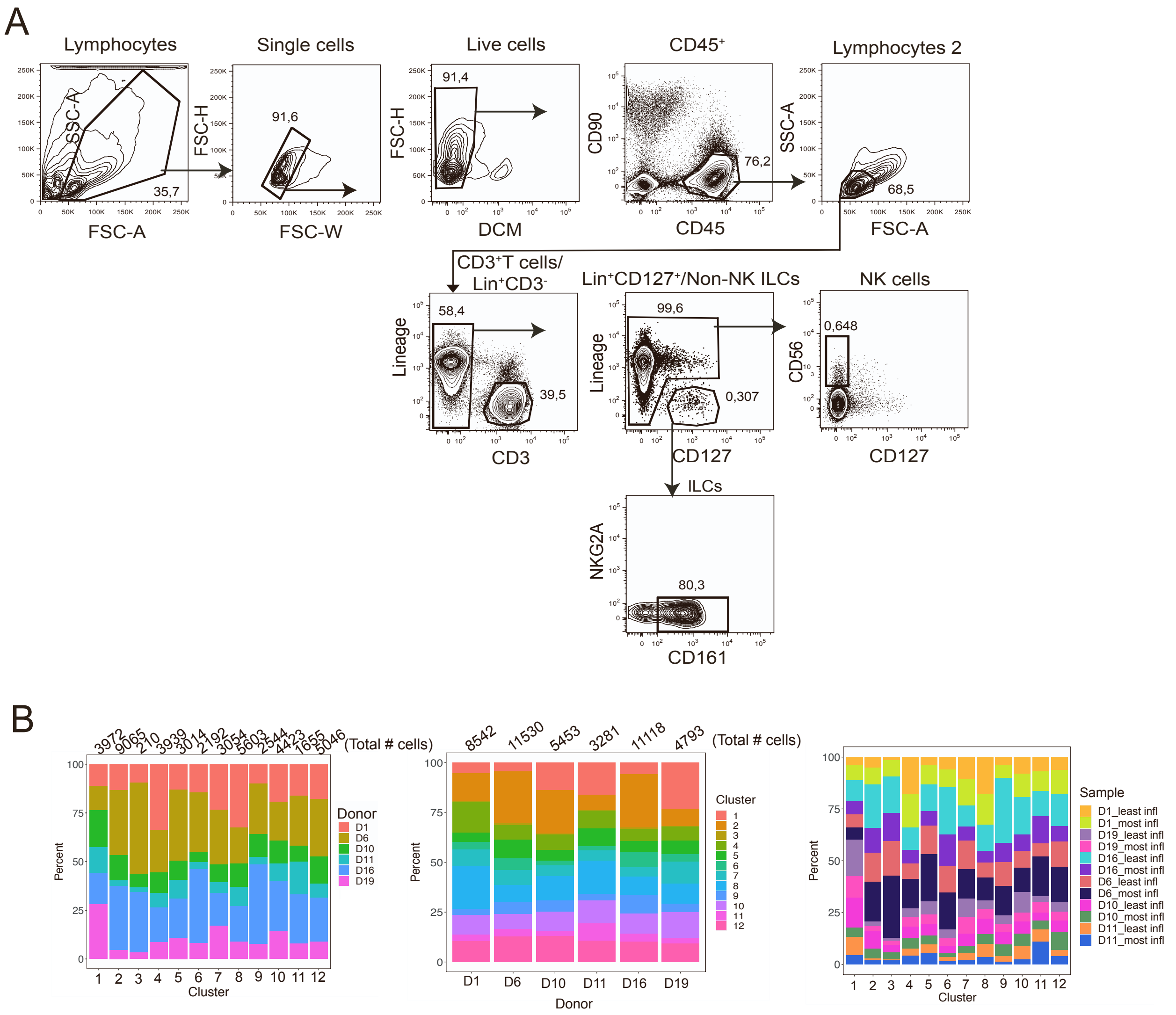

Figure S2. Single-cell transcriptional profiling of colonic ILCs, NK- and T cells in pIBD colitis. Related to Figure 2. (A) FACS-sorting gating strategy for T cells (CD3$^+$ Lin$^-$), ILCs (CD3$^-$Lin$^-$CD127$^+$ CD161$^+$) and NK cells (CD3$^-$Lin$^+$ CD127$^{-/dim}$ CD56$^+$) for scRNA-seq. **(B)** Stacked bar plot showing the contribution of each donor (n = 6) to the clusters shown in Figure 2B (left and middle). Stacked bar plot showing the contribution of each colon biopsy sample (n = 12) to the clusters shown in Figure 2B (right). Cells from each donor were analyzed in independent experiments.

Figure S3

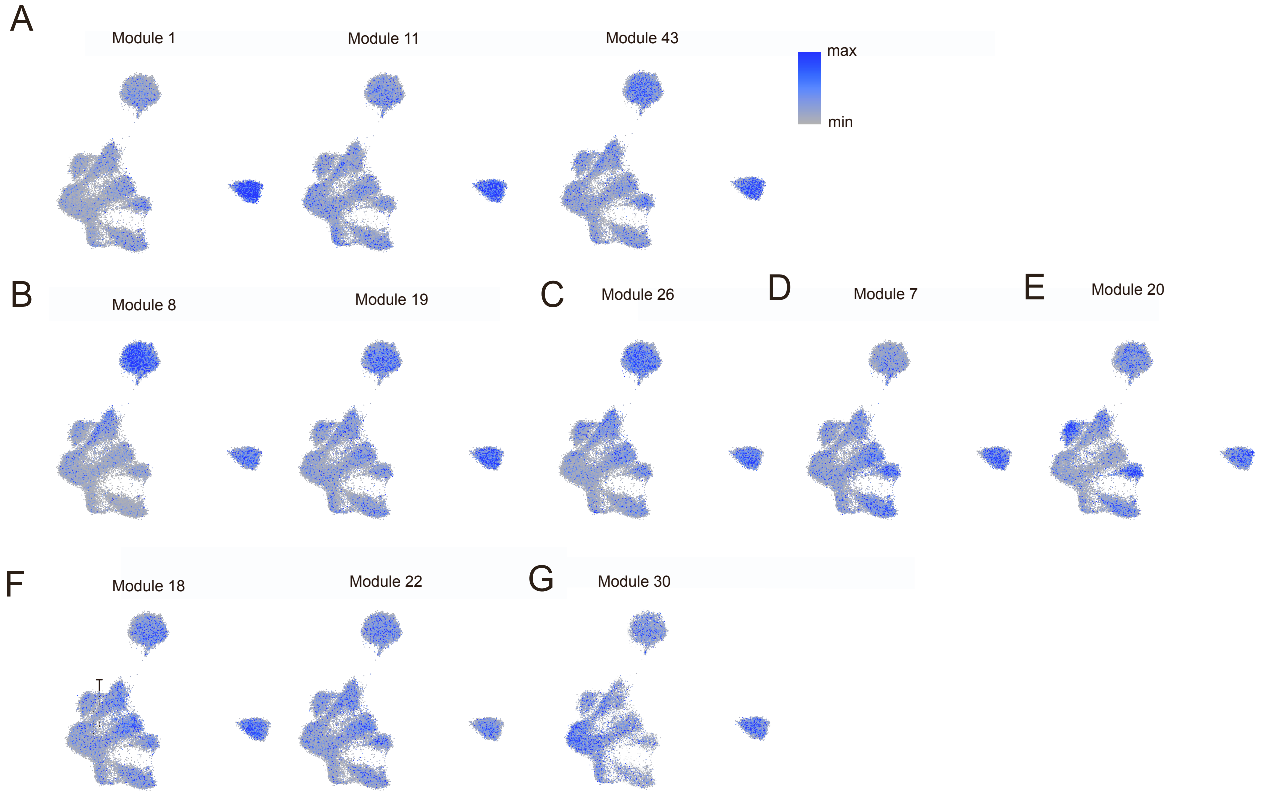

Figure S3. Gene module analysis of ILCs, NK cells and T cells. Related to Figure 3.

(A-G) UMAP illustration of the expression of selected gene modules shown in Figure 3. High expression of a gene module is shown as blue (max) and low expression of a gene module is shown as grey (min).

Figure S4

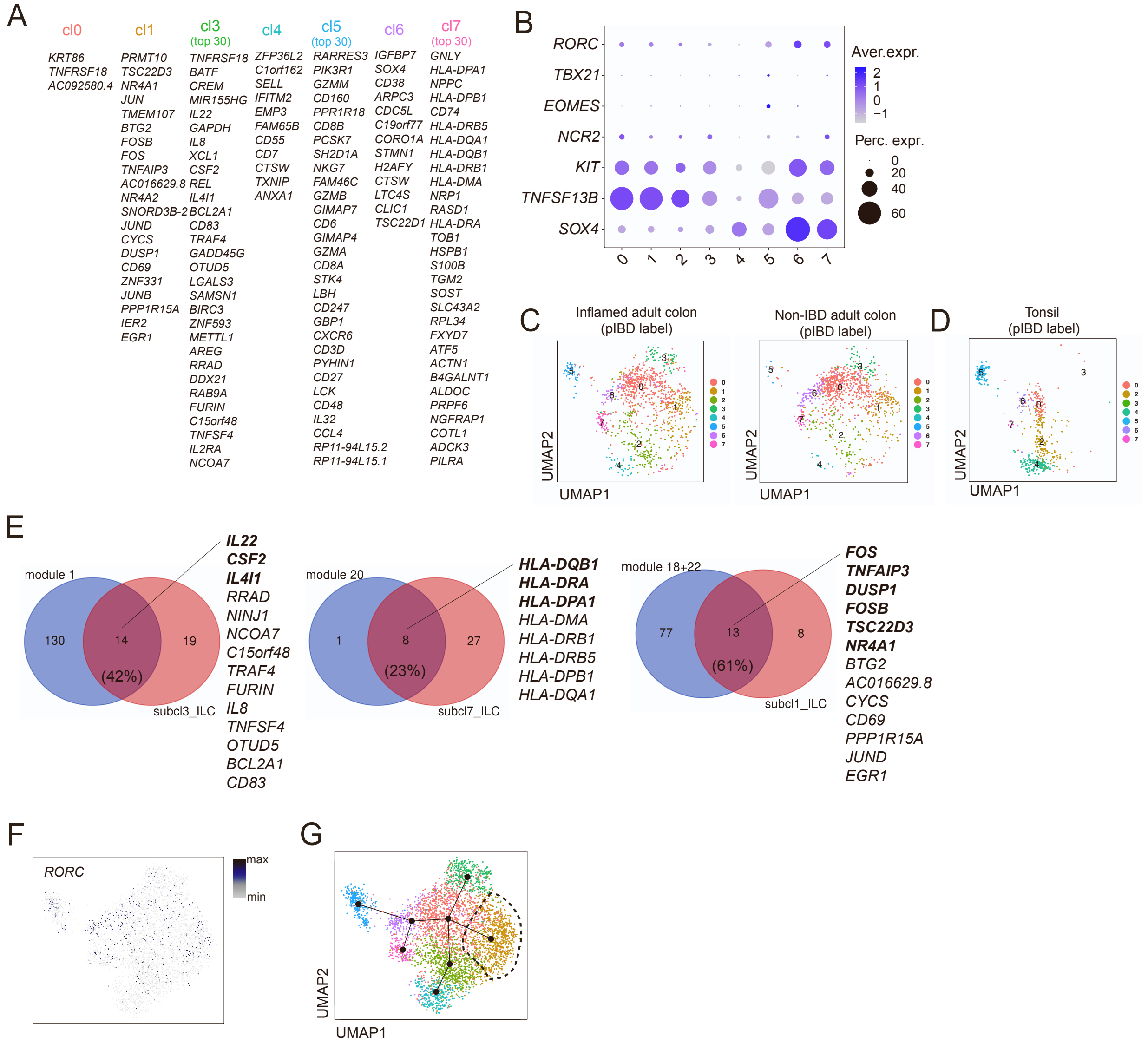

Figure S4. Subcluster analysis of colonic ILCs. Related to Figure 4. (A) Table showing the DEGs (log2fc > 0.10 and adj. p-value < 0.05) for each ILC subcluster (ordered from lowest to highest). For clusters 3, 5 and 7 the top 30 genes are shown. **(B)** Dotplot showing the expression of selected genes for the annotation of the ILC subclusters. **(C)** Inflamed and non-IBD ILCs from adult patients, projected onto the pIBD ILC UMAP shown in Figure 4A. **(D)** Tonsil ILCs from adult patients, projected onto the pIBD ILC UMAP shown in Figure 4A. **(E)** Venn diagrams showing the overlap between DEGs in ILC subcluster 3 (subcl3_ILC) and module 1 described in Figure 3C, ILC subcluster 7 (subcl7_ILC) and module 20 described in Figure 3G, ILC subcluster 1 (subcl1_ILC) and modules 18+22 described in Figure 3H. The overlapping genes in the intersection of the circles are indicated. In bold are the genes shown in Figure 4. The gene frequency is derived from the number of genes the ILC subclusters share with the modules (number in the intersection) divided by the total DEGs (log2fc > 0.10 and adj. p-value < 0.05) in each ILC subcluster. **(F)** UMAP

illustration of *RORC* expression in ILC subclusters. **(G)** Developmental trajectories of ILC subclusters performed with Slingshot. Subcluster 1 (dotted circle) was used as the root.

Figure S5

A

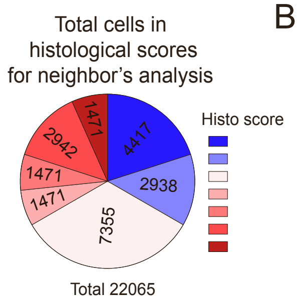

B

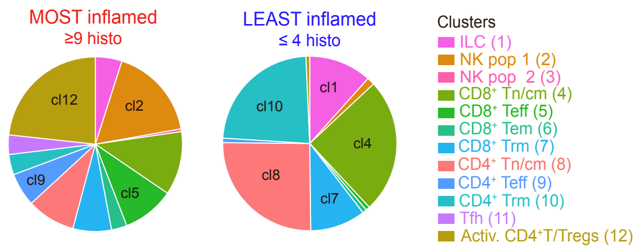

C

Sample (histo score)	1 (1)	2 (3)	3 (3)	4 (7)	5 (7)	6 (10)	7 (1)	8 (10)	9 (11)	10 (12)	11 (1)	12 (11)
>9 histo score												
cl1	0	0	0	0	0	0	0	0	64	26,8	2,9	5,9
cl2	0,4	0	3,4	0,4	0	0,4	0	0,4	25	41	1,7	25,6
cl3	0	0	0	0	0	0	0	0	14	85	0	0
cl4	0	0	0	0	0	0	0	0	35	56	2,5	5,6
cl5	0	0	0	0	0	0	0	0	55	42,7	0,7	1,5
cl6	0	0	0	0	0	0	0	0	46	54	0	0
cl7	0	0	0	0	0	0	0	0	52	43,2	1	3
cl8	0	0	0	0	0	0	0	1	54	36,8	2,4	5,7
cl9	0	0	0	0	0	0	0	0	41,6	53,5	2,3	2,3
cl10	0	0	0	0	0	0	0	0	45	54,9	0	0
cl11	0	0	0	0	0	0	0	0	305	42,8	2	24,4
cl12	0	0	0	0	0	0	0	0	67	201	6	42
<4 histo score												
cl1	4,8	8,6	4,5	2,2	1,7	1	25,3	5,9	0,3	0	41,1	4,1
cl2	37,1	30	1,4	1,4	0	0	21,4	0	0	0	8,5	0
cl3	0	0	0	0	0	0	0	0	0	0	0	0
cl4	40	37,2	5,5	7	1,2	3,8	1,8	1,2	0,008	0	1,1	0,6
cl5	52	19,4	0	0	0	0	25	2,7	0	0	0	0
cl6	35,8	28,2	5,1	0	0	2,5	2,5	5,1	2,5	0	15,3	2,5
cl7	31,4	43,3	3,7	1,5	1,3	0,7	5,1	2,5	0	0	9,2	0,7
cl8	47	26,1	8,7	3	1,1	2,1	1,9	0,9	0,1	0		0,3
cl9	9,7	17	0	0	0	0	41,7	2,4	0	0	39	0
cl10	15,5	17,5	18,5	4,9	0,9	0,8	17,6	3,4	0,4	0,6	17,3	2
cl11	0	0	0	0	0	0	0	0	0	0	0	0
cl12	31,4	48,5	8,5	0	0	0	2,8	2,8	0	0	5,7	0

D

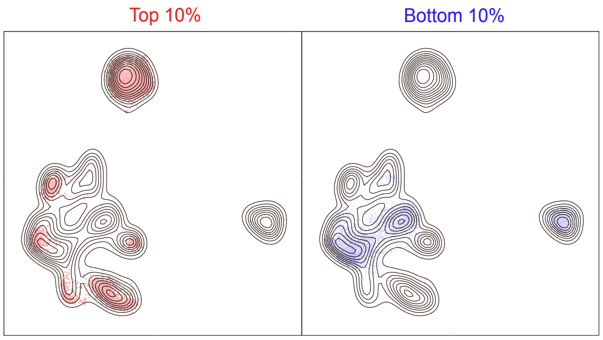

E

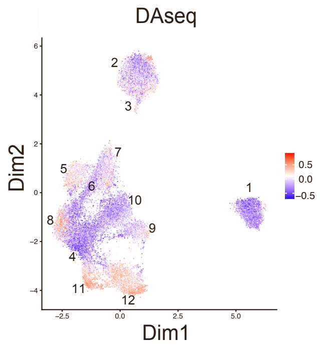

Figure S5. Distribution of most- and least inflamed cells across cell clusters. Related to Figure 5. (A) Pie chart showing the total number of cells in the histological scores post-sampling of the nearest-neighbor analysis shown in Figure 5. **(B)** Pie chart summaries showing the enrichment of most and least inflamed cells in the main UMAP clusters. **(C)** Table showing the percentage (%) contribution of each sample to cl1-12 of the >9 and <4 histo score UMAPs of Figure 5F. **(D)** Visualization of cells from the nearest-neighbor smoothing on the main UMAP with cells derived from top 10% (most inflamed) and bottom 10% (least inflamed) groups after Harmony batch correction. **(E)** Prediction UMAP plot from the DAseq analysis. Blue indicates cells with low histological score and red with high histological score.

Table S1. Patient characteristics of pediatric IBD flow cytometry cohort. Related to Figure 1.

Pediatric IBD patients

N (total)	19
Diagnosis (% UC)	37
Diagnosis (% CD)	63
Age (years) mean; IQR	14; 6
Sex (% Female)	58
P-CRP (mg/L) range; median	1-99; 1
ESR (mm/h) range; median	3-84; 26
F-calprotectin (mg/kg) range; median	519-5000; 1820
S-albumin (mg/L) range; median	22-44; 35
B-Hb (mg/L) range; median	52-149; 114
n inflamed samples	15
Inflamed sampling location colon (N), descendens/ascendens/transversum/multiple locations/NA	7/6/1/1/4
Inflamed samples: Endoscopic score, range; median	1-3; 2
Inflamed samples: Histologic score, range; median	4-13; 9
n non-inflamed samples	12
Non-inflamed sampling colon location (N), descendens/ascendens/transverum/multiple locations/NA	3/5/2/2/6
Non-inflamed samples: Endoscopic score, range; median	0-0; 0
Non-inflamed samples: Histologic score, range; median	1-5; 1

Pediatric non-IBD controls

N (total)	10	
Age (years) mean; IQR	13; 3	*p = 0.4 (compared to IBD group)
Sex (% Female)	70	**p = 0.5 (compared to IBD group)
P-CRP (mg/L) range; median	1-1; 1	
ESR (mm/h) range; median	2-20; 7	
F-calprotectin (mg/kg) range; median	9-1922; 112	
S-albumin (mg/L) range; median	39-48 (43)	
B-Hb (mg/L) range; median	120-150; 134	
Sampling location colon (N), descendens/ascendens/tranverse/multiple locations/NA	1/6/1/2/0	
Endoscopic score, range; median	0-0; 0	
Histologic score, range; median	1-1; 1	

*Unpaired t-test
**Chi-square test

Table S2. SWIBREG endoscopic scoring for UC and CD. Related to Figure 1.

**SWIBREG scoring for UC (=Mayo score)**			
0 = remission			
1= mild disease	Erythema	Decreased vascular pattern	Mild friability
2 = moderate disease	Marked erythema	Lack of vascular pattern	Friability and erosions
3 =severe disease	Spontaneous bleeding	Ulceration	
**SWIBREG scoring for CD**			
0 = remission			
1= mild disease	Small aphthous wounds on an otherwise normal mucosa		
2 = moderate disease	Erythema, edema	Lack of vascular pattern	Friability, no ulceration
3 = severe disease	Marked erythema, edema	Lack of vascular pattern	Ulceration

Table S3. Patient characteristics of pediatric IBD scRNA-seq cohort. Related to Figure 2.

Pediatric IBD patients	
N (total)	6
Diagnosis (% CD)	100
Age (years) mean; IQR	14; 3
Sex (% female)	33
SES-CD, range; median	8-28; 18
P-CRP (mg/L) range; median	5-99; 9
ESR (mm/h) range; median	7-74; 29
F-calprotectin (mg/kg) range; median	615-2750; 1435
S-albumin (mg/L) range; median	31-41; 37
B-Hb (mg/L) range; median	83-153; 130
n most inflamed samples	6
Most inflamed sampling location colon (N), descendens/ascendens/transversum	2/4/0
Most inflamed samples: Endoscopic score, range; median	2-3; 2
Most inflamed samples: Histologic score, range; median	3-11; 10
n least inflamed samples	6
Least inflamed samples: Endoscopic score, range; median	0-2; 0
Least inflamed samples: Histologic score, range; median	1-11; 2
Least inflamed sampling location colon (N), descendens/ascendens/transversum	2/2/2

Table S5. Patient characteristics of adult IBD flow cytometry cohort. Related to Figure 5I.

Adult IBD patients

N (total)	8	
Diagnosis UC (%)	25%	
Diagnosis CD (%)	75%	
Age (years) mean; IQR	39; 24	
Sex (% female)	50	
Sampling location colon (N), rectum/descendens/ascendens/transversum/NA	3/4/0/0/1	

Adult non-IBD controls

N (total)	8	
Diagnosis (% colorectal cancer)	100	
Age (years) mean; IQR	79; 10	**p* = <0.05 (compared to IBD group)
Sex (% female)	37	***p* = 0.6 (compared to IBD group)
Sampling location colon (N), rectum/descendens/ascendens/transversum/NA	0/4/1/3/0	

*Unpaired t-test

**Chi-square test
