# Supplementary material for: The single-cell transcriptional landscape of innate and adaptive lymphocytes in pediatric-onset colitis
Source: Cell Rep Med. 2023 May 8;4(5):101038. doi: 10.1016/j.xcrm.2023.101038 (PMC10213874; doi:10.1016/j.xcrm.2023.101038)
Supplement: Document S2. Article plus supplemental information [file mmc3.pdf]

Cell Reports Medicine

Article

# The single-cell transcriptional landscape of innate and adaptive lymphocytes in pediatric-onset colitis

## Graphical abstract

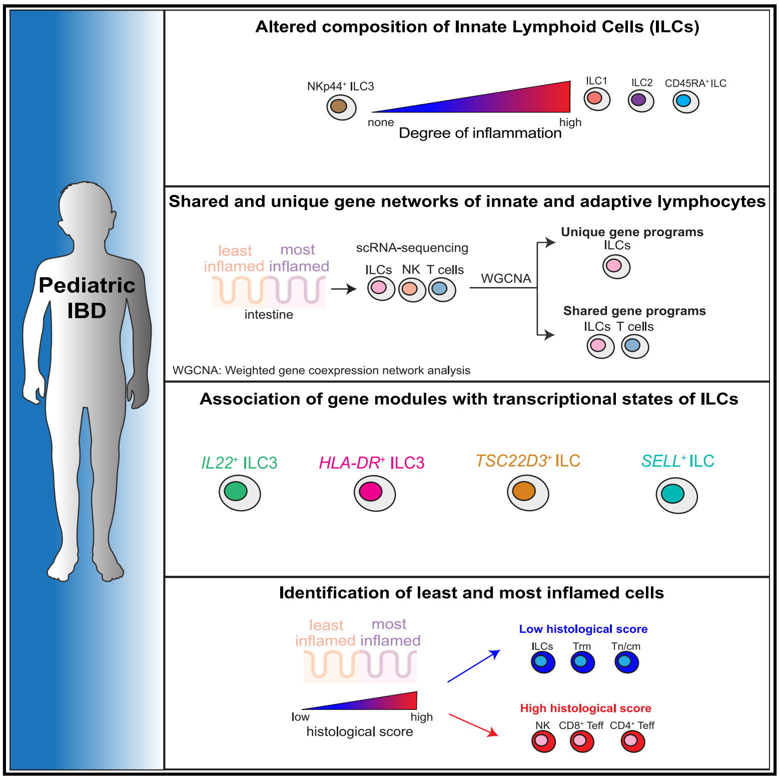


## Highlights

- Innate lymphoid cell (ILC) balance is altered in pediatric IBD (pIBD)
- Colonic ILCs and T cells have shared and unique transcriptional programs
- PIBD inflammation is characterized by transcriptional changes in both ILCs and T cells


## Authors

Efthymia Kokkinou, Tea Soini, Ram Vinay Pandey, ..., Yenan T. Bryceson, Helena Rolandsdotter, Jenny Mjösberg

## Correspondence

jenny.mjosberg@ki.se



## In brief

The role of innate lymphoid cells (ILCs) in pediatric IBD (pIBD) remains understudied. Here, Kokkinou et al. show that while some transcriptional features of colonic ILCs in pIBD are unique, some are shared with T cells. pIBD inflammation leads to transcriptional and cellular changes in both ILCs and T cells.



Kokkinou et al., 2023, Cell Reports Medicine *4*, 101038
May 16, 2023 © 2023 The Author(s).
https://doi.org/10.1016/j.xcrm.2023.101038


CellPress

Cell Reports Medicine

CellPress
OPEN ACCESS

Article

# The single-cell transcriptional landscape of innate and adaptive lymphocytes in pediatric-onset colitis

Efthymia Kokkinou,[1] Tea Soini,[1,17] Ram Vinay Pandey,[2,17] Aline van Acker,[1,15,17] Jakob Theorell,[1,3] Paulo Czarnewski,[4] Egle Kvedaraite,[1,5,6] Niels Vandamme,[7,8] Magda Lourda,[1,5] Chiara Sorini,[9] Whitney Weigel,[1] Anna Carrasco,[1] Christopher Andrew Tibbitt,[1] Heinrich Schlums,[2] Ulrik Lindforss,[10] Caroline Nordenvall,[10] Malin Ljunggren,[10] Maja Ideström,[5,11] Mattias Svensson,[1] Jan-Inge Henter,[5,12] Eduardo J. Villablanca,[9] Yenan T. Bryceson,[2] Helena Rolandsdotter,[13,14,16] and Jenny Mjösberg[1,16,18,*]

[1]Center for Infectious Medicine, Department of Medicine Huddinge, Karolinska Institutet, Karolinska University Hospital Huddinge, Stockholm, Sweden
[2]Center for Hematology and Regenerative Medicine, Department of Medicine, Karolinska Institutet, Karolinska University Hospital Huddinge, Stockholm, Sweden
[3]Psychiatry Southwest, Health Care Services Stockholm County, Huddinge, Sweden
[4]Science for Life Laboratory, Department of Biochemistry and Biophysics, National Bioinformatics Infrastructure Sweden, Stockholm University, Solna, Sweden
[5]Childhood Cancer Research Unit, Department of Women's and Children's Health, Karolinska Institutet, Karolinska University Hospital, Stockholm, Sweden
[6]Department of Clinical Pathology and Cancer Diagnostics, Karolinska University Hospital, Stockholm, Sweden
[7]Data Mining and Modeling for Biomedicine, VIB Center for Inflammation Research, Ghent, Belgium
[8]Department of Applied Mathematics, Computer Science and Statistics, Ghent University, Ghent, Belgium
[9]Immunology and Allergy Unit, Department of Medicine, Solna, Karolinska Institutet and University Hospital, Stockholm, Sweden
[10]Department of Molecular Medicine and Surgery, Karolinska Institutet and Center for Digestive Diseases, Karolinska University Hospital, Stockholm, Sweden
[11]Pediatric Gastroenterology, Hepatology and Nutrition Unit, Astrid Lindgren Children's Hospital, Karolinska University Hospital, Stockholm, Sweden
[12]Theme of Children's Health, Karolinska University Hospital, Stockholm, Sweden
[13]Department of Gastroenterology, Sachs' Children and Youth Hospital, Stockholm, Sweden
[14]Department of Clinical Science and Education, Södersjukhuset, Karolinska Institutet, Stockholm, Sweden
[15]Present address: Tech Watch, VIB Headquarters, Flemish Institute for Biotechnology, Ghent, Belgium
[16]Senior author
[17]These authors contributed equally
[18]Lead contact
*Correspondence: jenny.mjosberg@ki.se
https://doi.org/10.1016/j.xcrm.2023.101038

## SUMMARY

Innate lymphoid cells (ILCs) are considered innate counterparts of adaptive T cells; however, their common and unique transcriptional signatures in pediatric inflammatory bowel disease (pIBD) are largely unknown. Here, we report a dysregulated colonic ILC composition in pIBD colitis that correlates with inflammatory activity, including accumulation of naive-like CD45RA$^{+}$CD62L$^{-}$ ILCs. Weighted gene co-expression network analysis (WGCNA) reveals modules of genes that are shared or unique across innate and adaptive lymphocytes. Shared modules include genes associated with activation/tissue residency, naivety/quiescence, and antigen presentation. Lastly, nearest-neighbor-based analysis facilitates the identification of "most inflamed" and "least inflamed" lymphocytes in pIBD colon with unique transcriptional signatures. Our study reveals shared and unique transcriptional signatures of colonic ILCs and T cells in pIBD. We also provide insight into the transcriptional regulation of colonic inflammation, deepening our understanding of the potential mechanisms involved in pIBD.

## INTRODUCTION

Pediatric inflammatory bowel disease (pIBD) includes Crohn's disease (CD) and ulcerative colitis (UC), which have common symptoms like abdominal pain, frequent bloody diarrhea, and, for children with CD, sometimes also growth retardation.[1] Around 25% of patients with IBD have been diagnosed before the age of 18 years, and the overall prevalence of pIBD is


This is an open access article under the CC BY license (http://creativecommons.org/licenses/by/4.0/).

increasing.[2] PIBD is characterized by more severe symptoms and a more extensive disease at onset than adults, and the CD phenotype shows less isolated ileal manifestations and more colonic inflammation compared with adult patients with CD.[2] The etiology and pathogenesis of IBD is considered multifactorial. Host genetic as well as environmental factors may result in a dysregulated immune response and dysbiosis that is believed to drive chronic inflammation.[3]

T cells are implicated in intestinal inflammation in both mouse models[4] and in humans, where conventional ($CD4^+$ and $CD8^+$)[5–7] as well as unconventional T cells, including innate-like, γδ T cells and mucosal invariant (MAIT)[8,9] T cells, show dysregulated composition. In patients with IBD, biologic treatments antagonizing immunomodulating factors that either activate T cells (interleukin [IL]-12/23, ustekinumab, etc.) or are expressed by T cells (α4β7 integrin; velodizumab, tumor necrosis factor [TNF]; infliximab, etc.) are effective in the majority of the patients.[10]

Innate lymphoid cells (ILCs), including natural killer (NK) cells, are viewed as the counterparts of adaptive T cells, which, in concert, execute specialized effector programs.[11] In the inflamed adult IBD colon and ileum, the mucosal ILC composition is altered, resulting in reduced frequencies of IL-22-producing $NKp44^+$ type 3 ILCs (ILC3s) and increased interferon (IFN)-γ-producing ILC1s[12,13] as well as IL-17-producing ILC3s.[14] However, it remains unknown whether these alterations in ILC frequencies are also present in pIBD.

Single-cell RNA sequencing (scRNA-seq) studies have provided transcriptional signatures of T cells in IBD mucosa of children and adults.[5,15–19] However, characterization of intestinal ILCs in parallel to T cells in pediatric patients with IBD has not yet been performed.

Here, we provide a comprehensive characterization of ILCs and T cells in colonic biopsies from newly diagnosed and treatment-naive pediatric patients with IBD colitis and non-IBD controls. Flow cytometry showed inflammation-specific alterations of ILC frequencies, including the accumulation of $CD45RA^+CD62L^-$ naive-like ILCs, which correlated with disease severity. scRNA-seq revealed gene co-expression networks that were shared between or unique to innate and adaptive lymphocytes. Finally, a nearest-neighbor-based approach provided unbiased means to identify the "most inflamed" or "least inflamed" lymphocytes in our dataset based on the histological inflammation score of the samples. Overall, our study provides a comprehensive immunophenotypic and transcriptional characterization of ILCs and T cells in pediatric patients with IBD colitis. We uncover previously unknown co-regulatory dynamics of innate and adaptive lymphocytes that potentially play a role in disease pathogenesis and may be targeted for therapeutic intervention.

## RESULTS

### Dysregulated colonic ILC composition correlates with inflammatory activity in pIBD colitis

To characterize ILCs in pediatric patients with newly diagnosed IBD, we collected colonic biopsies from endoscopically inflamed and non-inflamed sites from 19 treatment-naive patients with CD or UC colitis (Table S1). Pediatric patients who underwent colonoscopy because of intestinal symptoms but did not have IBD or other endoscopic inflammation served as the non-IBD control group (n = 10). For the patients with IBD, endoscopic inflammation was evaluated with the SWIBREG[20] local endoscopic disease activity score (denoted "endoscopic score") (Table S2), and local microscopic inflammation was evaluated with a modified version of the D′ Haens et al. histological score (denoted "histo score"; see STAR Methods).[21]

Human intestinal $CD127^+$ ILCs were analyzed by flow cytometry as previously described[13,22] (Figure S1A). The identity of ILC1s, ILC3s, and NK cells was confirmed by staining of lineage-defining transcription factors in both blood and colon samples (Figure S1B). In contrast to $CD3^+$ T cells and $CD56^+$ NK cells, the frequency of $CD127^+$ ILCs among lymphocytes was reduced in endoscopically inflamed IBD compared with non-IBD and non-inflamed IBD colon mucosa (Figure 1A). The majority of ILCs expressed the tissue residency marker CD69 (Figure S1C) but also showed increased expression of markers associated with naive T cells, CD45RA and CD62L (Figure S1C). While HLA-DR expression was elevated in pIBD ILCs, the expression remained unchanged in total $CD3^+$ T cells (Figure S1C). However, elevated HLA-DR expression has been previously detected in $CD4^+$ T cells in IBD.[5] Further phenotyping of pIBD T cells and NK cells can be seen in Figure S1C.

We detected a skewed ILC composition in endoscopically inflamed IBD versus non-IBD and non-inflamed IBD colon, as illustrated by increased frequencies of $NKp44^-$ ILCs and ILC1s, while $NKp44^+$ ILC3 frequencies were reduced (Figure 1B), similar to our previous findings in adult IBD.[13] There was a positive correlation between the endoscopic and histo scores and the frequencies of ILC2s and $NKp44^-$ ILCs, whereas the frequency of $NKp44^+$ ILC3s was inversely correlated with both endoscopic and histo scores (Figure 1D). Overall, our findings show that the mucosal ILC composition is dysregulated in patients with pIBD, displaying a pattern similar to what we previously reported for adult IBD.

### Single-cell transcriptional profiling of colonic ILCs, NK cells, and T cells in pIBD colitis

To characterize the transcriptional signatures of both innate and adaptive lymphocytes in pIBD, we performed 3′ 10× scRNA-seq of biopsies from six treatment-naive pediatric patients with newly diagnosed CD-colitis (total n = 12 samples) (Figure 2A; Table S3). To assess the transcriptional signatures of inflammation within the same patient, biopsies were obtained from the endoscopically least and most inflamed area of the colon and subsequently fluorescence-activated cell sorted (FACS) for $Lin^-CD127^+CD161^+$ (ILCs), $Lin^+CD127^-CD56^+$ (NK cells), and $CD3^+$ (T cells) (Figure S2A). After quality control, normalization, and integration of the datasets, 44,717 lymphocytes were obtained for analysis. Louvain clustering analysis allowed for the separation of the pre-sorted cell populations into 12 clusters, which were represented by all patients and samples (Figures 2B and S2B). Clusters were annotated based on signature genes (Figure 2C), where one cluster represented ILCs expressing *IL7R*, *ID2*, and *IL22*, two clusters represented different activation stages of NK cells expressing *ID2*, *ZBTB16*, *PRF1*, *GZMA*, and *IFNG*, and nine clusters represented subsets of $CD4^+$ and $CD8^+$ T cells. These included naive/central memory $CD8^+$ and $CD4^+$ T cells ($CD8^+$ and $CD4^+$ Tn/cm cells; *CD8*,

CellPress
OPEN ACCESS

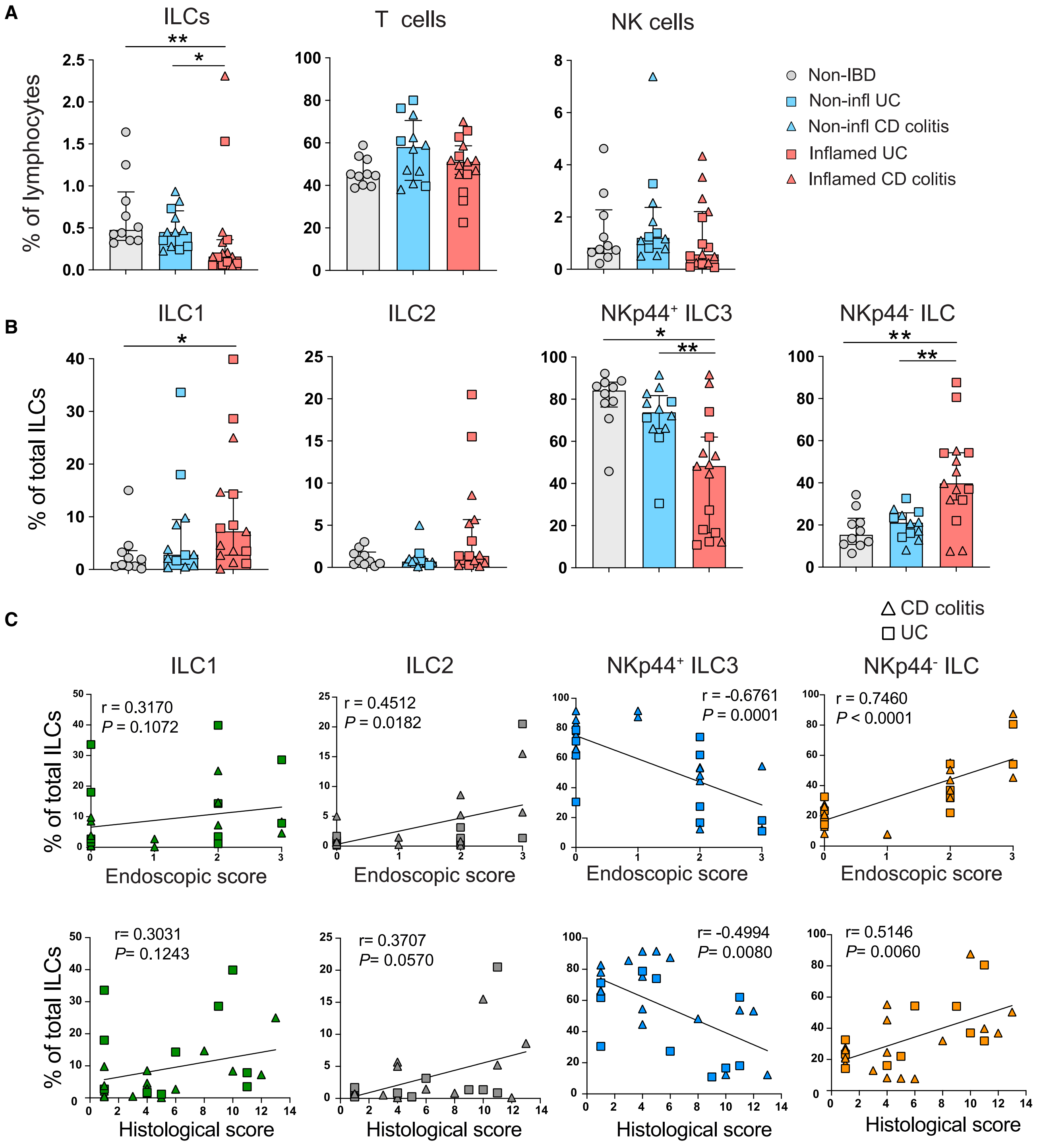


**Figure 1. Dysregulated colonic ILC composition correlates to inflammatory activity in pIBD colitis**

(A) Bar graphs showing the frequencies of total ILCs, T cells, and NK cells (gated as shown in Figure S1A) in non-IBD (n = 10), endoscopically non-inflamed (n = 12), and endoscopically inflamed (n = 15) pIBD colon samples. A total of 8 samples were derived from paired inflamed and non-inflamed samples. Frequencies shown out of $CD45^+$ lymphocytes.

(B) Bar graphs showing the frequencies of ILC1s, ILC2s, $NKp44^+$ ILC3s, and $NKp44^-$ ILCs (gated as shown in Figure S1A) in non-IBD (n = 10), endoscopically non-inflamed (n = 12), and endoscopically inflamed (n = 15) pIBD colon samples. Frequencies shown out of total ILCs.

(C) Correlation plots between frequencies of ILC1s, ILC2s, $NKp44^+$ ILC3s, and $NKp44^-$ ILCs and the endoscopic and histological scores determined for each of the endoscopically non-inflamed (n = 12) and endoscopically inflamed (n = 15) pIBD colon samples.

Statistical significances in (B) were detected with Mann-Whitney test. Bar graphs are shown as median ± interquartile range (IQR). Spearman's rank correlation test was applied for assessing correlations between variables. In (A)–(C), cells from each donor were analyzed in independent experiments.

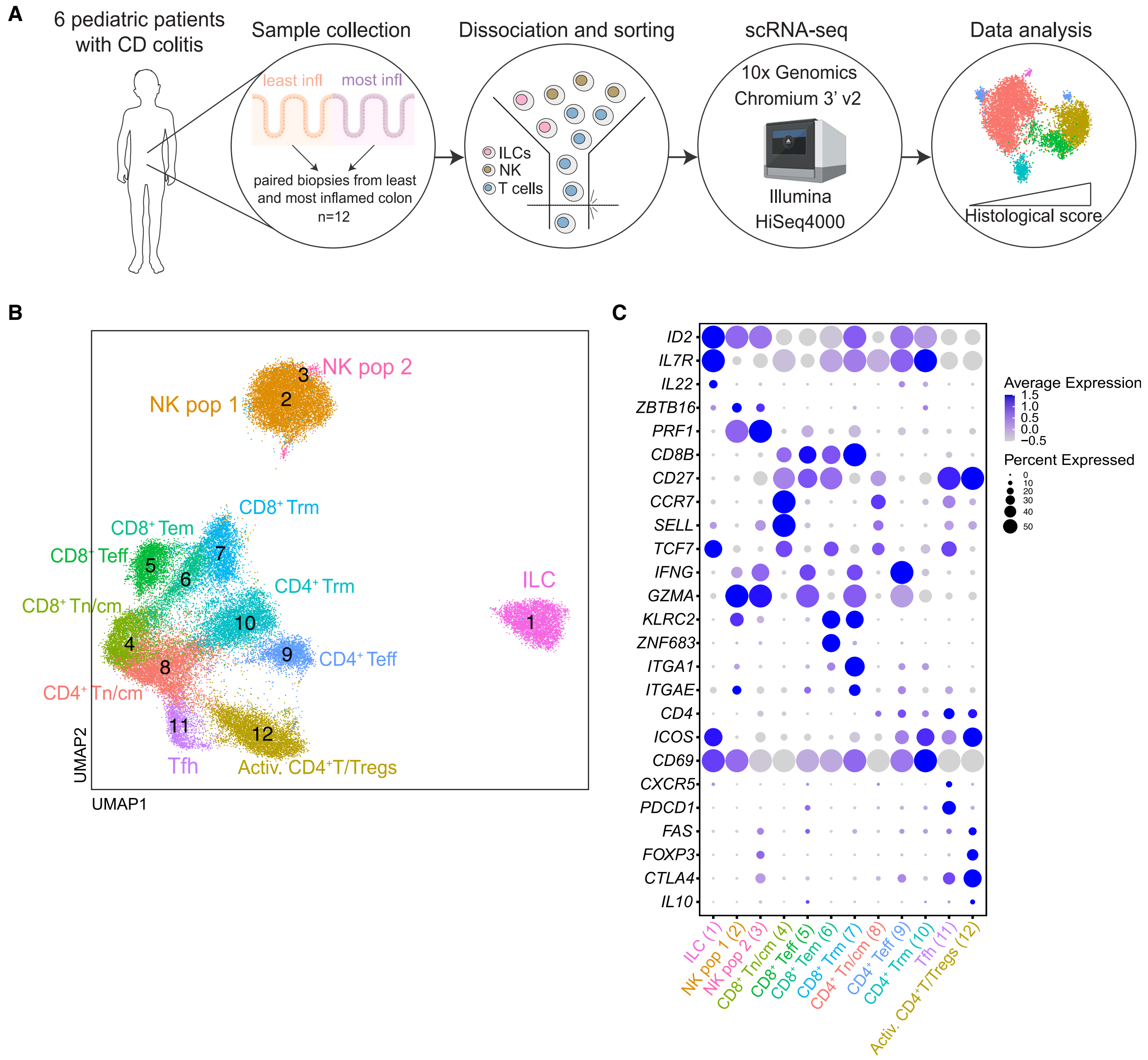


**Figure 2. Single-cell transcriptional profiling of colonic ILCs, NK cells, and T cells in pIBD colitis**

(A) Schematic illustration of the scRNA-seq workflow of the total 12 paired colonic biopsy samples obtained from the endoscopically least and most inflamed areas of the 6 pediatric patients with CD-colitis (n = 6 donors).

(B) UMAP visualization of the scRNA-seq data showing the unbiased clustering of total 44 717 ILCs, NK cells, and T cells.

(C) Dotplot displaying expression of lineage-identity genes for the annotation of the clusters in (B).

In (A)–(C), cells from each donor were analyzed in independent experiments.

*CD4*, *CCR7*, *SELL*, *TCF7*, and *CD27*); CD8$^+$ T effector cells (CD8$^+$ Teff cells; *CD8B*, *PRF1*, *GZMA*, and *IFNG*); CD8$^+$ T effector memory cells (CD8$^+$ Tem cells; *CD8B*, *KLRC2*, and *ZNF683*); CD8$^+$ T resident memory cells (CD8$^+$ Trm cells; *CD69*, *ITGA1*, and *ITGAE*); CD4$^+$ Teff cells (*CD4*, *IFNG*, and *GZMA*); CD4$^+$ Trm cells (*CD4*, *CD69*, and *ICOS*); T follicular helper cells (Tfh cells; *CD4*, *CXCR5*, *PDCD1*, and *FAS*); and activated CD4$^+$T/regulatory T cells (Activ. CD4$^+$T/Tregs; *CD4*, *CTLA4*, *ICOS*, *FOXP3*, and *IL10*) (Figures 2B and 2C).

Altogether, scRNA-seq revealed an overall diverse landscape of innate and adaptive lymphocytes in the pediatric colonic mucosa.

## Gene module analyses of colonic innate and adaptive lymphocytes reveal subset-specific and shared programs of co-expressed genes

Complex biological processes like inflammation are orchestrated by co-expressed gene networks. In addition, ILCs and

CellPress
OPEN ACCESS

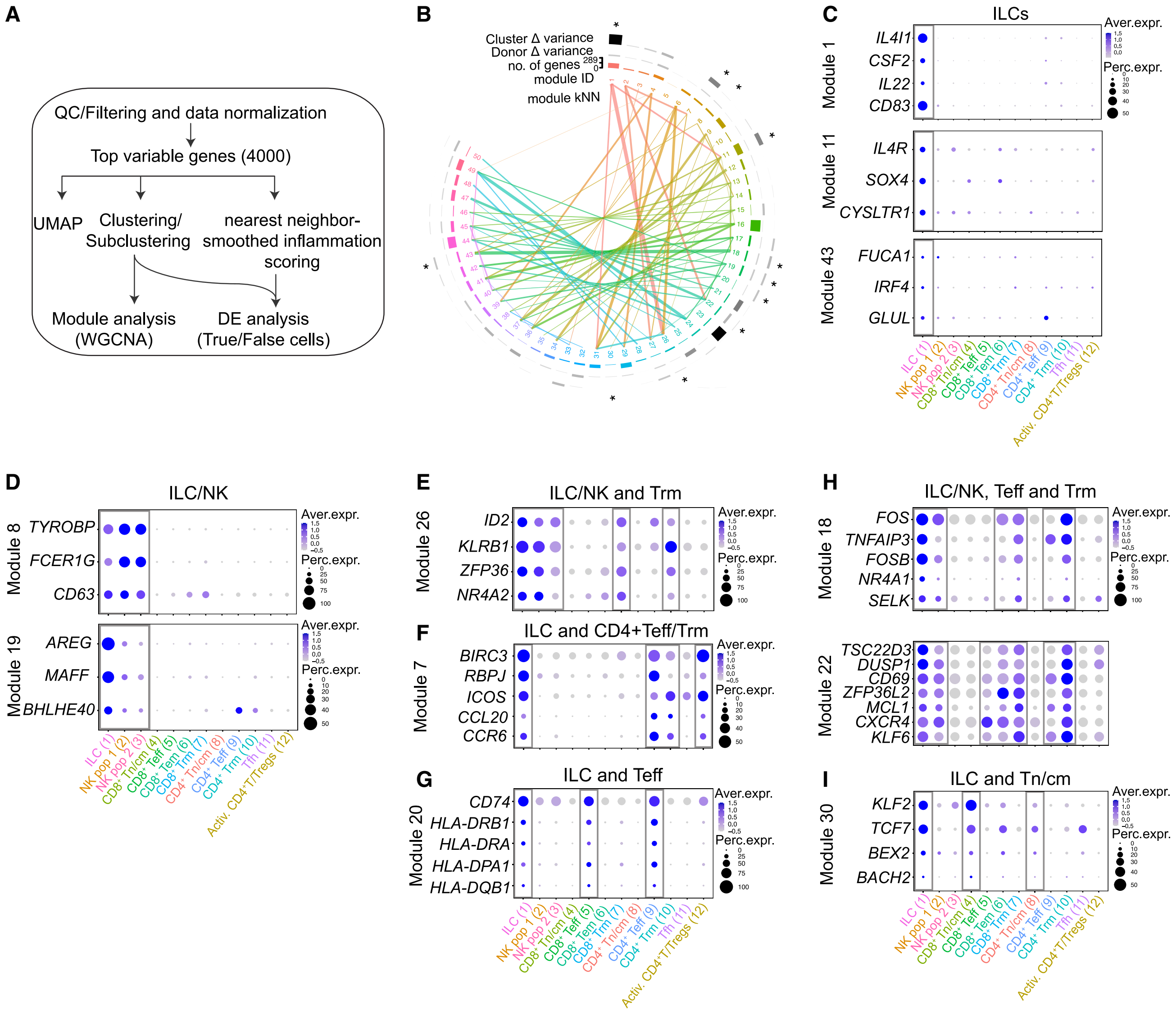


**Figure 3. Gene module analyses of colonic innate and adaptive lymphocytes reveal subset-specific and shared programs of co-expressed genes**

(A) Summary flow chart used for the analysis of scRNA-seq data and subsequent gene module and nearest-neighbor analysis.

(B) Circular dendrogram illustrating the number and composition of each module and the contribution of each module to discriminating cell clusters and donors. Connecting lines represent modules with highly correlated expressions (kNN [k nearest neighbor]). Modules selected for analysis and described in the text are marked with an asterisk.

(C–I) A selection of genes in each module marked with an asterisk in (B) illustrated by dotplots. The expression of the modules across the annotated clusters is shown in Figure S3.

T cells largely overlap in terms of phenotype and functions, suggesting coordinated gene regulation. To identify such patterns of gene expression and regulation dynamics, we performed weighted gene co-expression network analysis (WGCNA),[23,24] where we used the top 4,000 variable genes to discern 50 modules of co-regulated genes among the lymphocytes (Figure 3B). With this approach, we identified gene networks that were either confined to a specific cell type or shared across cell types. More specifically, we identified three modules (1, 11, and 43) preferentially expressed by the ILC cluster (Figures 3C and S3A). Intriguingly, module 1 showed coordinated expression between key ILC cytokine genes *IL22*, *CSF2* (encoding GM-CSF), the co-stimulatory molecule *CD83*, and *IL4I1*, the latter encoding a secreted L-amino acid oxidase reported to promote aryl hydrocarbon receptor (AHR) activation while limiting Th17 proliferation.[25,26] The expression of *SOX4*, *IL4R*, and *CYSLTR1* (encoding the cysteinyl leukotriene receptor) in module 11 showed that although a distinct cluster of ILC2s could not be identified in our data, ILCs expressing transcripts with known regulatory function in ILC2s/Th2 cells are present in the pIBD colon.

Two gene modules were shared between the two innate lymphocyte clusters (ILCs and NK cells; modules 8 and 19), including the cytotoxicity-associated adapter proteins *TYROBP* and *FCER1G*, as well as the effector molecule *AREG* that was expressed together with *BHLHE40*, encoding a transcriptional regulator previously reported to control cytokine production in T cells[27,28] (Figures 3D and S3B). Highlighting the shared features of ILCs and T cells, module 26 was coordinately expressed by ILCs and Trm cells, including innate-like genes (*ID2* and *KLRB1*) and genes associated with tissue residency (*ZFP36* and *NR4A2*) (Figures 3E and S3C). Module 7 was shared by ILCs and CD4$^+$ Teff cells, Trm cells, and Activ. T cells and included genes typically expressed by Th17 cells (*RBPJ*, *ICOS*, *CCL20*, and *CCR6*) (Figures 3F and S3D). In line with previous reports showing antigen-presenting features of ILCs,[29–32] module 20 contained a set of transcripts associated with antigen presentation (*HLA-DRA* and *HLA-DPA1*) that were shared by ILCs and Teff cells (Figures 3G and S3E). Additional shared signatures between ILCs and T cells were related to genes that regulate tissue residency (*FOS* and *NR4A1*; module 18) as well as quiescence (*TSC22D3*, *DUSP1*, *ZFP36L2*, and *KLF6*; module 22) (Figures 3H and S3F), the latter recently shown to be expressed by plastic skin ILCs in a mouse model of psoriasis.[33] Lastly, module 30 showed coordinated expression of *KLF2* and *TCF7*, genes typically expressed by naive T cells. However, module 30 was highly expressed by ILCs and naive T cells, indicating the presence of ILCs with naive features as previously described in the adult gut[24,34] (Figures 3I and S3G).

Overall, these findings reveal subset-specific and common gene signatures that may regulate ILCs and T cells.

## Gene modules associate with ILC subclusters and identify quiescent/naive-like ILCs

To better understand the expression pattern of unique and shared gene modules among ILCs, we focused our analysis only on the ILCs. Reclustering of ILCs revealed 7 transcriptionally distinct clusters that were annotated by differential expression (DE) analysis (Figures 4A, 4B, S4A, and S4B). Interestingly, the pIBD ILC subclusters displayed a similar degree of heterogeneity with ILCs from adult IBD colon, while tonsillar ILCs were less heterogeneous (Figures S4C and S4D). Intriguingly, the module 1 genes *CD83*, *IL4I1*, *IL22*, and *CSF2* were confined to ILC subcluster 3 (Figures 4C and S4E), but these genes associated poorly with expression of the key ILC3 genes *NCR2* (Figure 4C) and *RORC* (Figure S4F), which were widely expressed among the ILC clusters. We further identified that module 20 genes (*HLA-DPA1*, *HLA-DRA*, and *HLA-DQB1*) were uniquely expressed (Figures 4B, 4D, and S4E) by subcluster 7, suggesting that they represent antigen-presenting ILCs. Furthermore, subcluster 1 expressed many genes in modules 18 and 22, including *TSC22D3*, *DUSP1*, and *CD69* (Figures 4B, 4E, and S4E), previously associated with quiescent and tissue-resident ILCs.[33] Interestingly, in line with our previous report,[34] we identified a *SELL*-expressing ILC subcluster 4 (Figures 4B and 4F), with low expression of *NCR2*, *HLA-DR*, and *RORC* transcripts (Figures 4B, 4D, and S4F), likely representing naive-like ILCs enriched in adult IBD. Genes in the "naivety" module 30 (Figure 3I) were not solely expressed by subcluster 4. In fact, *TCF7* was homogenously expressed across all ILCs, and *KLF2* was also expressed by subcluster 1 (Figures 4B and 4G). These data indicated the presence of at least two subclusters of ILCs with features of naive T cells. To determine the developmental relationships between the ILC subclusters, we performed trajectory analysis with Monocle 3 using subcluster 4 (Figure 4H) or 1 (Figure S4G) as the root, respectively. We found that regardless of the root, subclusters 3 (*IL22*-expressing effector ILCs), 4 (naive ILCs), 1 (tissue resident/quiescent ILCs), and 6–8 (ILC1-like ILCs, HLA-DR$^+$ ILCs, SOX4$^+$ ILCs) branch separately from each other, with cluster 0 as the branching point.

To better characterize the naive/quiescent ILCs in pIBD colon, we stained ILCs for markers of activation/differentiation. We have recently described that the less-differentiated and less-activated (NKp44$^-$HLA-DR$^-$) ILCs in human tissues, including adult IBD gut, form a heterogeneous population that includes naive-like/quiescent ILCs marked by the presence of surface CD45RA expression.[34] Confirming and extending our previous findings, CD45RA also marked such cells in pIBD (Figure 4I). As we observed in the adult IBD gut, surface CD62L was not expressed on this population despite the presence of *SELL* transcripts (Figure 4J). Similarly to adult IBD, the frequency of these CD45RA$^+$CD62L$^-$ ILCs (referred to as CD45RA$^+$ ILCs) was increased in the endoscopically inflamed pIBD gut samples, and their frequency was positively correlated with the local endoscopic as well as histo score (Figures 4K and 4L).

In summary, our analyses reveal gene networks confined to certain ILC subsets, including a naive-like module. Using previously described surface markers that identify naive-like/quiescent ILCs in tissues, we observed an accumulation of CD45RA$^+$ ILCs in the inflamed gut of pediatric patients with IBD that correlated with the degree of inflammation.

## Nearest-neighbor-smoothed histo scores reveal the most and least inflammatory cells

Next, we set out to explore inflammation-related transcriptional signatures of innate and adaptive lymphocytes. During endoscopy, the grade of inflammation is evaluated based on the presence of visual signs.[35] In contrast, histological findings more precisely assess both the acute and chronic cellular changes occurring in the intestinal epithelium and lamina propria.[36] We were therefore interested in defining the transcriptional signatures of these cellular changes with scRNA-seq (Figures 5A and 5B). Indeed, when plotting the histo scores on the uniform manifold approximation and projection (UMAP), we could observe overrepresentation of lymphocytes from samples with high histo scores in certain clusters (Figure 5C). However, this crude representation did not consider donor representability. Therefore, we repurposed a nearest-neighbor-based smoothing method deployed elsewhere.[37–39] Here, each cell was assigned the average histo scores of the 100 nearest-neighbor cells in the Euclidean space, which was created by a 100-dimensional principal-component analysis based on the 4,000 most variable genes (Figure 5D). To balance out the number of neighbors for each histological group (defined below), we conducted subsampling of the data so that all three groups consisted of the same

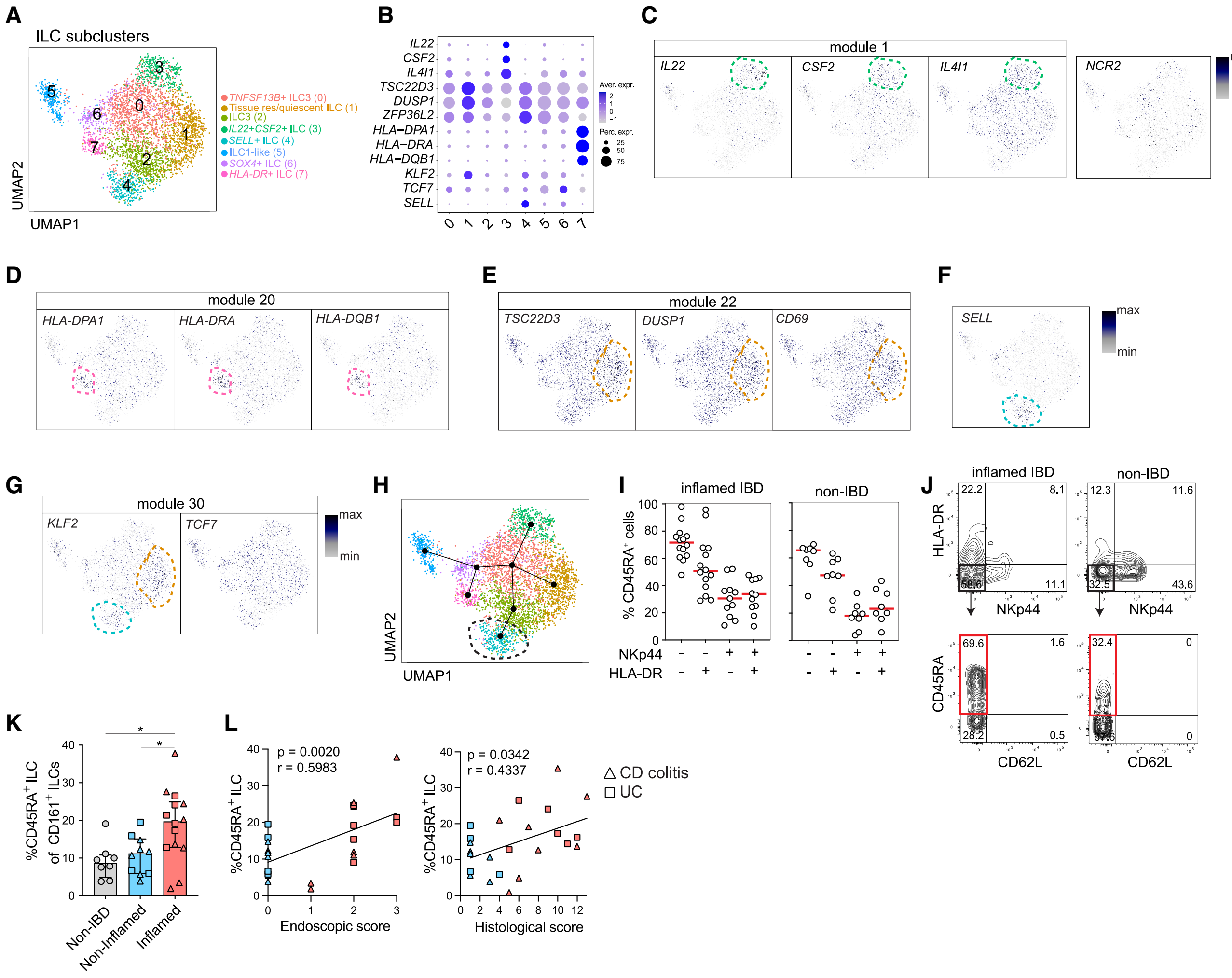

**Figure 4. Gene modules associate with ILC subclusters and identify quiescent/naive-like ILCs**

(A) UMAP visualization of ILC subclusters after reclustering main cluster 1 shown in Figure 2B.

(B) Dotplot showing selected ILC-module genes (Figures 3D–3I). The expression is shown within the annotated subclusters of ILCs depicted in (A).

(C–G) UMAP visualization of selected ILC-module genes in the ILC subclusters. Dotted lines mark the subcluster where the depicted gene is highly and differentially expressed. A list of differentially expressed genes (DEGs) per ILC subcluster is shown in Figure S4A.

(H) Developmental trajectories of ILC subclusters performed with Monocle 3. Subcluster 4 (dotted circle) was used as the root.

(I) Frequencies of CD45RA$^+$ ILCs among ILCs expressing combinations of HLA-DR and NKp44 in inflamed pIBD (n = 12–14) and non-IBD (n = 8) colon biopsies (bar graphs are shown as median). The samples were analyzed in independent experiments.

(J) Representative flow cytometry plots of CD45RA versus CD62L (marked in red is the CD45RA$^+$ ILC subset), gated out of NKp44$^-$HLA-DR$^-$ ILCs, in inflamed pIBD and non-IBD colon biopsies.

(K) Frequency of CD45RA$^+$ ILCs out of CD161$^+$ ILCs in non-IBD (n = 8), endoscopically non-inflamed (n = 10), and endoscopically inflamed pIBD (n = 14) colon biopsies. Mann-Whitney test ($p < 0.05$), mean with SD. The samples were analyzed in independent experiments.

(L) Correlation plots (Spearman, $p < 0.05$) between the frequency of CD45RA$^+$ ILCs and the endoscopic and histological scores determined for each of the endoscopically non-inflamed (n = 8) and endoscopically inflamed (n = 14) pIBD colon samples.

number of neighbors (Figure S5A). Distribution of lymphocytes across tissues with different histo scores allowed for categorization of the cells into three groups: least inflamed (histo score ≤ 4), "intermediate" (histo score = 5–8), and most inflamed lymphocytes (histo score ≥ 9) (Figure 5E). Visualization of the smoothed histo scores revealed enrichment of the most inflamed and least inflamed lymphocytes in certain clusters (Figures 5F and S5B). The most inflamed samples were mostly derived from 4 samples (Figure S5C). However, similar enrichment was predicted when batch correction was applied (Figure S5D) and also when we performed differential abundance analysis with DAseq (Figure S5E).[38] The most inflamed lymphocytes were predominantly NK cells (cluster 2), CD8$^+$ Teff cells (cluster 5), CD4$^+$ Tef cells (cluster 9), and Activ. T cells/Tregs (cluster 12) (Figure 5F). Conversely, the least inflamed cells were enriched in ILCs (cluster 1), CD8$^+$ Tn/cm cells (cluster 4), CD4$^+$ Tn/cm cells (cluster 8), CD8$^+$ Trm cells (cluster 7), and CD4$^+$ Trm cells (cluster 10).

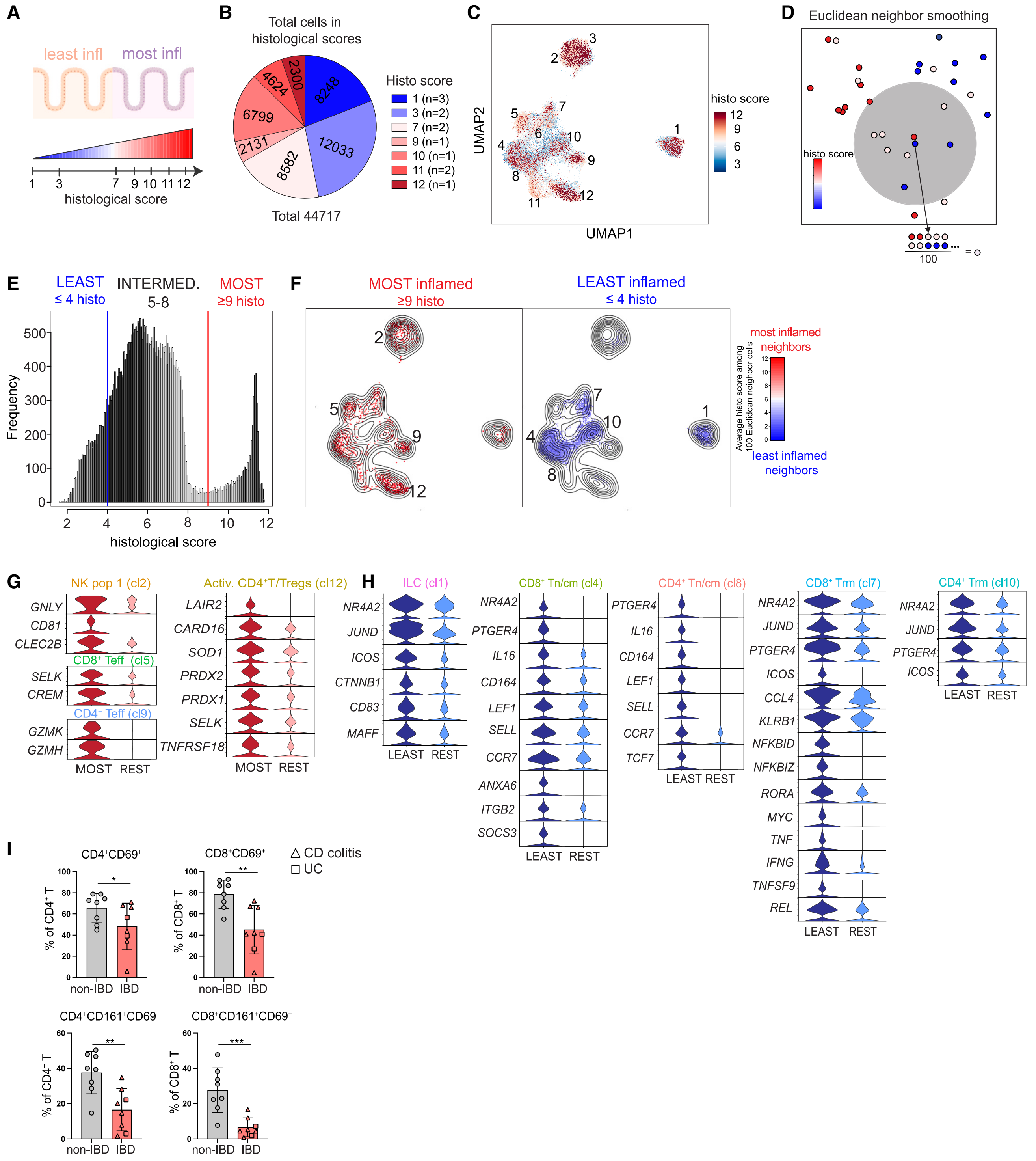


**Figure 5. Nearest-neighbor-smoothed histological scores reveal the most and least inflammatory cells**

(A) Schematic illustration showing the histological scores from the endoscopically least and most inflamed paired colonic biopsies used for scRNA-seq.

(B) Pie chart showing the total number of cells from areas of different histological scores from all 12 pIBD colon biopsy samples.

(C) The raw histological score of each cell derived from each sample depicted onto the main UMAP from Figure 2B.

(D) Schematic illustration of the Euclidean neighbor smoothing. Each cell was given the average histological scores of its 100 transcriptionally closest neighbors. These smoothed histological scores were used for the subsequent analysis.

(legend continued on next page)

CellPress
OPEN ACCESS

To uncover transcriptional signatures of the most inflamed lymphocytes, we performed DE analysis in the most inflamed cells (shown as MOST) versus the rest of the cells (shown as REST) within each cluster. A total of 96 genes were upregulated in the most inflamed cells with 35 genes being shared among the clusters (Table S4). The most inflamed NK cells showed elevated expression of the transcript for the antimicrobial molecule granulysin (*GNLY*) and genes involved in immunoregulatory interactions (*CD81* and *CLEC2B*), while the most inflamed $CD8^+$ T cells expressed the selenoprotein K (*SELK*) and the cAMP regulator *CREM* (Figure 5G). Interestingly, the most inflamed $CD4^+$ Teff cells displayed a cytotoxic signature with expression of the granzymes *GZMK* and *GZMH* (Figure 5G), while the most inflamed Activ. $CD4^+$ T/Tregs upregulated genes involved in redox balance (*SELK*, *PRDX1*, *PRDX2*, and *SOD1*), the caspase inhibitor *CARD16*, and the inhibitory molecules *LAIR2* and *TNFRSF18* (GITR) (Figure 5G). Similarly, to transcriptionally characterize the least inflamed lymphocytes, we performed DE analysis in the least inflamed cells (shown as LEAST) versus the rest of the cells (shown as REST) (Figure 5H). The least inflamed lymphocytes demonstrated larger transcriptional differences compared with the most inflamed lymphocytes with an upregulation of 226 genes, of which 70 were shared among the clusters (Table S4). Transcripts related to tissue residency such as *NR4A2* and *JUND* as well as *PTGER4* and *ICOS* were commonly expressed by the least inflamed cells across several clusters (Figure 5H). Additionally, the least inflamed ILCs revealed expression of *CD83*, involved in dendritic cell maturation as well as lymphocyte activation.[40] The least inflamed $CD8^+$ and $CD4^+$ Tn/cm cells expressed molecules related to their identity (*SELL*, *LEF1*, *CCR7*, *TCF7*) like the least inflamed Trm cells that additionally showed overexpression of genes associated with tissue residency (*ICOS*, *JUND*, *NR4A2*, and *PTGER4*) (Figure 5H). The least inflamed $CD8^+$ Trm cells additionally upregulated genes related to "innateness" (*RORA*, *KLRB1*) and effector functions (*IFNG*, *TNF*, *CCL4*) (Figure 5H). To validate these findings, we performed flow cytometry using intestinal specimens of adult patients with and without IBD (Table S5). We showed that, indeed, $CD4^+$ Trm and $CD8^+$ Trm cells (defined as $CD69^+$ and/or $CD161^+$) were lower in frequency in IBD, as predicted by the nearest-neighbor analysis (Figure 5I). The prediction of ILCs being mostly represented in the least inflamed landscape was additionally confirmed by our flow cytometry analysis of total ILCs (Figure 1A).

Collectively, based on nearest-neighbor analysis of transcriptional profiles and histo scores, we identified most inflamed and least inflamed lymphocytes with potential roles in colonic inflammation. The genes expressed by these cells deserve further functional exploration to assess their usefulness as targets for immunotherapy in IBD.

## DISCUSSION

In our study, we combined high-dimensional single-cell transcriptional and immunophenotypic analyses to comprehensively characterize innate and adaptive lymphocytes from colonic biopsies obtained from treatment-naive pediatric patients with newly diagnosed IBD colitis. We focused on pIBD as it generally shows a more aggressive phenotype and allows for studies of disease mechanisms that are less influenced by diagnostic delay,[41] age-related environmental exposures, for example smoking, and co-morbidities compared with adults. A number of studies have investigated IBD-associated patterns of T cells and ILCs in human tissues at the single-cell level.[5,15,16,18,19] However, these studies describe discrete cell populations of either ILCs or T cells without directly comparing these two cell types. Therefore, we complemented our protein analysis of ILCs with in-parallel single-cell transcriptional profiling of ILCs, NK cells, and T cells to uncover transcriptional networks that govern both shared and unique properties. Through our approach, we provide insights into ILC and T cell biology in pIBD. Understanding the regulation of these transcriptional networks could possibly lead to the development of therapies for IBD.

Flow cytometry revealed a dysregulated composition of mucosal ILCs in pIBD that correlated with the inflammatory activity. More specifically, we observed a reduction of $NKp44^+$ ILC3s and an increase of $NKp44^-$ ILCs and ILC1, the latter which could, at least to some extent, be explained by ILC3-ILC1 plasticity as described for adult IBD.[12] However, it is currently unclear how well the FACS-based definition of human ILCs correlates with transcriptional and functional features of ILCs in the gut mucosa. Similar to our FACS definition of ILC1, scRNA-seq analysis revealed an ILC1-like cluster expressing *TBX21* (encoding T-bet) but low *KIT* (encoding CD117) and *RORC*. These cells likely represent the previously described $CD127^+$ ILC1s[12] and not the intraepithelial $CD103^+$ ILC1s, as those lack CD127 expression.[42] Expression of *NCR2*, encoding NKp44, was seen in several ILC subclusters, indicating transcriptional heterogeneity among $NKp44^+$ ILC3s. It has been well described that human $NKp44^+$ ILC3s harbor IL-22-producing ILCs[43] and that ILC3-derived IL-22 is crucial for gut homeostasis in mice.[44] However, here we show that *IL22* transcription is confined to a specific ILC subcluster with relatively low expression of *RORC*. This is biologically possible as RORC-deficient patients are still capable of IL-22 production.[45] Of note, *IL22* was co-transcribed with other effector molecules such as *CSF2*, *CD83*, and *IL4I1* in a gene module that was preferentially expressed in ILCs compared with T cells, potentially reflecting the more rapid response of ILCs compared with T cells. Notably, *IL4I1* can activate AHR

(E) Histogram showing the cell type distribution across the histological scores derived from the nearest-neighbor analysis. Cells were categorized into 3 groups: $\leq 4$ (least inflamed), 5–8 (intermediate), and $\geq 9$ (most inflamed).

(F) Visualization of cells from the nearest-neighbor smoothing on the main UMAP with cells derived from the $\geq 9$ histological (histo) score (most inflamed) and $\leq 4$ histo score (least inflamed) groups, which also have neighbors from at least 5 samples.

(G and H) Violin plots showing the expression level of selected DEGs (log2 fold change [FC] > 0.10 and adjusted p value [adj. p value] < 0.05) upregulated in the most (G) and least (H) inflamed cells versus the rest of the cells in each cluster.

(I) Frequencies of $CD4^+CD69^+$, $CD4^+CD161^+CD69^+$, $CD8^+CD69^+$, and $CD8^+CD161^+CD19^+$ cells in colonic adult non-IBD (n = 8) and IBD samples (n = 8). Mann-Whitney test ($p < 0.05$), mean with SD. The samples were analyzed in two independent experiments.

ligands in tumor cells via tryptophan metabolism,[25] which in turn supports IL-22 production in ILCs.[46] Thus, *IL4I1* may promote AHR in ILC3s. Additional studies are needed to investigate how *IL4I1* or other co-expressed molecules regulate IL-22 production by intestinal human ILCs.

Although ILC2s represent a large population in the mouse intestine[47] and human fetal intestine,[48] the healthy human adult intestine is largely devoid of conventional CD127$^+$ ILC2s,[48,49] similar to what we also observed in our flow cytometric and scRNA-seq analysis of pIBD colon tissue. Although it is possible that intestinal ILC2s show an unconventional profile that we excluded with our current sorting gating strategy, we identified an ILC-restricted gene module containing genes associated with the regulation of ILC2s. This included positive regulators such as *IL4R* and *CYSTL1* but also *SOX4*, which can suppress Th2 differentiation.[50] Thus, we speculate that the absence of a transcriptionally distinct subset of CD127$^+$ ILC2s in the human intestine could be due to gut environmental factors that suppress ILC2s with a conventional phenotype. This topic requires further investigation.

One of the most striking overlaps between innate and adaptive lymphocytes in our analysis was the expression of a gene module containing genes associated with tissue residency including *CD69*, *CXCR4*, and *KLF6*. This gene module was particularly highly expressed in ILCs and Trm cells, indicating similar programs for tissue residency in innate and adaptive lymphocytes, a topic that deserves further investigation. However, we also noted that this module included genes associated with cellular quiescence, *TSC22D3*, *DUSP1*, and *ZFP36L2*.[33] These genes were highly transcribed in a *SELL*-expressing ILC subcluster (*ZFP36L2*) and in another ILC subcluster (*TSC22D3* and *DUSP1*), which co-transcribed the transcription factor *KLF2*, typically expressed by naive T cells. The *SELL*-expressing ILC subcluster lacked features of mature ILCs including *RORC*, *NCR2*, *TBX21*, or *EOMES*, reminiscent of the CD62L$^+$ naive-like ILCs we recently described in tonsils.[34] The second naive/quiescent cluster, which lacked expression of *TBX21* and *EOMES*, likely represented the quiescent/naive-like ILCs that we previously reported in human tonsil and adult gut,[34] where we showed that these less-differentiated quiescent ILCs are marked by the presence of CD45RA expression and, despite expression of *SELL* transcripts, the absence of CD62L.[34] The frequency of this subset was increased in the inflamed gut of adult patients with IBD, and they could differentiate to ILC1s and IL-22-producing ILC3s.[34] In the present study, we extended this finding to show that CD45RA$^+$ ILCs accumulate in the inflamed gut of pediatric patients with IBD as well. These cells could be ready for *trans*-differentiation, as previously shown in a mouse model of psoriasis where ILC3s accumulated by *trans*-differentiation of ILC2s via a quiescent-like state of ILCs.[33]

We further identified a gene module that was commonly expressed by a distinct subcluster of ILCs and T cells and that contained transcripts of major histocompatibility complex (MHC) class II molecules involved in antigen presentation. Indeed, we and others have previously shown that ILCs can acquire antigen-presenting properties that in turn can be suppressed by the human tumor microenvironment[29] and the gut microenvironment in mice.[31] However, the antigen-presenting capacity of ILCs in the context of human IBD, in the presence of a more complex microbiota compared with transgenic mice, remains underexplored. Hence, it is possible that ILCs could orchestrate CD4$^+$ T cell responses during gut inflammation in humans, similarly to what was described in a mouse model of acute colitis.[30]

To unravel inflammation-driven transcriptional changes in innate and adaptive lymphocytes, we made use of the histological inflammation score of our samples rather than grouping them in two endoscopically defined groups with variable histo scores. A nearest-neighbor-based smoothing of histo scores identified so-called least inflamed cells that were mostly defined as ILCs, CD4$^+$ and CD8$^+$ Tn/cm cells, and innate-like CD8$^+$ Trm cells. The least inflamed colon areas of patients with pIBD may represent tissue that was previously inflamed and now healed or tissue that is yet unaffected by inflammation. Regardless, the transcriptomes of least inflamed cells represent a footprint of innate and adaptive lymphocyte homeostasis in the colon. This includes genes associated with the lineage identity of ILCs, CD4$^+$/CD8$^+$ Tn/cm cells, CD4$^+$ Trm cells, and innate-like CD8$^+$ Trm cells, suggesting that maintained function of these cells is critical for immune homeostasis in the gut and that loss of these cells is associated with gut inflammation. In contrast, the most inflamed cells were dominated by NK cells, cytotoxic CD4$^+$ T cells, Activ. CD4$^+$ T cells/Tregs, and CD8$^+$ Teff cells.

It is possible that the compositional differences in least and most inflamed cells is due to recruitment of lymphocytes from the blood to inflamed areas of the colon. However, it could also be due to plasticity and/or differentiation of cells. Plasticity of ILCs and T cells is well described, and for ILCs, conversion of non-cytotoxic human ILCs to NK cells has been demonstrated *in vitro*.[51] Most inflamed NK cells upregulated transcripts of the antimicrobial protein *GNLY* and *CLEC2B*, the latter mediating NK cell activation via ligation to NKp80.[52] *GNLY* has been shown to be released in response to infections and to facilitate antigen presentation in dendritic cells (DCs) via Toll-like receptor 4 (TLR4).[53] Interestingly, *GNLY*-expressing CD94$^+$CD127$^+$ ILCs are expanded in patients with CD,[54] illustrating the fluidity in ILC-NK cell functions and the possibly that ILCs are reduced at the expense of NK cells during gut inflammation. Similarly, the underrepresentation of CD4$^+$ Tn/cm and CD4$^+$ Trm cells among the most inflamed cells could be due to differentiation to cytotoxic CD4$^+$ T cells and/or Activ. T cells/Tregs. Several studies have described the presence of cytotoxic CD4$^+$ T cells (CD4$^+$ CLT cells) in IBD,[55,56] but their origin remains unknown. We identified a CD4$^+$ T cell subset expressing *GZMH* and *GZMK* in inflamed IBD. Interestingly, this is in line with a recent report describing that CD4$^+$ CTL cells exhibit a diverse granzyme expression in nasal polyp tissue, including a subset that expressed these non-conventional granzymes.[57] GZMK and GZMH expression is seen in CD8$^+$ T cells with limited TCR repertoire and expression of exhaustion markers in aging[58] and autoimmunity,[59] suggesting that the CD4$^+$ T cell subset that we detect herein might be a highly differentiated/exhausted subset. Future studies should be aimed to address the origin and function of these CD4$^+$ CLT cells and Activ. T cells/Tregs in the context of gut mucosal inflammation in IBD.

We also noted accumulation of CD8$^+$ Teff cells among the most inflamed cells. While it is tempting to speculate that these cells could be derived from the CD8$^+$ Tn/cm and/or CD8$^+$ Trm cells among the least inflamed cells, further work is required to

address this. The role for the $CD8^+$ T cell compartment is conflicting in IBD. A single-cell atlas study of $CD8^+$ T cells in UC described innate features of *CD8*$^+$*IL26*$^+$ T cells,[18] while another study reported reduced frequencies of cytotoxic IFN-$\gamma^+$ $CD8^+$ T effector memory cells re-expressing CD45RA (TEMRA) cells in UC mucosa.[5] Similarly, we found increased expression of *IFNG*, *TNF*, and innate-like transcripts in the least inflamed IBD $CD8^+$ Trm cells, suggesting that these cells have important homeostatic functions in the gut.

In summary, we report that pediatric patients with IBD display dysregulated ILC frequencies that are similar to those observed in adult patients with IBD, suggesting a common pathogenic program in this aspect. Using a combination of high-dimensional single-cell analysis on the transcriptional and protein levels, we provide a roadmap of ILCs and T cells in pIBD, including transcriptional signatures associated with inflammation. Further mechanistic studies are necessary to understand whether and how these findings contribute to pathogenesis and how they could facilitate the design of immunotherapeutic targets.

### Limitations of the study

Although paired non-inflamed biopsies were used to infer inflammatory changes in the inflamed samples, the main limitation of the study is the lack of a non-symptomatic, healthy non-IBD control group in the single-cell analysis. Furthermore, one of the main transcriptional findings, on the reduced presence of Trm cells in colon inflammation, was validated on the protein level using samples from adult patients with IBD. However, we were not able to validate this, or other interesting transcriptional findings, in samples from patients with pIBD. Finally, since we performed our studies in samples from patients with ongoing inflammation, we cannot determine if the observed differences are a cause or consequence of IBD and what the role of these differences are in driving inflammation. In summary, the limitations of this study represent important aspects to address in future studies.

## STAR★METHODS

Detailed methods are provided in the online version of this paper and include the following:

- KEY RESOURCES TABLE
- RESOURCE AVAILABILITY
  - Lead contact
  - Materials availability
  - Data and code availability
- EXPERIMENTAL MODEL AND SUBJECT DETAILS
  - Human patients and study design
  - Ethical approval
  - Characterization and inflammation activity scoring of patients with pIBD
- METHOD DETAILS
  - Cell isolation from intestinal samples and blood
  - Flow cytometry and cell sorting
  - 10x genomics single-cell RNA sequencing
- QUANTIFICATION AND STATISTICAL ANALYSIS
  - Single-cell RNA sequencing data analysis
  - Weighted gene co-expression network analysis (WGCNA)
  - Euclidean neighbor-based smoothing of histology inflammation score
  - Statistical analyses

## SUPPLEMENTAL INFORMATION

Supplemental information can be found online at https://doi.org/10.1016/j.xcrm.2023.101038.

## ACKNOWLEDGMENTS

We would like to thank all the patients, and for the pediatric patients, also their parents, for their generous participation. Additionally, we thank all nurses, research nurses, and physicians that contributed to the study and the Flow Cytometry Core Facility, Department of Medicine Huddinge, the Bioinformatics and Expression Analysis core facility at Karolinska Institutet, and the VIB Nucleomics Core, Leuven, Belgium, for their essential help. Support by NBIS (National Bioinformatics Infrastructure Sweden) is gratefully acknowledged. P.C. is financially supported by the Knut and Alice Wallenberg Foundation as part of the National Bioinformatics Infrastructure Sweden at SciLifeLab. This work was supported by the European Crohn's and Colitis Organisation (ECCO) (no grant number) (to A.v.A.); the Swedish Research Council 2018-00384 and 521-2013-2791 (to J.M.); Cancerfonden 170082, 160664, and 190163 (to J.M.); the Foundation for Strategic Research FFL15-0120 (to J.M.); the Knut and Alice Wallenberg Foundation 4-1198/2016 (to J.M.); Karolinska Institutet 2-1536/202 (to J.M.); the European Research Council (ERC) under the European Union Horizon 2020 research and innovation program (grant agreement no. 850963, GUT-SEQ) (to J.M.); the Sigrid Jusélius Foundation (no grant number) (to T.S.); and the Finnish Pediatric Research Foundation (no grant number) (to T.S.).

## AUTHOR CONTRIBUTIONS

Conceptualization, J.M., H.R., E. Kokkinou, and A.v.A.; methodology, E. Kokkinou, T.S., R.V.P., A.v.A., J.T., P.C., N.V., and C.S.; investigation, E. Kokkinou, T.S., R.V.P., A.v.A., J.T., P.C., and N.V.; writing – original draft, J.M., H.R., E. Kokkinou, T.S., and R.V.P.; writing – review & editing; P.C., E. Kvedaraite, N.V., M.L., C.S., W.W., A.C., C.A.T., H.S., U.L., C.N., M.L., M.I., M.S., J.-I.H., E.J.V., and Y.T.B.; funding acquisition, J.M. and A.v.A.; resources, E. Kvedaraite, U.L., C.N., M.L., M.I., M.S., J.-I.H., E.J.V., H.R., and J.M.; supervision, J.M.

## DECLARATION OF INTERESTS

The authors declare no competing interests.

Received: July 22, 2022
Revised: December 15, 2022
Accepted: April 14, 2023
Published: May 8, 2023

## REFERENCES

1. Rosen, M.J., Dhawan, A., and Saeed, S.A. (2015). Inflammatory bowel disease in children and adolescents. JAMA Pediatr. *169*, 1053–1060. https://doi.org/10.1001/JAMAPEDIATRICS.2015.1982.

2. Van Limbergen, J., Russell, R.K., Drummond, H.E., Aldhous, M.C., Round, N.K., Nimmo, E.R., Smith, L., Gillett, P.M., McGrogan, P., Weaver, L.T., et al. (2008). Definition of phenotypic characteristics of childhood-onset inflammatory bowel disease. Gastroenterology *135*, 1114–1122. https://doi.org/10.1053/J.GASTRO.2008.06.081.

3. Zhang, Y.Z., and Li, Y.Y. (2014). Inflammatory bowel disease: pathogenesis. World J. Gastroenterol. *20*, 91–99. https://doi.org/10.3748/WJG.V20.I1.91.

4. Powrie, F., Leach, M.W., Mauze, S., Caddle, L.B., and Coffman, R.L. (1993). Phenotypically distinct subsets of CD4+ T cells induce or protect from chronic intestinal inflammation in C. B-17 scid mice. Int. Immunol. *5*, 1461–1471. https://doi.org/10.1093/INTIMM/5.11.1461.

5. Mitsialis, V., Wall, S., Liu, P., Ordovas-Montanes, J., Parmet, T., Vukovic, M., Spencer, D., Field, M., McCourt, C., Toothaker, J., et al. (2020). Single-cell analyses of colon and blood reveal distinct immune cell signatures of ulcerative colitis and Crohn's disease. Gastroenterology *159*, 591–608.e10. https://doi.org/10.1053/J.GASTRO.2020.04.074.

6. Smids, C., Horjus Talabur Horje, C.S., Drylewicz, J., Roosenboom, B., Groenen, M.J.M., van Koolwijk, E., Wahab, P.J., van Lochem, E.G., and Wahab, P.J. (2018). Intestinal T cell profiling in inflammatory bowel disease: linking T cell subsets to disease activity and disease course. J. Crohns Colitis *12*, 465–475. https://doi.org/10.1093/ECCO-JCC/JJX160.

7. Maxwell, J.R., Zhang, Y., Brown, W.A., Smith, C.L., Byrne, F.R., Fiorino, M., Stevens, E., Bigler, J., Davis, J.A., Rottman, J.B., et al. (2015). Differential roles for interleukin-23 and interleukin-17 in intestinal immunoregulation. Immunity *43*, 739–750. https://doi.org/10.1016/J.IMMUNI.2015.08.019.

8. Trejdosiewicz, L.K., Calabrese, A., Smart, C.J., Oakes, D.J., Howdle, P.D., Crabtree, J.E., Losowsky, M.S., Lancaster, F., and Boylston, A.W. (1991). Gamma delta T cell receptor-positive cells of the human gastrointestinal mucosa: occurrence and V region gene expression in Heliobacter pylori-associated gastritis, coeliac disease and inflammatory bowel disease - PubMed. Clin. Exp. Immunol. *84*, 440–444.

9. Serriari, N.E., Eoche, M., Lamotte, L., Lion, J., Fumery, M., Marcelo, P., Chatelain, D., Barre, A., Nguyen-Khac, E., Lantz, O., et al. (2014). Innate mucosal-associated invariant T (MAIT) cells are activated in inflammatory bowel diseases. Clin. Exp. Immunol. *176*, 266–274. https://doi.org/10.1111/CEI.12277.

10. D'Amico, F., Peyrin-Biroulet, L., Danese, S., and Fiorino, G. (2020). New drugs in the pipeline for the treatment of inflammatory bowel diseases: what is coming? Curr. Opin. Pharmacol. *55*, 141–150. https://doi.org/10.1016/J.COPH.2020.10.015.

11. Colonna, M. (2018). Innate lymphoid cells: diversity, plasticity, and unique functions in immunity. Immunity *48*, 1104–1117. https://doi.org/10.1016/J.IMMUNI.2018.05.013.

12. Bernink, J.H., Peters, C.P., Munneke, M., Te Velde, A.A., Meijer, S.L., Weijer, K., Hreggvidsdottir, H.S., Heinsbroek, S.E., Legrand, N., Buskens, C.J., et al. (2013). Human type 1 innate lymphoid cells accumulate in inflamed mucosal tissues. Nat. Immunol. *14*, 221–229. https://doi.org/10.1038/NI.2534.

13. Forkel, M., VanTol, S., Höög, C., Michaëlsson, J., Almer, S., and Mjösberg, J. (2019). Distinct alterations in the composition of mucosal innate lymphoid cells in newly diagnosed and established Crohn's disease and ulcerative colitis. J. Crohns Colitis *13*, 67–78. https://doi.org/10.1093/ECCO-JCC/JJY119.

14. Geremia, A., Arancibia-Cárcamo, C.V., Fleming, M.P.P., Rust, N., Singh, B., Mortensen, N.J., Travis, S.P.L., and Powrie, F. (2011). IL-23-responsive innate lymphoid cells are increased in inflammatory bowel disease. J. Exp. Med. *208*, 1127–1133. https://doi.org/10.1084/JEM.20101712.

15. Martin, J.C., Chang, C., Boschetti, G., Ungaro, R., Giri, M., Grout, J.A., Gettler, K., Chuang, L.S., Nayar, S., Greenstein, A.J., et al. (2019). Single-cell analysis of Crohn's disease lesions identifies a pathogenic cellular module associated with resistance to anti-TNF therapy. Cell *178*, 1493–1508.e20. https://doi.org/10.1016/J.CELL.2019.08.008.

16. Uniken Venema, W.T., Voskuil, M.D., Vila, A.V., van der Vries, G., Jansen, B.H., Jabri, B., Faber, K.N., Dijkstra, G., Xavier, R.J., Wijmenga, C., et al. (2019). Single-cell RNA sequencing of blood and ileal T cells from patients with Crohn's disease reveals tissue-specific characteristics and drug targets. Gastroenterology *156*, 812–815.e22. https://doi.org/10.1053/J.GASTRO.2018.10.046.

17. Huang, B., Chen, Z., Geng, L., Wang, J., Liang, H., Cao, Y., Chen, H., Huang, W., Su, M., Wang, H., et al. (2019). Mucosal profiling of pediatric-onset colitis and IBD reveals common pathogenics and therapeutic pathways. Cell *179*, 1160–1176.e24. https://doi.org/10.1016/J.CELL.2019.10.027.

18. Corridoni, D., Antanaviciute, A., Gupta, T., Fawkner-Corbett, D., Aulicino, A., Jagielowicz, M., Parikh, K., Repapi, E., Taylor, S., Ishikawa, D., et al. (2020). Single-cell atlas of colonic CD8+ T cells in ulcerative colitis. Nat. Med. *26*, 1480–1490. https://doi.org/10.1038/s41591-020-1003-4.

19. Jaeger, N., Gamini, R., Cella, M., Schettini, J.L., Bugatti, M., Zhao, S., Rosadini, C.V., Esaulova, E., Di Luccia, B., Kinnett, B., et al. (2021). Single-cell analyses of Crohn's disease tissues reveal intestinal intraepithelial T cells heterogeneity and altered subset distributions. Nat. Commun. *121*, 1921–2012. https://doi.org/10.1038/s41467-021-22164-6.

20. Ludvigsson, J.F., Andersson, M., Bengtsson, J., Eberhardson, M., Fagerberg, U.L., Grip, O., Halfvarson, J., Hjortswang, H., Jäghult, S., Karling, P., et al. (2019). Swedish inflammatory bowel disease register (SWIBREG) - a nationwide quality register. Scand. J. Gastroenterol. *54*, 1089–1101. https://doi.org/10.1080/00365521.2019.1660799.

21. D'Haens, G.R., Geboes, K., Peeters, M., Baert, F., Penninckx, F., and Rutgeerts, P. (1998). Early lesions of recurrent Crohn's disease caused by infusion of intestinal contents in excluded ileum. Gastroenterology *114*, 262–267. https://doi.org/10.1016/S0016-5085(98)70476-7.

22. Yudanin, N.A., Schmitz, F., Flamar, A.L., Thome, J.J.C., Tait Wojno, E., Moeller, J.B., Schirmer, M., Latorre, I.J., Xavier, R.J., Farber, D.L., et al. (2019). Spatial and temporal mapping of human innate lymphoid cells reveals elements of tissue specificity. Immunity *50*, 505–519.e4. https://doi.org/10.1016/J.IMMUNI.2019.01.012.

23. Langfelder, P., and Horvath, S. (2008). WGCNA: an R package for weighted correlation network analysis. BMC Bioinf. *9*, 559–613. https://doi.org/10.1186/1471-2105-9-559/FIGURES/4.

24. Mazzurana, L., Czarnewski, P., Jonsson, V., Wigge, L., Ringnér, M., Williams, T.C., Ravindran, A., Björklund, Å.K., Säfholm, J., Nilsson, G., et al. (2021). Tissue-specific transcriptional imprinting and heterogeneity in human innate lymphoid cells revealed by full-length single-cell RNA-sequencing. Cell Res. *31*, 554–568. https://doi.org/10.1038/S41422-020-00445-X.

25. Sadik, A., Somarribas Patterson, L.F., Öztürk, S., Mohapatra, S.R., Panitz, V., Secker, P.F., Pfänder, P., Loth, S., Salem, H., Prentzell, M.T., et al. (2020). IL4I1 is a metabolic immune checkpoint that activates the AHR and promotes tumor progression. Cell *182*, 1252–1270.e34. https://doi.org/10.1016/J.CELL.2020.07.038.

26. Santarlasci, V., Maggi, L., Mazzoni, A., Capone, M., Querci, V., Rossi, M.C., Beltrame, L., Cavalieri, D., De Palma, R., Liotta, F., et al. (2014). IL-4-induced gene 1 maintains high Tob1 expression that contributes to TCR unresponsiveness in human T helper 17 cells. Eur. J. Immunol. *44*, 654–661. https://doi.org/10.1002/EJI.201344047.

27. Lin, C.C., Bradstreet, T.R., Schwarzkopf, E.A., Sim, J., Carrero, J.A., Chou, C., Cook, L.E., Egawa, T., Taneja, R., Murphy, T.L., et al. (2014). Bhlhe40 controls cytokine production by T cells and is essential for pathogenicity in autoimmune neuroinflammation. Nat. Commun. *5*, 3551. https://doi.org/10.1038/NCOMMS4551.

28. Kanda, M., Yamanaka, H., Kojo, S., Usui, Y., Honda, H., Sotomaru, Y., Harada, M., Taniguchi, M., Suzuki, N., Atsumi, T., et al. (2016). Transcriptional regulator Bhlhe40 works as a cofactor of T-bet in the regulation of IFN-$\gamma$ production in iNKT cells. Proc. Natl. Acad. Sci. USA *113*, E3394–E3402. https://doi.org/10.1073/PNAS.1604178113.

29. Rao, A., Strauss, O., Kokkinou, E., Bruchard, M., Tripathi, K.P., Schlums, H., Carrasco, A., Mazzurana, L., Konya, V., Villablanca, E.J., et al. (2020). Cytokines regulate the antigen-presenting characteristics of human circulating and tissue-resident intestinal ILCs. Nat. Commun. *11*, 2049. https://doi.org/10.1038/S41467-020-15695-X.

30. Hepworth, M.R., Fung, T.C., Masur, S.H., Kelsen, J.R., McConnell, F.M., Dubrot, J., Withers, D.R., Hugues, S., Farrar, M.A., Reith, W., et al. (2015). Group 3 innate lymphoid cells mediate intestinal selection of commensal bacteria-specific CD4+ T cells. Science *348*, 1031–1035. https://doi.org/10.1126/SCIENCE.AAA4812.

31. Lehmann, F.M., von Burg, N., Ivanek, R., Teufel, C., Horvath, E., Peter, A., Turchinovich, G., Staehli, D., Eichlisberger, T., Gomez de Agüero, M., et al. (2020). Microbiota-induced tissue signals regulate ILC3-mediated antigen presentation. Nat. Commun. *11*, 1794. https://doi.org/10.1038/S41467-020-15612-2.

32. Von Burg, N., Chappaz, S., Baerenwaldt, A., Horvath, E., Bose Dasgupta, S., Ashok, D., Pieters, J., Tacchini-Cottier, F., Rolink, A., Acha-Orbea, H., and Finke, D. (2014). Activated group 3 innate lymphoid cells promote T-cell-mediated immune responses. Proc. Natl. Acad. Sci. USA *111*, 12835–12840. https://doi.org/10.1073/PNAS.1406908111/SUPPL_FILE/PNAS.201406908SI.PDF.

33. Bielecki, P., Riesenfeld, S.J., Hütter, J.C., Torlai Triglia, E., Kowalczyk, M.S., Ricardo-Gonzalez, R.R., Lian, M., Amezcua Vesely, M.C., Kroehling, L., Xu, H., et al. (2021). Skin-resident innate lymphoid cells converge on a pathogenic effector state. Nature *592*, 128–132. https://doi.org/10.1038/S41586-021-03188-W.

34. Kokkinou, E., Pandey, R.V., Mazzurana, L., Gutierrez-Perez, I., Tibbitt, C.A., Weigel, W., Soini, T., Carrasco, A., Rao, A., Nagasawa, M., et al. (2022). CD45RA+CD62L− ILCs in human tissues represent a quiescent local reservoir for the generation of differentiated ILCs. Sci. Immunol. *7*, eabj8301. https://doi.org/10.1126/SCIIMMUNOL.ABJ8301.

35. Mitsuishi, T. (2019). Correlation between histological findings and endoscopic findings in patients with ulcerative colitis: Basal plasmacytosis is an important finding suggesting active inflammation. JGH Open *3*, 100–104. https://doi.org/10.1002/JGH3.12111.

36. Geboes, K., Riddell, R., Öst, A., Jensfelt, B., Persson, T., and Löfberg, R. (2000). A reproducible grading scale for histological assessment of inflammation in ulcerative colitis. Gut *47*, 404–409. https://doi.org/10.1136/GUT.47.3.404.

37. Hart, G.T., Tran, T.M., Theorell, J., Schlums, H., Arora, G., Rajagopalan, S., Sangala, A.D.J., Welsh, K.J., Traore, B., Pierce, S.K., et al. (2019). Adaptive NK cells in people exposed to Plasmodium falciparum correlate with protection from malaria. J. Exp. Med. *216*, 1280–1290. https://doi.org/10.1084/JEM.20181681.

38. Zhao, J., Jaffe, A., Li, H., Lindenbaum, O., Sefik, E., Jackson, R., Cheng, X., Flavell, R.A., and Kluger, Y. (2021). Detection of differentially abundant cell subpopulations in scrna-seq data. Proc. Natl. Acad. Sci. USA *118*. https://doi.org/10.1073/PNAS.2100293118.

39. Huhn, O., Ivarsson, M.A., Gardner, L., Hollinshead, M., Stinchcombe, J.C., Chen, P., Shreeve, N., Chazara, O., Farrell, L.E., Theorell, J., et al. (2020). Distinctive phenotypes and functions of innate lymphoid cells in human decidua during early pregnancy. Nat. Commun. *11*, 381. https://doi.org/10.1038/S41467-019-14123-Z.

40. Breloer, M., and Fleischer, B. (2008). CD83 regulates lymphocyte maturation, activation and homeostasis. Trends Immunol. *29*, 186–194. https://doi.org/10.1016/J.IT.2008.01.009.

41. Schoepfer, A., Santos, J., Fournier, N., Schibli, S., Spalinger, J., Vavricka, S., Safroneeva, E., Aslan, N., Rogler, G., Braegger, C., and Nydegger, A. (2019). Systematic analysis of the impact of diagnostic delay on bowel damage in paediatric versus adult onset Crohn's disease. J. Crohns Colitis *13*, 1334–1342. https://doi.org/10.1093/ECCO-JCC/JJZ065.

42. Fuchs, A., Vermi, W., Lee, J.S., Lonardi, S., Gilfillan, S., Newberry, R.D., Cella, M., and Colonna, M. (2013). Intraepithelial type 1 innate lymphoid cells are a unique subset of cytokine responsive interferon-γ-producing cells. Immunity *38*, 769–781. https://doi.org/10.1016/J.IMMUNI.2013.02.010.

43. Hoorweg, K., Peters, C.P., Cornelissen, F., Aparicio-Domingo, P., Papazian, N., Kazemier, G., Mjösberg, J.M., Spits, H., and Cupedo, T. (2012). Functional differences between human NKp44- and NKp44+ RORC+ innate lymphoid cells. Front. Immunol. *3*, 72. https://doi.org/10.3389/FIMMU.2012.00072/BIBTEX.

44. Sonnenberg, G.F., Monticelli, L.A., Elloso, M.M., Fouser, L.A., and Artis, D. (2011). CD4(+) lymphoid tissue-inducer cells promote innate immunity in the gut. Immunity *34*, 122–134. https://doi.org/10.1016/J.IMMUNI.2010.12.009.

45. Lim, A.I., Li, Y., Lopez-Lastra, S., Stadhouders, R., Paul, F., Casrouge, A., Serafini, N., Puel, A., Bustamante, J., Surace, L., et al. (2017). Systemic human ILC precursors provide a substrate for tissue ILC differentiation. Cell *168*, 1086–1100.e10. https://doi.org/10.1016/j.cell.2017.02.021.

46. Lee, J.S., Cella, M., McDonald, K.G., Garlanda, C., Kennedy, G.D., Nukaya, M., Mantovani, A., Kopan, R., Bradfield, C.A., Newberry, R.D., and Colonna, M. (2011). AHR drives the development of gut ILC22 cells and postnatal lymphoid tissues via pathways dependent on and independent of Notch. Nat. Immunol. *13*, 144–151. https://doi.org/10.1038/NI.2187.

47. Klose, C.S.N., and Artis, D. (2016). Innate lymphoid cells as regulators of immunity, inflammation and tissue homeostasis. Nat. Immunol. *177*, 765–774. https://doi.org/10.1038/ni.3489.

48. Mjösberg, J.M., Trifari, S., Crellin, N.K., Peters, C.P., Van Drunen, C.M., Piet, B., Fokkens, W.J., Cupedo, T., and Spits, H. (2011). Human IL-25- and IL-33-responsive type 2 innate lymphoid cells are defined by expression of CRTH2 and CD161. Nat. Immunol. *12*, 1055–1062. https://doi.org/10.1038/ni.2104.

49. Simoni, Y., Fehlings, M., Kløverpris, H.N., McGovern, N., Koo, S.L., Loh, C.Y., Lim, S., Kurioka, A., Fergusson, J.R., Tang, C.L., et al. (2017). Human innate lymphoid cell subsets possess tissue-type based heterogeneity in phenotype and frequency. Immunity *46*, 148–161. https://doi.org/10.1016/J.IMMUNI.2016.11.005/ATTACHMENT/38FF1BFA-4E9D-4421-A0AC-4B3B45F9B9AA/MMC1.PDF.

50. Kuwahara, M., Yamashita, M., Shinoda, K., Tofukuji, S., Onodera, A., Shinnakasu, R., Motohashi, S., Hosokawa, H., Tumes, D., Iwamura, C., et al. (2012). The transcription factor Sox4 is a downstream target of signaling by the cytokine TGF-β and suppresses TH2 differentiation. Nat. Immunol. *13*, 778–786. https://doi.org/10.1038/NI.2362.

51. Hughes, T., Briercheck, E.L., Freud, A.G., Trotta, R., McClory, S., Scoville, S.D., Keller, K., Deng, Y., Cole, J., Harrison, N., et al. (2014). AHR prevents human IL-1R1hi ILC3 differentiation to natural killer cells. Cell Rep. *8*, 150–162. https://doi.org/10.1016/J.CELREP.2014.05.042.

52. Welte, S., Kuttruff, S., Waldhauer, I., and Steinle, A. (2006). Mutual activation of natural killer cells and monocytes mediated by NKp80-AICL interaction. Nat. Immunol. *7*, 1334–1342. https://doi.org/10.1038/NI1402.

53. Tewary, P., Yang, D., De La Rosa, G., Li, Y., Finn, M.W., Krensky, A.M., Clayberger, C., and Oppenheim, J.J. (2010). Granulysin activates antigen-presenting cells through TLR4 and acts as an immune alarmin. Blood *116*, 3465–3474. https://doi.org/10.1182/BLOOD-2010-03-273953.

54. Krabbendam, L., Heesters, B.A., Kradolfer, C.M.A., Haverkate, N.J.E., Becker, M.A.J., Buskens, C.J., Bemelman, W.A., Bernink, J.H., and Spits, H. (2021). CD127+ CD94+ innate lymphoid cells expressing granulysin and perforin are expanded in patients with Crohn's disease. Nat. Commun. *12*, 5841. https://doi.org/10.1038/S41467-021-26187-X.

55. Allez, M., Tieng, V., Nakazawa, A., Treton, X., Pacault, V., Dulphy, N., Caillat-Zucman, S., Paul, P., Gornet, J.M., Douay, C., et al. (2007). CD4+NKG2D+ T cells in Crohn's disease mediate inflammatory and cytotoxic responses through MICA interactions. Gastroenterology *132*, 2346–2358. https://doi.org/10.1053/J.GASTRO.2007.03.025.

56. Chen, L., He, Z., Reis, B.S., Gelles, J.D., Chipuk, J.E., Ting, A.T., Spicer, J.A., Trapani, J.A., Furtado, G.C., and Lira, S.A. (2022). IFN-γ + cytotoxic CD4 + T lymphocytes are involved in the pathogenesis of colitis induced by IL-23 and the food colorant Red 40. Cell. Mol. Immunol. *19*, 777–790. https://doi.org/10.1038/S41423-022-00864-3.

57. Ma, J., Tibbitt, C.A., Georen, S.K., Christian, M., Murrell, B., Cardell, L.O., Bachert, C., and Coquet, J.M. (2021). Single-cell analysis pinpoints distinct populations of cytotoxic CD4+T cells and an IL-10+CD109+TH2 cell population in nasal polyps. Sci. Immunol. *6*, 6356. https://doi.org/10.

1126/SCIIMMUNOL.ABG6356/SUPPL_FILE/SCIIMMUNOL.ABG6356_-TABLES_S1_TO_S7.ZIP.

58. Mogilenko, D.A., Shpynov, O., Andhey, P.S., Arthur, L., Swain, A., Esaulova, E., Brioschi, S., Shchukina, I., Kerndl, M., Bambouskova, M., et al. (2021). Comprehensive profiling of an aging immune system reveals clonal GZMK + CD8 + T cells as conserved hallmark of inflammaging. Immunity *54*, 99–115.e12. https://doi.org/10.1016/J.IMMUNI.2020.11.005.

59. Perez, R.K., Gordon, M.G., Subramaniam, M., Kim, M.C., Hartoularos, G.C., Targ, S., Sun, Y., Ogorodnikov, A., Bueno, R., Lu, A., et al. (2022). Single-cell RNA-seq reveals cell type-specific molecular and genetic associations to lupus. Science *376*, eabf1970. https://doi.org/10.1126/SCIENCE.ABF1970.

60. Butler, A., Hoffman, P., Smibert, P., Papalexi, E., and Satija, R. (2018). Integrating single-cell transcriptomic data across different conditions, technologies, and species. Nat. Biotechnol. *36*, 411–420. https://doi.org/10.1038/NBT.4096.

61. Korsunsky, I., Millard, N., Fan, J., Slowikowski, K., Zhang, F., Wei, K., Baglaenko, Y., Brenner, M., Loh, P.R., and Raychaudhuri, S. (2019). Fast, sensitive and accurate integration of single-cell data with Harmony. Nat. Methods *16*, 1289–1296. https://doi.org/10.1038/S41592-019-0619-0.

62. Leek, J.T., Johnson, W.E., Parker, H.S., Jaffe, A.E., and Storey, J.D. (2012). The sva package for removing batch effects and other unwanted variation in high-throughput experiments. Bioinformatics *28*, 882–883. https://doi.org/10.1093/BIOINFORMATICS/BTS034.

63. Street, K., Risso, D., Fletcher, R.B., Das, D., Ngai, J., Yosef, N., Purdom, E., and Dudoit, S. (2018). Slingshot: cell lineage and pseudotime inference for single-cell transcriptomics. BMC Genom. *19*, 477. https://doi.org/10.1186/S12864-018-4772-0.

64. Amezquita, R.A., Lun, A.T.L., Becht, E., Carey, V.J., Carpp, L.N., Geistlinger, L., Marini, F., Rue-Albrecht, K., Risso, D., Soneson, C., et al. (2019). Orchestrating single-cell analysis with Bioconductor. Nat. Methods *172*, 137–145. https://doi.org/10.1038/s41592-019-0654-x.

65. Theorell, A., Bryceson, Y.T., and Theorell, J. (2019). Determination of essential phenotypic elements of clusters in high-dimensional entities—depeche. PLoS One *14*, e0203247. https://doi.org/10.1371/JOURNAL.PONE.0203247.

66. Mazzurana, L., Forkel, M., Rao, A., Van Acker, A., Kokkinou, E., Ichiya, T., Almer, S., Höög, C., Friberg, D., and Mjösberg, J. (2019). Suppression of Aiolos and Ikaros expression by lenalidomide reduces human ILC3-ILC1/NK cell transdifferentiation. Eur. J. Immunol. *49*, 1344–1355. https://doi.org/10.1002/EJI.201848075.

## STAR★METHODS

### KEY RESOURCES TABLE

| REAGENT or RESOURCE | SOURCE | IDENTIFIER |
|---|---|---|
| Antibodies | | |
| anti-human CD1a FITC (Clone HI149) | Biolegend | Cat#300104 |
| anti-human CD14 FITC (Clone Tuk4) | LifeTech | Cat#MHCD1401-4 |
| anti-human CD19 FITC (Clone 4G7) | BD | Cat#345776 |
| anti-human CD123 FITC (Clone 6H6) | Biolegend | Cat#306014 |
| anti-human BDCA2 FITC (Clone AC144) | Miltenyi | Cat#130-113-192 |
| anti-human FcER1a FITC (Clone AER-27) | Biolegend | Cat#334608 |
| anti-human CD34 FITC (Clone 581) | Biolegend | Cat#343504 |
| anti-human CD94 FITC (Clone DX22) | Biolegend | Cat#305504 |
| anti-human TCRab FITC (Clone IP26) | Biolegend | Cat#306706 |
| anti-human TCRgd FITC (Clone B1) | Biolegend | Cat#331208 |
| anti-human Eomes FITC (Clone WD1928) | eBioscience | Cat#11-4877-42 |
| anti-human NKG2A (CD159a) APC (Clone Z199) | Beckman Coulter | Cat#60797 |
| anti-human Eomes efluor660 (Clone WD1928) | eBioscience | Cat#50-4877-41 |
| anti-human CD69 AF700 (Clone FN50) | Biolegend | Cat#310992 |
| anti-human CD3 AF700 (Clone UCHT1) | BD | Cat#557943 |
| anti-human CD62L APC-Cy7 (Clone DREG-56) | Biolegend | Cat#304814 |
| anti-human CRTH2 V450 (Clone BM16) | BD | Cat#561661 |
| anti-human CD45 V500 (Clone HI30) | BD | Cat#560777 |
| anti-human CD1a biotin (Clone HI149) | Biolegend | Cat#300112 |
| anti-human CD14 biotin (Clone HCD14) | Biolegend | Cat#325624 |
| anti-human CD19 biotin (Clone HIB19) | Biolegend | Cat#302204 |
| anti-human CD123 biotin (Clone 6H6) | Biolegend | Cat#306004 |
| anti-human BDCA2 biotin (Clone GoH3) | Miltenyi | Cat#130-097-923 |
| anti-human FcER1a biotin (Clone AER-37) | eBioscience | Cat#13-5899-82 |
| anti-human CD34 biotin (Clone 581) | Biolegend | Cat#343524 |
| anti-human CD94 biotin (Clone REA113) | Miltenyi | Cat#130-098-967 |
| anti-human TCRab biotin (Clone IP26) | Biolegend | Cat#306704 |
| anti-human TCRgd biotin (Clone B1) | Biolegend | Cat#331206 |
| Streptavidin BV570 | Biolegend | Cat#405227 |
| anti-human CD90 BV605 (Clone 5E10) | Biolegend | Cat#328128 |
| anti-human CD161 BV605 (Clone HP-3G10) | Biolegend | Cat#339915 |
| anti-human CD45 BV650 (Clone HI30) | Biolegend | Cat#304043 |
| anti-human CD56 BV650 (Clone HCD56) | Biolegend | Cat#318343 |
| anti-human CD56 BV711 (Clone HCD56) | Biolegend | Cat#318336 |
| anti-human CD3 BV785 (Clone OKT3) | Biolegend | Cat#317329 |
| anti-human NKG2A (CD159a) PE (Clone 131411) | R&D | Cat#FAB1059P |
| anti-human ROR-gt PE (Clone AFKJS-9) | eBioscience | Cat#12-6988-82 |
| anti-human HLA-DR PE (Clone L243) | eBioscience | Cat#12-9952-41 |
| anti-human CRTH2 PE-CF594 (Clone BM16) | BD | Cat#563501 |
| anti-human CD161 PE-CF594 (Clone HP-3G10) | Biolegend | Cat#339939 |

(*Continued on next page*)

*Continued*

| REAGENT or RESOURCE | SOURCE | IDENTIFIER |
|---|---|---|
| anti-human T-bet PE-CF594 (Clone O4-46) | BD | Cat#562467 |
| anti-human NKp44 PE-Cy5 (Clone Z231) | Beckman Coulter | Cat#A66903 |
| anti-human CD117 PE-Cy5.5 (Clone 95C3) | Beckman Coulter | Cat#A66333 |
| anti-human CD127 PE-Cy7 (Clone R34.34) | Beckman Coulter | Cat#A64618 |
| anti-human CD45 BUV395 (Clone HI30) | BD | Cat#563791 |
| anti-human CD45RA BUV737 (Clone HI100) | BD | Cat#564442 |
| anti-human CD3 BV570 (Clone UTCH1) | Biolegend | Cat#300436 |
| anti-human CD69 BV650 (Clone FN50) | Biolegend | Cat#310934 |
| anti-human CD4 BUV496 (Clone SK3) | BD | Cat#564652 |
| anti-human CD8 BUV805 (Clone SK1) | BD | Cat#612889 |
| Biological samples | | |
| Intestinal biopsies from pediatric IBD and non-IBD patients | Karolinska University Hospital or Sachs' Children and Youth Hospital | N/A |
| Intestinal specimens from adult IBD and non-IBD patients | Karolinska University Hospital | N/A |
| Peripheral blood from pediatric IBD patients | Karolinska University Hospital | N/A |
| Deposited data | | |
| Count matrix from cell ranger | This paper | GEO Accession Number: GSE169136 |
| R scripts | This paper | https://github.com/ramvinay/Kokkinou_pIBD https://github.com/jtheorell/Kokkinou_gut_neighbors/ |
| Software and algorithms | | |
| R v.4.1.0 | R Core Team | https://cran.r-project.org/ |
| Cell Ranger v.2.1.1 | 10x Genomics | https://support.10xgenomics.com/single-cell-gene-expression/software |
| Seurat v.4.1.1 | Butler et al., 2019[60] | https://github.com/satijalab/seurat/ |
| Harmony v.0.1 | Korsunsky et al., 2019[61] | https://github.com/immunogenomics/harmony |
| sva v.3.32.1 | Leek et al., 2022[62] | https://bioconductor.org/packages/release/bioc/html/sva.html |
| Slingshot v.2.2.1 | Street et al., 2018[63] | https://bioconductor.org/packages/release/bioc/html/slingshot.html |
| WGCNA v.1.70.3 | Langfelder et al., 2008[23] | https://horvath.genetics.ucla.edu/html/CoexpressionNetwork/Rpackages/WGCNA/ |
| SingleCellExperiment | Amezquita et al., 2020[64] | https://www.bioconductor.org/packages/release/bioc/html/SingleCellExperiment.html |
| DASeq v1.0.0 | Zhao et al., 2021[38] | https://github.com/KlugerLab/DAseq |
| DepecheR v.1.14.0 | Theorell et al., 2019[65] | https://www.bioconductor.org/packages/release/bioc/html/DepecheR.html |

(*Continued on next page*)

*Continued*

| REAGENT or RESOURCE | SOURCE | IDENTIFIER |
|---|---|---|
| Other | | |
| LIVE/DEAD™ Fixable Near-IR Dead Cell Stain Kit | Invitrogen | Cat#L34975 |
| LIVE/DEAD™ Fixable Aqua Dead Cell Stain Kit | Invitrogen | Cat#L34957 |
| LIVE/DEAD™ Fixable Green Dead Cell Stain Kit | Invitrogen | Cat#L34970 |
| Collagenase II | Sigma-Aldrich | Cat#C6885 |
| DNAse | Sigma-Aldrich | Cat#10104159001 |
| IMDM | Gibco | Cat#12440-053 |
| Foxp3/Transcription Factor Staining Buffer Set | eBioscience | Cat#00-5523-00 |
| Chromium™ Single Cell 3' Library & Gel Bead Kit v2, 16 rxns | 10x Genomics | Cat#PN-120237 |

## RESOURCE AVAILABILITY

### Lead contact

Further information and requests for resources should be directed to and will be fulfilled by the lead contact, Jenny Mjösberg (jenny.mjosberg@ki.se).

### Materials availability

The study did not generate new reagents.

### Data and code availability

- ScRNA-seq data (count matrix) are available at GEO under the accession number GSE169136.
- All codes (R scripts) have been deposited at: https://github.com/ramvinay/Kokkinou_pIBD and https://github.com/jtheorell/Kokkinou_gut_neighbors/
- Software and packages used for this analysis are listed in the key resources table.
- Any additional information required to reanalyze the data reported in this paper is available from the lead contact upon request.

## EXPERIMENTAL MODEL AND SUBJECT DETAILS

### Human patients and study design

Pediatric patients at the Karolinska University Hospital or Sachs' Children and Youth Hospital were included. All patients were treatment-naive and included upon diagnostic colonoscopy. IBD colitis diagnosis was endoscopically and histologically verified.

For flow cytometry analysis, 16 biopsies were obtained from two areas in the colon of pediatric patients with IBD (n = 19): the endoscopically most affected area (inflamed) and the endoscopically least-affected area (non-inflamed) (Table S1). Some patients had pancolitis or restricted ileal or upper GI-tract involvement, and thus, samples were taken only from the endoscopically inflamed or non-inflamed colonic mucosa, respectively. Non-IBD controls (n = 10), displayed unspecific gastrointestinal symptoms and colonoscopy was performed to rule out IBD diagnosis. All non-IBD controls had endoscopically and histologically normal gut mucosal findings with no signs of active or chronic inflammation.

For the scRNA-seq, treatment-naïve pediatric patients with CD-colitis were included (n = 6). Biopsies were sampled from endoscopically most inflamed and adjacent least inflamed areas of the colon and histologically evaluated (Table S3).

Colonic intestinal specimens from adult patients (Table S5), include non-IBD controls (non-affected intestine from colorectal cancer patients) (n = 8) as well as IBD patients that underwent surgery (n = 8, 2 UC, 6 CD).

### Ethical approval

This study was approved by the Swedish Ethical Review Authority (approval numbers 2010/32-31/4, 2018/323-31/1 and 2020-00507). Written informed consents were obtained from all patients and their caregivers.

### Characterization and inflammation activity scoring of patients with pIBD

Disease activity was evaluated with endoscopic and histopathological scorings. To evaluate the disease activity endoscopically, we used the endoscopic part of SWIBREG (Swedish Inflammatory Bowel Disease Register) scoring system (Table S2).[20] For pediatric

CD patients, SES-CD endoscopic score and Paris phenotypic classification were additionally assessed to characterize the patient group. For histopathological scoring, we used a modification of colonic global histologic disease activity score of diagnostic samples collected in close proximity to the research biopsies.[21] The evaluation was based on the written statement of an IBD-experienced pathologist. Established IBD biomarkers (shown in Tables S1 and S5), were taken as routine diagnostic examination: blood 0-36 (median 3) and fecal samples 0-60 (median 18) days prior to biopsy sampling and are listed for characterization of the patient groups.

## METHOD DETAILS

### Cell isolation from intestinal samples and blood

Cell isolation from colonic biopsies was performed as described in Mazzurana *et al* 2019, where the detailed protocols are provided.[66] Briefly, colonic biopsies were collected in wash buffer containing HBSS supplemented with antibiotics. For subsequent enzymatic digestion, wash buffer was removed and replaced with 1mL IMDM (supplemented with antibiotics), collagenase II 0.25 mg/mL; Sigma) and DNase (0.25 mg/mL; Roche). Magnetic stirring was performed at 37°C, 450rpm.

For 3 pIBD patients (Table S1), peripheral blood mononuclear cells (PBMC) were isolated as previously described[29,66] using Lymphoprep™ (Stemcell Technologies) with gradient density centrifugation at 2000 rpm for 20 min, room temperature (RT). Cells were collected, washed, counted and subjected to cell staining.

### Flow cytometry and cell sorting

For flow cytometry, colon cells or PBMC were stained with dead cell marker (DCM) and antibodies targeting surface antigens for 20-30 min at RT, followed by washing with FACS buffer (PBS containing 1% FCS). The cells were fixed with either 2% PFA for 10 min at RT, or in case of intracellular staining, with Foxp3 Fix/Perm solution kit (eBioscience) for 30 minutes at RT. For the latter, cells were subsequently washed with permeabilization buffer (eBioscience) and stained with antibodies targeting intracellular antigens for 30 min at RT. Samples were immediately acquired on a BD LSR Fortessa or BD Symphony A5 flow cytometer. Analysis of flow cytometry data was performed using FlowJo versions 9.9.6, 10.4.2 and 10.6.2 (TreeStar). A detailed list of the antibodies are shown in the key resources table.

For cell sorting, colonic ILCs, T cells and NK cells were surface-stained with lineage specific antibodies (all antibodies are indicated in the key resources table). Cells were sort-purified in cold RMPI using the BD FACSAria Fusion™. Gating strategy for sorted ILCs, T cells and NK cells is shown in Figure S1A. After sorting cells were immediately subjected to RNA library preparation.

### 10x genomics single-cell RNA sequencing

Chromium Single-Cell v2 3' Reagents Kit (10x Genomics) was used to generate single-cell RNA Sequencing libraries. In brief, cell suspension was spun down and resuspended in 17.4µl PBS with 0.04% BSA at a final concentration of 1000 cells/µl (target recovery of 10 000 cells). Accordingly, Master Mix was prepared at the adjusted volume of 16.4µl Nuclease-Free water. Cells were loaded on the Chromium Controller Single Cell A Chip to generate Gel Bead Emulsions (GEMs). GEMs were proceeded to GEM-RT incubation, cDNA amplification and library construction following the Chromium™ Single Cell 3′ library Kit v2 User Guide (10x Genomics). Quality controls for cDNA amplification and final barcoded libraries were performed using High Sensitivity DNA Assays from 2100 Agilent Bioanalyzer to assess the quantity and fragment size. Sequencing libraries were loaded at 2.1pM loading concentration on an Hi-Seq4000 with custom sequencing metrics (single-indexed sequencing run, 28/8/0/98 cycles for R1/i7/i5/R2) (Illumina, San Diego, CA).

## QUANTIFICATION AND STATISTICAL ANALYSIS

### Single-cell RNA sequencing data analysis

10x Cell Ranger toolkit (v2.1.1) was used for filtering and aligning reads onto human genome hg19 and counting barcodes and unique molecular identifiers (UMI). Seurat (v4.1.1)[60] was used for downstream 10x scRNA-seq data analysis. Briefly, cells with a percentage of mitochondria genes > 5% were considered as dead cells and were removed. Cells with < 200 genes and > 2500 detected genes were excluded. Genes detected in < 3 cells were excluded. Raw UMI counts were scaled and normalized through log transformation. We used Harmony for performing the integration of samples.[61] The top 4000 variable genes were selected for PCA and the first 40 dimensions were selected for UMAP and clustering analysis with resolutions 0.6. Seurat function FindAllMarkers (logFC threshold = 0.25) were used to find cluster- and sample-specific marker genes. Further, UMAP clusters were annotated by well-known marker genes and marker genes were detected by the *FindAllMarker*s function. The functions *FeaturePlot*, *VlnPlot* and *DotPlot* were used for feature plots, violins plots, heatmaps and dot plots, respectively, with default parameters. For subcluster analysis, we used the ComBat function from sva (v3.32.1)[62] R package to remove known batches (donors). Wilcoxon test was used for the DE analysis. Trajectory analysis on ILCs was performed using the Slingshot version 2.2.1[63] R package with Seurat object. We used UMAP coordinates and ILC subclusters 4 and 1 as the starting point (root).

### Weighted gene co-expression network analysis (WGCNA)

We used the weighted co-expression network analysis (WGCNA)[23] algorithm to identify gene co-expression networks. Specifically, 4000 variable genes were used for calculation of gene-to-gene Pearson correlations. The adjacency matrix was directly used for creation of the topological overlap matrix (TOM). Gene module identification was done with agglomerative hierarchical clustering on the TOM using Ward's squared linkage (Ward.D2) with a fixed number of 50 modules. Gene counts belonging to the same module were normalized between 0–1 individually and then averaged per cell for calculation of each gene module expression pattern. Modules were prioritized by fitting a general linear model (GLM) using each gene module expression pattern as the effect and the sample metadata (Louvain cluster, Donor) as the explaining variables. Each module and metadata parameter was tested independently. The output of the GLM fit results in the residual metric of "deviance" ($dev_{model}$) after fitting the model and "null deviance" ($dev_{null}$) when considering a NULL model. The percent difference in these two deviances ($\Delta dev$) was used as a measure of how much a particular metadata factor explains the expression pattern of a gene module:

$$\Delta dev = (dev_{null} - dev_{model}) / dev_{null}$$

This way, modules with higher$\Delta dev$were prioritized for further exploration.

### Euclidean neighbor-based smoothing of histology inflammation score

Since samples that are endoscopically scored as non-inflamed or inflamed are composed of samples with variable histological scores we needed a method to better define "inflammatory" and "non-inflammatory" cells to potentially discover genes involved in pIBD inflammation. This method builds on the concept of Euclidean neighbor-based smoothing, which has previously been used both in cytometry and scRNAseq analysis.[37,38] Its core assumption is that Euclidean neighbors in the transcriptomic space have similar functions.

The processing of the data was done using the neighSmooth function in the BioConductor package DepecheR, in a similar fashion as in Hart *et al*[37]. To even out differences in cell numbers between donors and conditions, the samples were divided into least inflamed (histo score 0-4), intermediate (histo score 7) and most inflamed (histo score 9-12). Then, random subsets of cells were chosen, so that each sample within these overarching inflammation groups had the same number of cells, and simultaneously that the least, intermediate, and most inflamed sample groups had the same number of total cells. In the end, a total number of 22065 neighbor cells were identified, ranging between 1461 and 3681 cells for each sample. After this, the 100 closest Euclidean neighbors were identified from this neighbor cell subset for all cells in the dataset, and each cell was given the average histo score for these 100 nearest neighbors. Each cell was further assessed for the number of samples represented among its 100 nearest neighbors. An arbitrary cutoff of five samples was used to filter out cell populations strongly overrepresented in outlier samples. For this more representative group of cells, aided by a histogram, cutoff values of $\leq 4$ for least inflamed cells and $\geq 9$ for most inflamed cells were introduced.

To confirm the results of the nearest neighbor-smoothing analysis of the histological scores, DAseq was used.[38] As this is a discriminant analysis tool, a few adaptions of the data was necessary. First, all samples with score 7 were excluded. Second, if both samples from one individual had low (<4) or high ($\geq$9) histological scores, the least extreme sample was removed. Subsequently, all remaining cells in the low or the high inflammation group were given these discriminant labels. This was followed by the standard DASeq pipeline. As a prediction threshold, the value of 0.5 was used.

### Statistical analyses

Statistical analysis and graphing of the flow cytometry data was performed using Prism 9 software (GraphPad Software, San Diego, US). The sample size for each experiment, the replicate number as well as the precision measures are included in the figure legends.

**Cell Reports Medicine, Volume *4***

## Supplemental information

# The single-cell transcriptional landscape of innate and adaptive lymphocytes in pediatric-onset colitis

**Efthymia Kokkinou, Tea Soini, Ram Vinay Pandey, Aline van Acker, Jakob Theorell, Paulo Czarnewski, Egle Kvedaraite, Niels Vandamme, Magda Lourda, Chiara Sorini, Whitney Weigel, Anna Carrasco, Christopher Andrew Tibbitt, Heinrich Schlums, Ulrik Lindforss, Caroline Nordenvall, Malin Ljunggren, Maja Ideström, Mattias Svensson, Jan-Inge Henter, Eduardo J. Villablanca, Yenan T. Bryceson, Helena Rolandsdotter, and Jenny Mjösberg**

## Supplemental information

# The single-cell transcriptional landscape of innate and adaptive lymphocytes in pediatric-onset colitis

**Efthymia Kokkinou, Tea Soini, Ram Vinay Pandey, Aline van Acker, Jakob Theorell, Paulo Czarnewski, Egle Kvedaraite, Niels Vandamme, Magda Lourda, Chiara Sorini, Whitney Weigel, Anna Carrasco, Christopher Andrew Tibbitt, Heinrich Schlums, Ulrik Lindforss, Caroline Nordenvall, Malin Ljunggren, Maja Ideström, Mattias Svensson, Jan-Inge Henter, Eduardo J. Villablanca, Yenan T. Bryceson, Helena Rolandsdotter, Jenny Mjösberg**

Figure S1

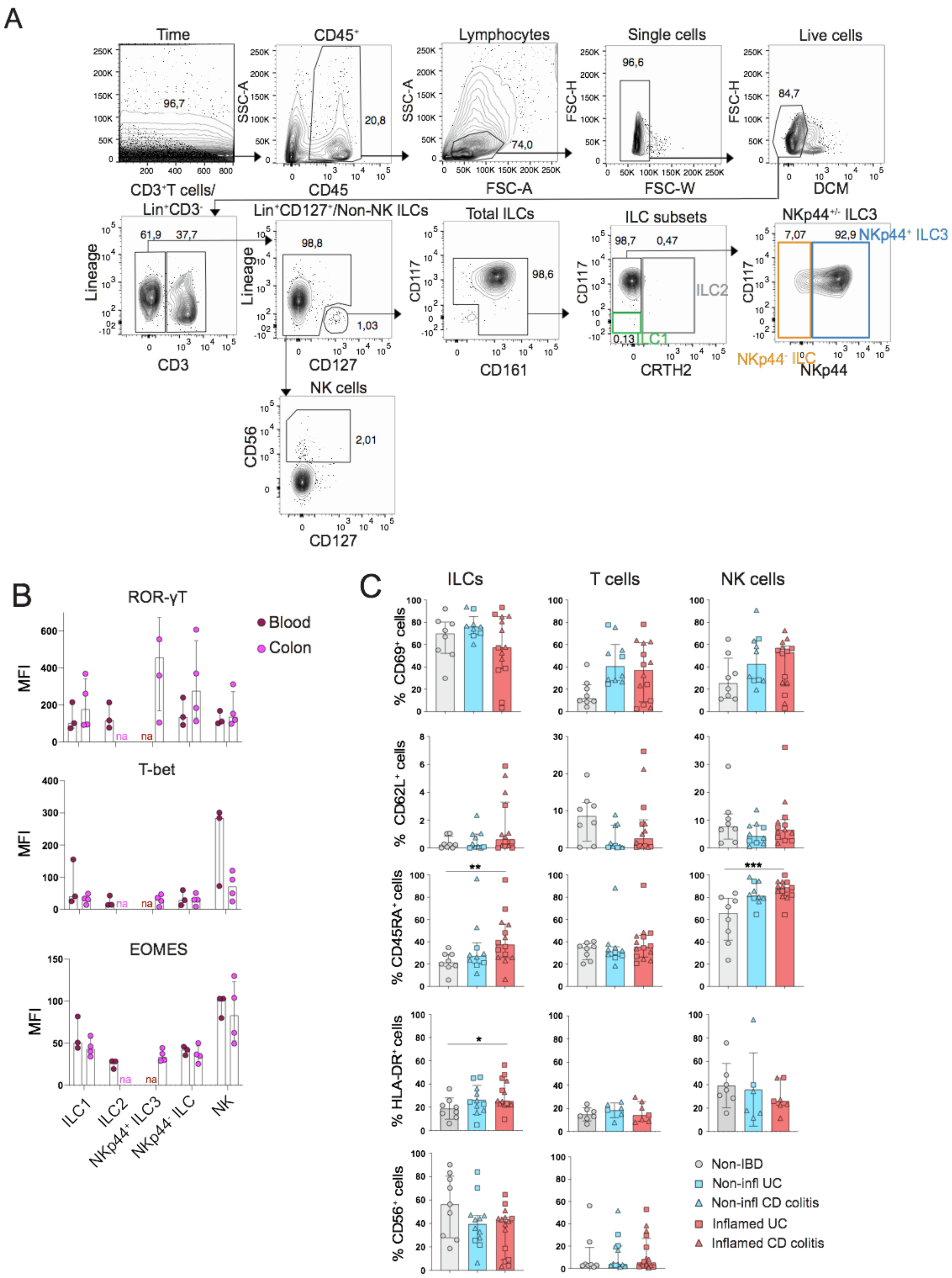

A
Time
CD45+
Lymphocytes
Single cells
Live cells
CD3+T cells/
Lin+CD3-
Lin+CD127+/Non-NK ILCs
Total ILCs
ILC subsets
NKp44+/- ILC3
NKp44+ ILC3
NKp44- ILC
ILC2
ILC1
NK cells
SSC-A
FSC-H
FSC-A
FSC-W
DCM
CD45
Lineage
CD3
CD127
CD117
CD161
CRTH2
NKp44
CD56
96,7
20,8
74,0
96,6
84,7
61,9
37,7
98,8
1,03
98,6
98,7
0,47
0,13
7,07
92,9
2,01
B
ROR-γT
T-bet
EOMES
MFI
Blood
Colon
na
ILC1
ILC2
NKp44+ ILC3
NKp44- ILC
NK
C
ILCs
T cells
NK cells
% CD69+ cells
% CD62L+ cells
% CD45RA+ cells
% HLA-DR+ cells
% CD56+ cells
Non-IBD
Non-infl UC
Non-infl CD colitis
Inflamed UC
Inflamed CD colitis

**Figure S1. Dysregulation of colonic ILC composition in pIBD colitis. Related to Figure 1. (A)** Gating strategy for the identification of T cells ($CD3^+$ $Lin^-$), ILCs ($CD3^-Lin^-CD127^+$ $CD161^+$) and NK cells ($CD3^-Lin^+$ $CD127^{-/dim}$ $CD56^+$) by flow cytometry in pediatric (p)IBD and non-IBD colon samples. **(B)** Bar graphs showing the geometric MFI expression of ROR-γT, T-bet and EOMES protein in blood (n = 3) and colon samples (n = 4) of pediatric non-IBD patients. **(C)** Bar graphs showing the frequencies of $CD69^+$, $CD62L^+$, $CD45RA^+$, HLA-$DR^+$ and $CD56^+$ cells out of ILCs, T cells and NK cells in non-IBD (n = 8-9), endoscopically non-inflamed (n = 6-12) and endoscopically inflamed (n = 6-14) pIBD colon samples (mean with SD). Statistical differences were detected with the Mann-Whitney test. Bar graphs are shown as Mean with SD. In (B) and (C) cells from each donor were analyzed in independent experiments.

# Figure S2

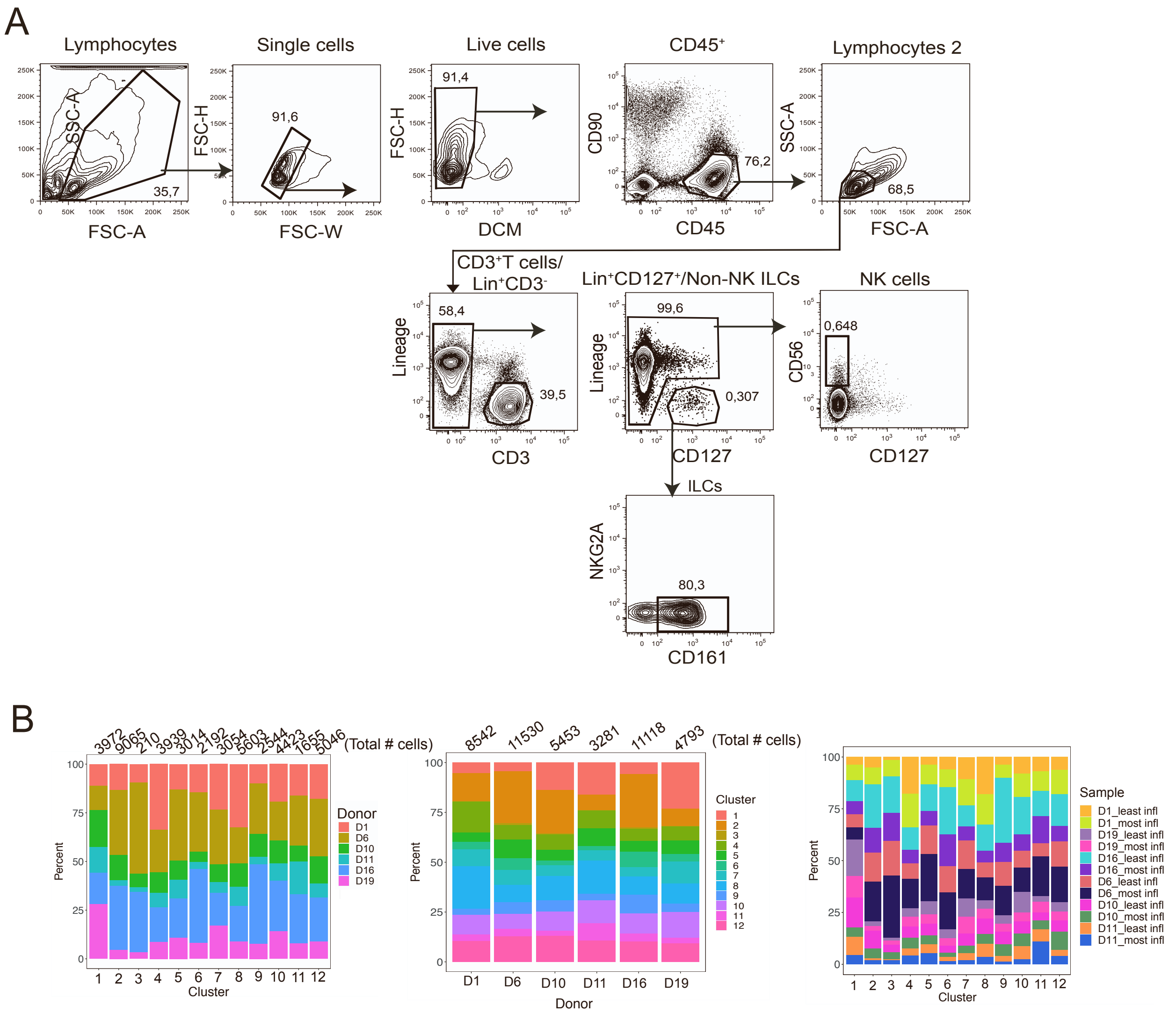


**Figure S2. Single-cell transcriptional profiling of colonic ILCs, NK- and T cells in pIBD colitis. Related to Figure 2. (A)** FACS-sorting gating strategy for T cells ($CD3^{+}$ $Lin^{-}$), ILCs ($CD3^{-}Lin^{-}CD127^{+}$ $CD161^{+}$) and NK cells ($CD3^{-}Lin^{+}$ $CD127^{-/dim}$ $CD56^{+}$) for scRNA-seq. **(B)** Stacked bar plot showing the contribution of each donor (n = 6) to the clusters shown in Figure 2B (left and middle). Stacked bar plot showing the contribution of each colon biopsy sample (n = 12) to the clusters shown in Figure 2B (right). Cells from each donor were analyzed in independent experiments.

Figure S3

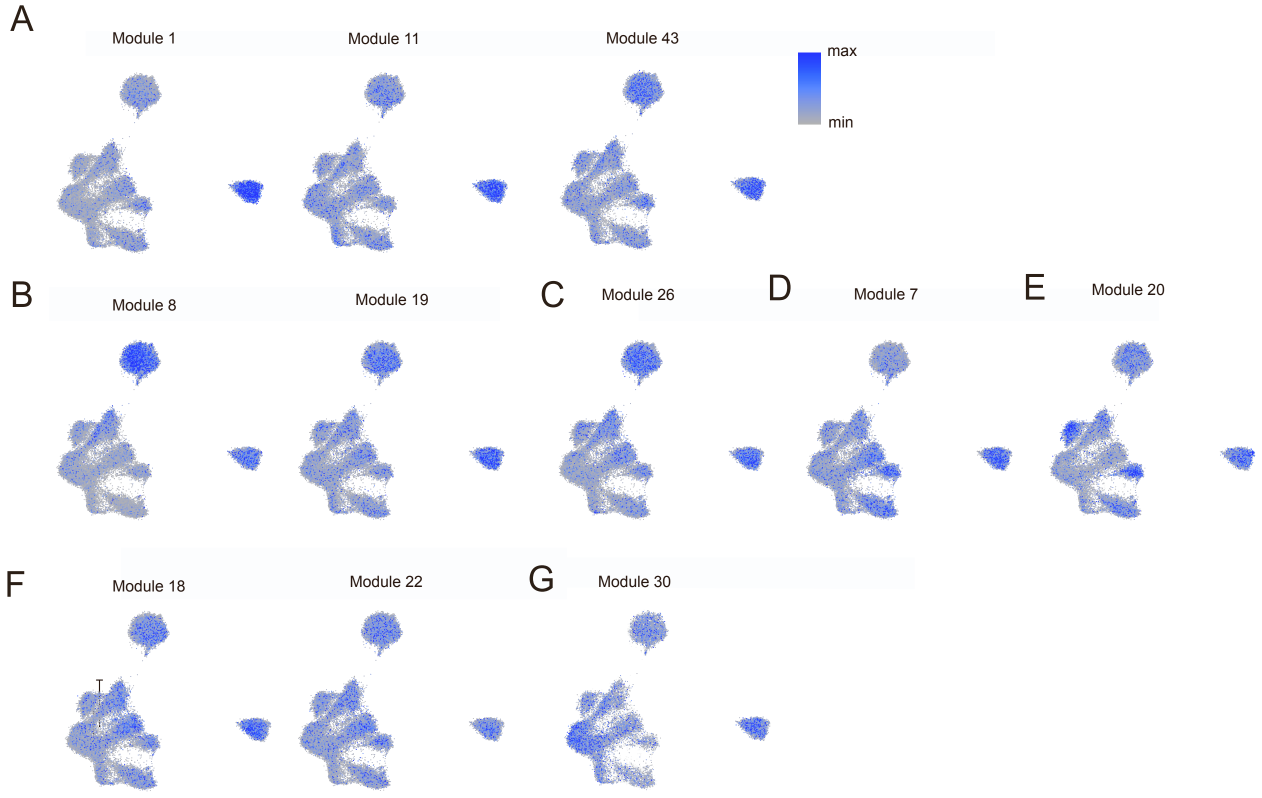


**Figure S3. Gene module analysis of ILCs, NK cells and T cells. Related to Figure 3.**

**(A-G)** UMAP illustration of the expression of selected gene modules shown in Figure 3. High expression of a gene module is shown as blue (max) and low expression of a gene module is shown as grey (min).

Figure S4

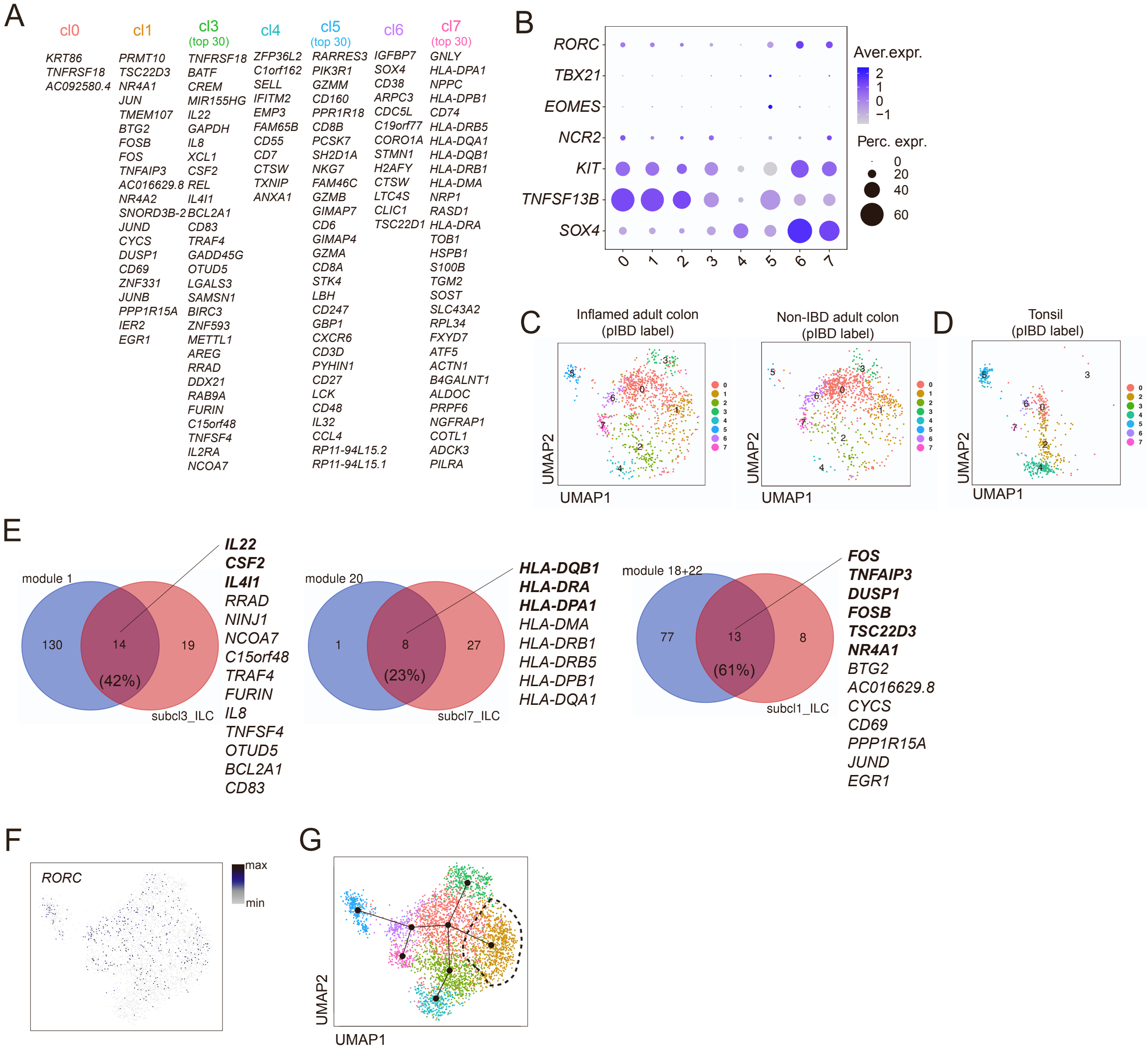


**Figure S4. Subcluster analysis of colonic ILCs. Related to Figure 4. (A)** Table showing the DEGs (log2fc > 0.10 and adj. p-value < 0.05) for each ILC subcluster (ordered from lowest to highest). For clusters 3, 5 and 7 the top 30 genes are shown. **(B)** Dotplot showing the expression of selected genes for the annotation of the ILC subclusters. **(C)** Inflamed and non-IBD ILCs from adult patients, projected onto the pIBD ILC UMAP shown in Figure 4A. **(D)** Tonsil ILCs from adult patients, projected onto the pIBD ILC UMAP shown in Figure 4A. **(E)** Venn diagrams showing the overlap between DEGs in ILC subcluster 3 (subcl3_ILC) and module 1 described in Figure 3C, ILC subcluster 7 (subcl7_ILC) and module 20 described in Figure 3G, ILC subcluster 1 (subcl1_ILC) and modules 18+22 described in Figure 3H. The overlapping genes in the intersection of the circles are indicated. In bold are the genes shown in Figure 4. The gene frequency is derived from the number of genes the ILC subclusters share with the modules (number in the intersection) divided by the total DEGs (log2fc > 0.10 and adj. p-value < 0.05) in each ILC subcluster. **(F)** UMAP

illustration of *RORC* expression in ILC subclusters. **(G)** Developmental trajectories of ILC subclusters performed with Slingshot. Subcluster 1 (dotted circle) was used as the root.

# Figure S5

A

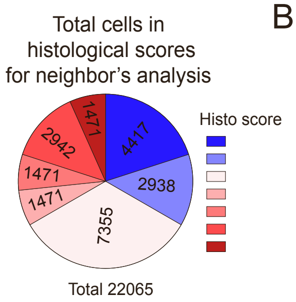


B

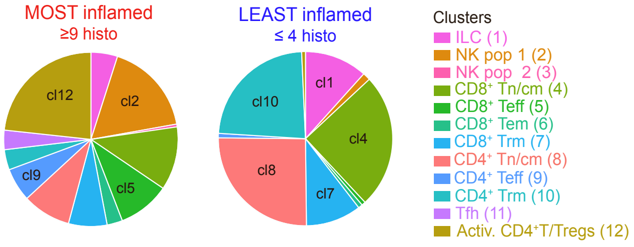


C

| Sample (histo score) | 1 (1) | 2 (3) | 3 (3) | 4 (7) | 5 (7) | 6 (10) | 7 (1) | 8 (10) | 9 (11) | 10 (12) | 11 (1) | 12 (11) |
|---|---|---|---|---|---|---|---|---|---|---|---|---|
| >9 histo score | | | | | | | | | | | | |
| cl1 | 0 | 0 | 0 | 0 | 0 | 0 | 0 | 0 | 64 | 26,8 | 2,9 | 5,9 |
| cl2 | 0,4 | 0 | 3,4 | 0,4 | 0 | 0,4 | 0 | 0,4 | 25 | 41 | 1,7 | 25,6 |
| cl3 | 0 | 0 | 0 | 0 | 0 | 0 | 0 | 0 | 14 | 85 | 0 | 0 |
| cl4 | 0 | 0 | 0 | 0 | 0 | 0 | 0 | 0 | 35 | 56 | 2,5 | 5,6 |
| cl5 | 0 | 0 | 0 | 0 | 0 | 0 | 0 | 0 | 55 | 42,7 | 0,7 | 1,5 |
| cl6 | 0 | 0 | 0 | 0 | 0 | 0 | 0 | 0 | 46 | 54 | 0 | 0 |
| cl7 | 0 | 0 | 0 | 0 | 0 | 0 | 0 | 0 | 52 | 43,2 | 1 | 3 |
| cl8 | 0 | 0 | 0 | 0 | 0 | 0 | 0 | 1 | 54 | 36,8 | 2,4 | 5,7 |
| cl9 | 0 | 0 | 0 | 0 | 0 | 0 | 0 | 0 | 41,6 | 53,5 | 2,3 | 2,3 |
| cl10 | 0 | 0 | 0 | 0 | 0 | 0 | 0 | 0 | 45 | 54,9 | 0 | 0 |
| cl11 | 0 | 0 | 0 | 0 | 0 | 0 | 0 | 0 | 305 | 42,8 | 2 | 24,4 |
| cl12 | 0 | 0 | 0 | 0 | 0 | 0 | 0 | 0 | 67 | 201 | 6 | 42 |
| <4 histo score | | | | | | | | | | | | |
| cl1 | 4,8 | 8,6 | 4,5 | 2,2 | 1,7 | 1 | 25,3 | 5,9 | 0,3 | 0 | 41,1 | 4,1 |
| cl2 | 37,1 | 30 | 1,4 | 1,4 | 0 | 0 | 21,4 | 0 | 0 | 0 | 8,5 | 0 |
| cl3 | 0 | 0 | 0 | 0 | 0 | 0 | 0 | 0 | 0 | 0 | 0 | 0 |
| cl4 | 40 | 37,2 | 5,5 | 7 | 1,2 | 3,8 | 1,8 | 1,2 | 0,008 | 0 | 1,1 | 0,6 |
| cl5 | 52 | 19,4 | 0 | 0 | 0 | 0 | 25 | 2,7 | 0 | 0 | 0 | 0 |
| cl6 | 35,8 | 28,2 | 5,1 | 0 | 0 | 2,5 | 2,5 | 5,1 | 2,5 | 0 | 15,3 | 2,5 |
| cl7 | 31,4 | 43,3 | 3,7 | 1,5 | 1,3 | 0,7 | 5,1 | 2,5 | 0 | 0 | 9,2 | 0,7 |
| cl8 | 47 | 26,1 | 8,7 | 3 | 1,1 | 2,1 | 1,9 | 0,9 | 0,1 | 0 | | 0,3 |
| cl9 | 9,7 | 17 | 0 | 0 | 0 | 0 | 41,7 | 2,4 | 0 | 0 | 39 | 0 |
| cl10 | 15,5 | 17,5 | 18,5 | 4,9 | 0,9 | 0,8 | 17,6 | 3,4 | 0,4 | 0,6 | 17,3 | 2 |
| cl11 | 0 | 0 | 0 | 0 | 0 | 0 | 0 | 0 | 0 | 0 | 0 | 0 |
| cl12 | 31,4 | 48,5 | 8,5 | 0 | 0 | 0 | 2,8 | 2,8 | 0 | 0 | 5,7 | 0 |

D

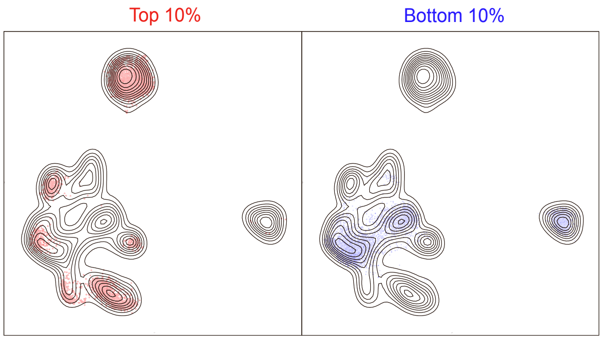


E

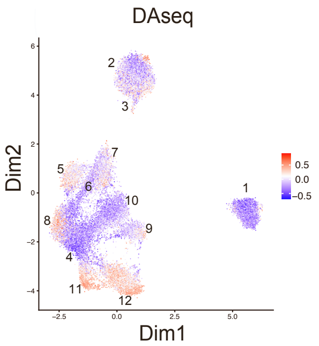


**Figure S5. Distribution of most- and least inflamed cells across cell clusters. Related to Figure 5. (A)** Pie chart showing the total number of cells in the histological scores post-sampling of the nearest-neighbor analysis shown in Figure 5. **(B)** Pie chart summaries showing the enrichment of most and least inflamed cells in the main UMAP clusters. **(C)** Table showing the percentage (%) contribution of each sample to cl1-12 of the >9 and <4 histo score UMAPs of Figure 5F. **(D)** Visualization of cells from the nearest-neighbor smoothing on the main UMAP with cells derived from top 10% (most inflamed) and bottom 10% (least inflamed) groups after Harmony batch correction. **(E)** Prediction UMAP plot from the DAseq analysis. Blue indicates cells with low histological score and red with high histological score.

**Table S1. Patient characteristics of pediatric IBD flow cytometry cohort. Related to Figure 1.**

***Pediatric IBD patients***

| | |
|---|---|
| N (total) | 19 |
| Diagnosis (% UC) | 37 |
| Diagnosis (% CD) | 63 |
| Age (years) mean; IQR | 14; 6 |
| Sex (% Female) | 58 |
| P-CRP (mg/L) range; median | 1-99; 1 |
| ESR (mm/h) range; median | 3-84; 26 |
| F-calprotectin (mg/kg) range; median | 519-5000; 1820 |
| S-albumin (mg/L) range; median | 22-44; 35 |
| B-Hb (mg/L) range; median | 52-149; 114 |
| n inflamed samples | 15 |
| Inflamed sampling location colon (N), descendens/ascendens/transversum/multiple locations/NA | 7/6/1/1/4 |
| Inflamed samples: Endoscopic score, range; median | 1-3; 2 |
| Inflamed samples: Histologic score, range; median | 4-13; 9 |
| n non-inflamed samples | 12 |
| Non-inflamed sampling colon location (N), descendens/ascendens/transverum/multiple locations/NA | 3/5/2/2/6 |
| Non-inflamed samples: Endoscopic score, range; median | 0-0; 0 |
| Non-inflamed samples: Histologic score, range; median | 1-5; 1 |

***Pediatric non-IBD controls***

| | | |
|---|---|---|
| N (total) | 10 | |
| Age (years) mean; IQR | 13; 3 | **p* = 0.4 (compared to IBD group) |
| Sex (% Female) | 70 | ***p* = 0.5 (compared to IBD group) |
| P-CRP (mg/L) range; median | 1-1; 1 | |
| ESR (mm/h) range; median | 2-20; 7 | |
| F-calprotectin (mg/kg) range; median | 9-1922; 112 | |
| S-albumin (mg/L) range; median | 39-48 (43) | |
| B-Hb (mg/L) range; median | 120-150; 134 | |
| Sampling location colon (N), descendens/ascendens/tranverse/multiple locations/NA | 1/6/1/2/0 | |
| Endoscopic score, range; median | 0-0; 0 | |
| Histologic score, range; median | 1-1; 1 | |

*Unpaired t-test
**Chi-square test

**Table S2. SWIBREG endoscopic scoring for UC and CD. Related to Figure 1.**

| | | | |
|---|---|---|---|
| **SWIBREG scoring for UC (=Mayo score)** | | | |
| 0 = remission | | | |
| 1= mild disease | Erythema | Decreased vascular pattern | Mild friability |
| 2 = moderate disease | Marked erythema | Lack of vascular pattern | Friability and erosions |
| 3 =severe disease | Spontaneous bleeding | Ulceration | |
| **SWIBREG scoring for CD** | | | |
| 0 = remission | | | |
| 1= mild disease | Small aphthous wounds on an otherwise normal mucosa | | |
| 2 = moderate disease | Erythema, edema | Lack of vascular pattern | Friability, no ulceration |
| 3 = severe disease | Marked erythema, edema | Lack of vascular pattern | Ulceration |

**Table S3. Patient characteristics of pediatric IBD scRNA-seq cohort. Related to Figure 2.**

| *Pediatric IBD patients* | |
|---|---|
| N (total) | 6 |
| Diagnosis (% CD) | 100 |
| Age (years) mean; IQR | 14; 3 |
| Sex (% female) | 33 |
| SES-CD, range; median | 8-28; 18 |
| P-CRP (mg/L) range; median | 5-99; 9 |
| ESR (mm/h) range; median | 7-74; 29 |
| F-calprotectin (mg/kg) range; median | 615-2750; 1435 |
| S-albumin (mg/L) range; median | 31-41; 37 |
| B-Hb (mg/L) range; median | 83-153; 130 |
| n most inflamed samples | 6 |
| Most inflamed sampling location colon (N), descendens/ascendens/transversum | 2/4/0 |
| Most inflamed samples: Endoscopic score, range; median | 2-3; 2 |
| Most inflamed samples: Histologic score, range; median | 3-11; 10 |
| n least inflamed samples | 6 |
| Least inflamed samples: Endoscopic score, range; median | 0-2; 0 |
| Least inflamed samples: Histologic score, range; median | 1-11; 2 |
| Least inflamed sampling location colon (N), descendens/ascendens/transversum | 2/2/2 |

**Table S5. Patient characteristics of adult IBD flow cytometry cohort. Related to Figure 5I.**

*Adult IBD patients*

| | | |
|---|---|---|
| N (total) | 8 | |
| Diagnosis UC (%) | 25% | |
| Diagnosis CD (%) | 75% | |
| Age (years) mean; IQR | 39; 24 | |
| Sex (% female) | 50 | |
| Sampling location colon (N), rectum/descendens/ascendens/transversum/NA | 3/4/0/0/1 | |

*Adult non-IBD controls*

| | | |
|---|---|---|
| N (total) | 8 | |
| Diagnosis (% colorectal cancer) | 100 | |
| Age (years) mean; IQR | 79; 10 | **p* = <0.05 (compared to IBD group) |
| Sex (% female) | 37 | ***p* = 0.6 (compared to IBD group) |
| Sampling location colon (N), rectum/descendens/ascendens/transversum/NA | 0/4/1/3/0 | |

*Unpaired t-test
**Chi-square test
